# Direct Photocatalyzed Hydrogen Atom Transfer (HAT) for Aliphatic C–H Bonds Elaboration

**DOI:** 10.1021/acs.chemrev.1c00263

**Published:** 2021-08-06

**Authors:** Luca Capaldo, Davide Ravelli, Maurizio Fagnoni

**Affiliations:** † Flow Chemistry Group, Van’t Hoff Institute for Molecular Sciences (HIMS), University of Amsterdam, Science Park 904, 1098 XH Amsterdam, The Netherlands; ‡ PhotoGreen Lab, Department of Chemistry, University of Pavia, Viale Taramelli 12, 27100 Pavia, Italy

## Abstract

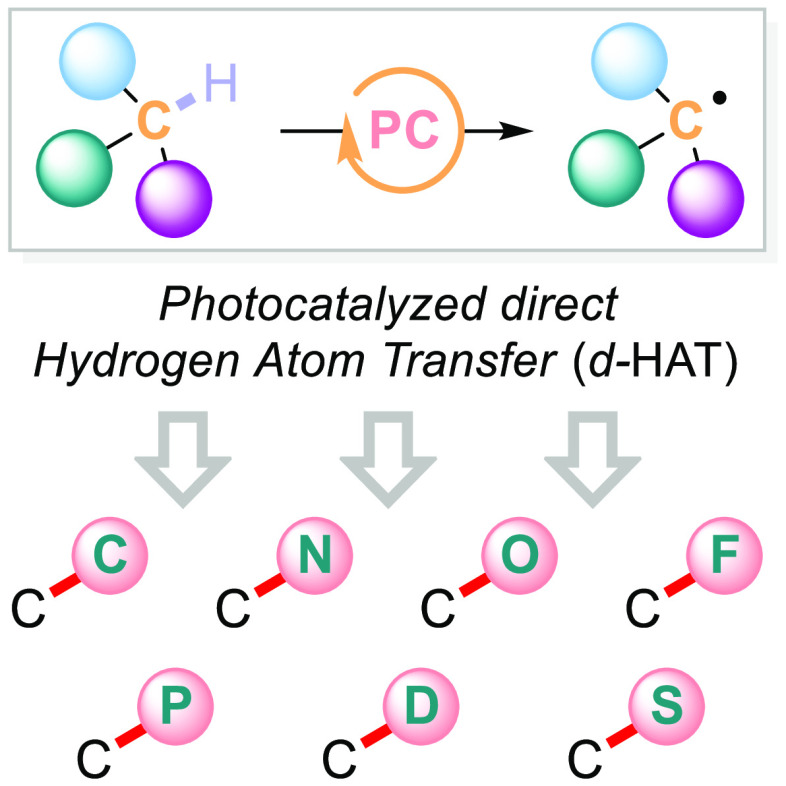

Direct photocatalyzed hydrogen atom transfer (*d*-HAT) can be considered a method of choice for the elaboration of aliphatic C–H bonds. In this manifold, a photocatalyst (PC_HAT_) exploits the energy of a photon to trigger the homolytic cleavage of such bonds in organic compounds. Selective C–H bond elaboration may be achieved by a judicious choice of the hydrogen abstractor (key parameters are the electronic character and the molecular structure), as well as reaction additives. Different are the classes of PCs_HAT_ available, including aromatic ketones, xanthene dyes (Eosin Y), polyoxometalates, uranyl salts, a metal-oxo porphyrin and a tris(amino)cyclopropenium radical dication. The processes (mainly C–C bond formation) are in most cases carried out under mild conditions with the help of visible light. The aim of this review is to offer a comprehensive survey of the synthetic applications of photocatalyzed *d*-HAT.

## 1 Introduction

The selective manipulation of C–H bonds (especially C(sp^3^)–H bonds) represents a remarkable challenge in synthetic campaigns because organic molecules contain many of these bonds of different nature. The acidity of hydrogens in the proximity of electron-withdrawing groups has been largely exploited for the smooth generation of enolates, versatile nucleophiles to forge C–C bonds.^1,2^ Apart from this fundamental reactivity pattern, aliphatic C–H bonds have been referred to as “unfunctional groups”^3^ due to their lack of reactivity and the more articulated strategies needed for their functionalization.^4^ Accordingly, the vast majority of C–H bond activation strategies in organic synthesis rely on the use of activating or directing groups, either to enable a particular reaction pathway or to improve selectivity as well as efficiency.^5^ Even though the use of temporary directing groups, that is functions that are reversibly bound to the substrate to drive selectivity, has been proposed,^6^ the direct aliphatic C–H bond elaboration in organic molecules still remains the unfound Holy Grail in chemistry.^3,7−12^ Notably, this is an intense area of research, because it is a godsend for late-stage functionalization^13−17^ and in function-oriented synthesis^18^ thanks to the innate atom-economy related to the direct elaboration of C–H bonds. Moreover, the selective activation of these bonds in structurally complex molecules is of immense value in medicinal chemistry,^14^ where small changes in a given structure may have a profound impact on its biological activity and in natural product synthesis.^19,20^

**Scheme 1:**
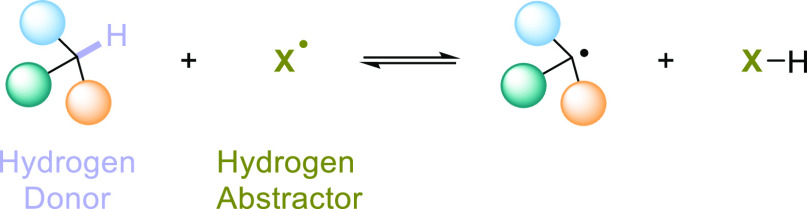
Homolytic Cleavage of a C–H Bond via a Hydrogen Atom Transfer Step

To address this challenging task, different metal-based strategies^4,19,21−24^ have been devised: in particular, Fe-,^25,26^ Cu-,^27^ Mn-,^28−30^ Co-,^31,32^ Rh-,^33^ Ir-,^34,35^ Ru-,^36−38^ and Pd-based^39−43^ catalysts have been successfully tested. Within this frame, one of the most appealing concepts consists in the homolytic cleavage of the C–H bond via a hydrogen atom transfer (HAT) event.^44−46^ This consists in the concerted movement of an electron and a proton (H^•^ ≡ H^+^ + e^–^) from the substrate, *aka* hydrogen donor, to an accepting species (a hydrogen abstractor); all in a single kinetic step (Scheme 1).

**Scheme 2:**
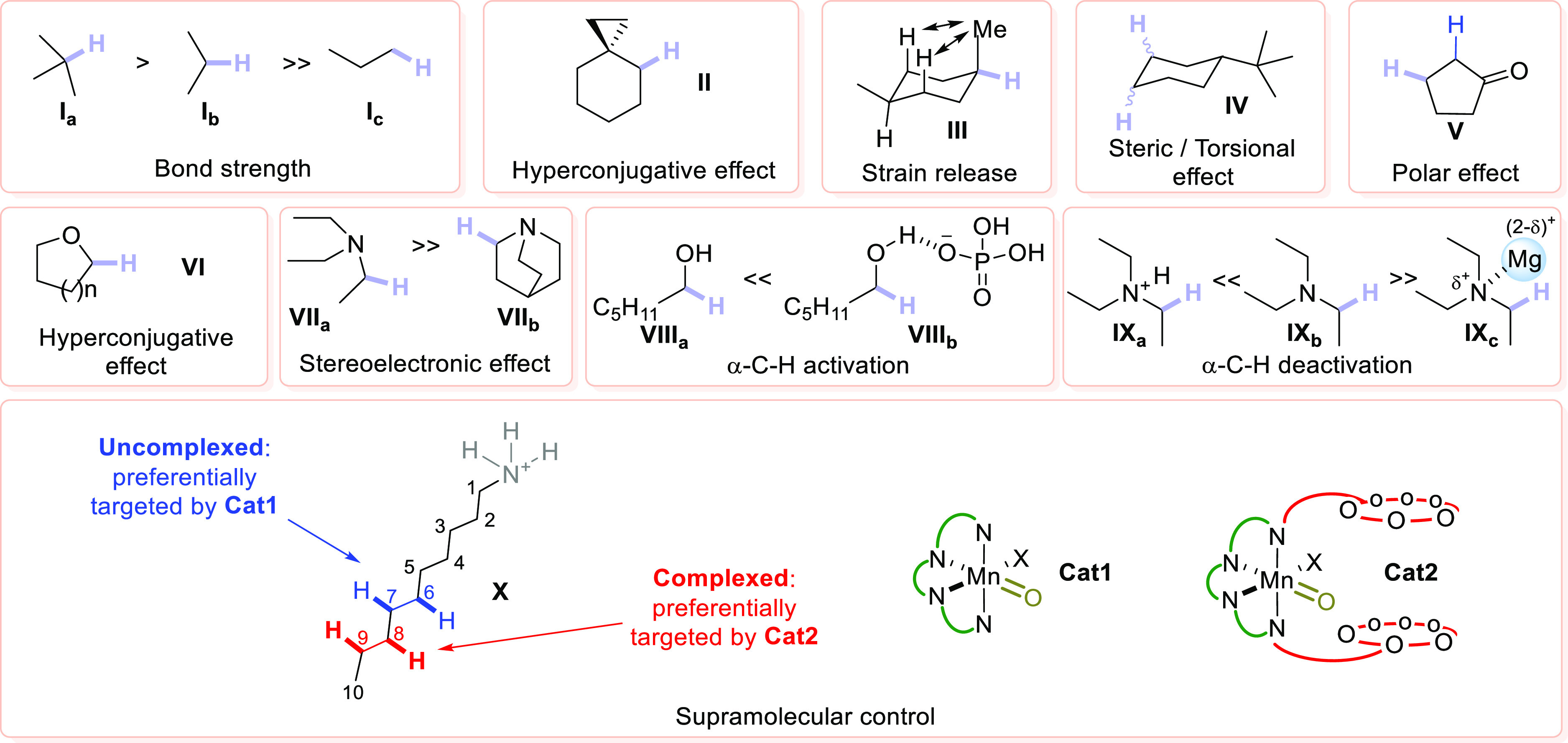
Factors Affecting C–H Bond Cleavage Different factors operating in the selective HAT-based C–H functionalization in organic compounds. In violet the reactive site where the hydrogen is preferentially cleaved during the functionalization (for the explanation of the effects, see the text).

The factors that rule this chemistry (and in general the approaches devoted to design a *selective* HAT) may be tentatively classified as depending on the substrate or on the hydrogen abstractor structures as well as “medium dependent”, as summarized in Scheme 2.

**Scheme 3:**
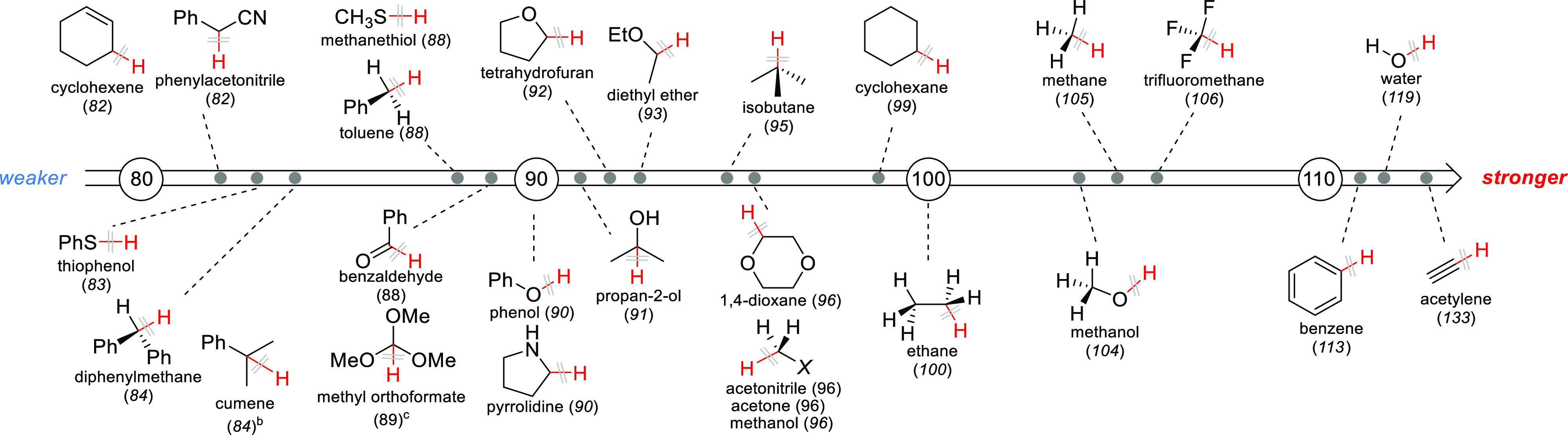
Bond Dissociation Energies (BDEs) in kcal/mol of Representative Compounds Bond dissociation energies (BDEs) in kcal/mol of the X–H bonds (in red) in representative compounds. Values taken from ref (53) except where otherwise noted. Value taken from ref (54). Value taken from ref (55).

One of the main effects belonging to the former class is the bond dissociation energy (BDE) of the C–H bond to be cleaved. In other words, the lower the BDE and the more stable the generated radical, the easier the bond to break (Scheme 3). However, this is just a rule of thumb and applies only under certain conditions. More often, other factors must be carefully evaluated to account for the difference in selectivity observed in the derivatization of a certain substrate (Scheme 2).^47−52^

Taking the case of hydrocarbons as reference, the relative stability of the generated organoradical drives the cleavage of a tertiary C–H bond (e.g., in compound **I**_**a**_) over that of a secondary (as in **I**_**b**_) or a primary C–H bond (in **I**_**c**_). Also, hyperconjugation and conjugation play an important role in steering the selectivity of HAT. For example, hyperconjugative effects may operate in the case of a hydrocarbon containing a cyclopropyl ring (**II**). In this case, the overlap between the C–C σ bonding orbital of the three membered ring and the neighboring C–H σ* antibonding orbital directs the C–H activation to the vicinal position (Scheme 2).^56−59^ In some instances, the strain release connected with the cleavage of a C–H bond is another driving force for a selective process. Indeed, this factor is rather significant for six-membered carbocyclic structures (see the case of compound **III**). In substituted cyclohexanes, the hydrogen abstraction from the equatorial C–H bond that is adjacent to a bulkier axial group leads to the release of 1,3-diaxial strain thanks to the planarization of the incipient carbon centered radical in the HAT transition state.^56,60−76^

While depending also on the nature/identity of the hydrogen abstractor, steric hindrance plays an important role as well, as known for the reactivity of *tert*-butylcyclohexane **IV**.^77^ This is another nice example of a “substrate-dependent” effect where the geometry of the molecule forces the activation toward precise reaction sites (e.g., positions 3 and 4) due to simple steric/torsional effects.^75,77^

Turning to substrates containing heteroatoms (e.g., O, N, etc.), the presence of polar functionalities may influence neighboring C–H bonds and their reactivity. Notably, the polarity match (or mismatch) between the character of the C–H bond to be cleaved and the hydrogen abstractor is another factor that hugely affects HAT. For example, an electrophilic species (e.g., an alkoxy radical or a related derivative) strongly prefers to abstract an hydridic (nucleophilic) C–H bond rather than an electron-poor (electrophilic) one of similar strength, a feature that is directly linked to the electronic character of the accessible radical intermediate.^78^ This allows the use of solvents having (rather labile) electrophilic C–H bonds (e.g., acetonitrile, acetone, Scheme 3), when an electrophilic hydrogen abstractor operates in the elaboration of nucleophilic C–H bonds.^45,79−82^

The polarity of C–H bonds in the substrate is thus influenced by the presence of electron-withdrawing or electron-donating groups.^56,60−66^ This is apparent in the case of cyclopentanone **V**, where the more labile electrophilic α-hydrogens are not activated by an electrophilic hydrogen abstractor since a partial positive charge on the incipient C-centered radical makes the corresponding transition state unfavorable. As a result, the β-C–H cleavage occurs instead.^83^

Another common “substrate-depending” effect governing HAT is that exerted by electron-donating functionalities, notably oxygen and nitrogen atoms. The donation of the nonbonding electrons by these atoms activates vicinal C–H bonds through hyperconjugation. Typical is the case of ethers (e.g., **VI**), acetals, alcohols, amines, and amides, where the heteroatom causes the decrease of the BDE values of the vicinal C–H bond via hyperconjugation and stabilizes the corresponding radical intermediate (see Scheme 2). Furthermore, the presence of heteroatoms may influence the selectivity via stereoelectronic effects,^56,60−66,84^ which allows rationalization of the different reactivities of open chain vs cyclic derivatives. By considering the cleavage of the α-to-N C–H bond in amines **VII**_**a**_ and **VII**_**b**_ as a representative example, the hydrogen abstraction in **VII**_**a**_ is more effective than in **VII**_**b**_. In fact, the process is more efficient when the bond being broken can be eclipsed with the heteroatom lone pair, not a favorable situation in **VII**_**b**_ due to the rigidity of the molecular scaffold.

Alternatively, “medium-dependent” effects (Scheme 2) can tune the reactivity pattern in chemical transformations occurring via HAT, again altering the reactivity of substrates containing heteroatoms through the activation or deactivation of the α-C–H bonds. Indeed, the solvent itself may function as hydrogen bond donor or acceptor due to its acidity or basicity;^85−89^ albeit, the presence of additives with peculiar acid/base properties may have a similar role.^90−106^ The activation effect is well illustrated by the selective C–H functionalization occurring in alcohol **VIII**_**a**_. Tetrabutylammonium dihydrogen phosphate forms a complex with the substrate via hydrogen bonding with the alcoholic O–H bond, thus increasing the n−σ* delocalization of the oxygen lone pair and making the α-to-O C–H bond more prone to a HAT process.^90^ On the other hand, a deactivation effect can be induced by an acidic solvent (e.g., a fluorinated alcohol) or by the addition of a Lewis or a Brønsted acid.^56,60−66,107−110^ Accordingly, both in the protonated form (**IX**_**a**_) and in the complexed form (**IX**_**c**_) the α-to-N C–H bond of triethylamine (**IX**_**b**_) is less prone to be cleaved due to the reduced availability of the nitrogen lone pair, and in some instances the selectivity is shifted to the β- (or, in general terms, remote) C–H bonds.^111^

Quite recently, supramolecular chemistry has been exploited to induce chemoselectivity in HAT-based processes.^112−114^ The ammonium salt **X** is functionalized by a Mn-oxo complex (**Cat1**) preferentially at C_6_ and C_7_. By employing a more sophisticated catalyst (**Cat2**) bearing two crown ether moieties able to complex the ammonium salt, it was possible to shift the functionalization toward C_8_ and C_9_.

Upon suitable conditions, a moiety embedded in the molecular scaffold can be activated and triggers the hydrogen abstraction at a specific site in an intramolecular fashion thus inducing a *remote* activation of a C–H bond (*r*-HAT).^41,115−122^ Typically, such site-selectivity is granted by the formation of a favorable six-membered cyclic transition state, which results in the occurrence of a 1,5-HAT step, despite the fact that the 1,n-HAT mode (*n* ≥ 6) may compete in some cases.^123^

**Scheme 4:**
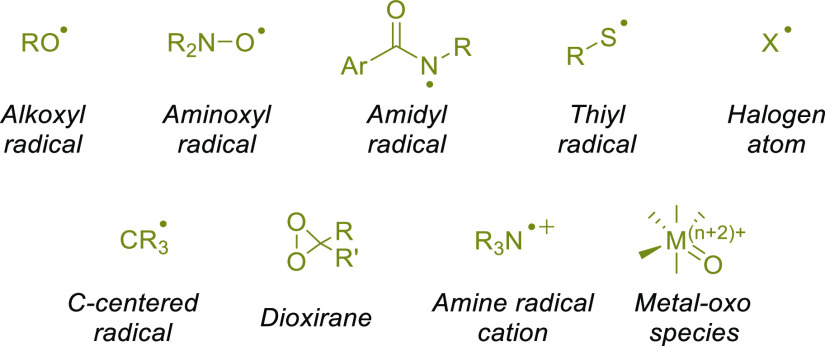
Common Hydrogen Abstractors Used in Synthetic Planning

Given the above, a synthetic route based on HAT has to be judiciously planned, starting from the choice of the proper hydrogen abstractor X^•^ (Scheme 4).^124^ Thermodynamics-wise, the newly formed X–H bond has to be stronger than the C–H bond to cleave to provide the driving force for the overall process, despite BDE not being the only parameter to be considered. As shown in Scheme 3, the BDE of α-to-heteroatom C–H bonds is mostly comprised between 85 and 95 kcal/mol, while primary and secondary C–H bonds in aliphatic hydrocarbons are quite strong (ca. 100 kcal/mol) calling for a highly reactive species (X^•^) to promote the cleavage event. Different hydrogen abstractors are known to have a radical or a radical-like character, including alkoxyl,^122,125−127^ aminoxyl,^62,128^ amidyl,^129,130^ and sulfonamidyl,^131^ azidyl,^132^ iodanyl,^133^ thiyl,^134−138^ and even C-centered^116,139−142^ radicals or halogen atoms,^143,144^ amine radical cations,^90,117,145−147^*N*-ammonium ylides,^148^ dioxiranes,^59,70,149,150^ or metal–oxo complexes.^29,151^ These hydrogen abstractors may be thermally or photochemically generated.

Recently, photocatalysis has emerged as a powerful synthetic platform in organic chemistry because it allows taming the tremendous amount of energy associated with light to build molecular complexity. It relies on the use of chemical species, namely photocatalysts (PCs), that can convert light into chemical energy for substrate activation.^152−180^

**Scheme 5:**
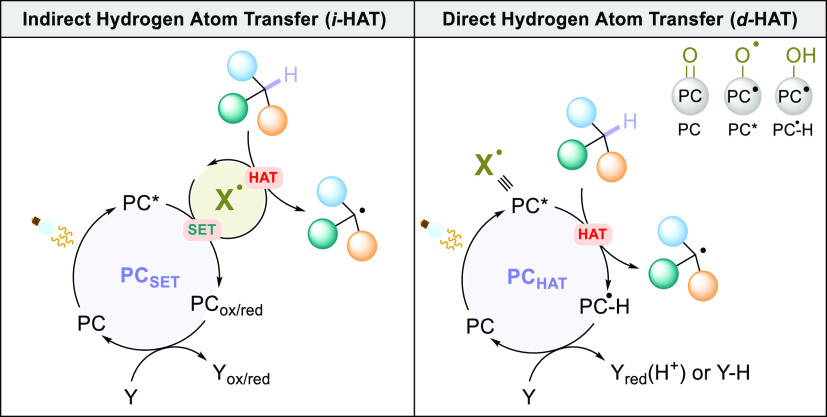
Photocatalyzed Indirect Hydrogen Atom Transfer (i-HAT) vs Direct Hydrogen Atom Transfer (**d-HAT**)

This methodology has been used to trigger HAT and, in particular, all the reports that appeared so far can be classified in two approaches: indirect hydrogen atom transfer (*i*-HAT) and direct hydrogen atom transfer (*d*-HAT, Scheme 5).

In the former case, the PC (PC_SET_) takes care of absorbing light and, once in the excited state, generates the hydrogen abstractor (X^•^, a radical or radical ion species) via a single-electron transfer (SET) step (Scheme 5, left).^181−184^ In the *d*-HAT process, the PC (PC_HAT_) triggers directly the HAT when in the excited state (Scheme 5, right).^181−185^ In other words, PC*_HAT_ coincides with X^•^.

A common structural motif to the vast majoriy of PCs_HAT_ currently known is the presence of an oxo group (Z=O), which acquires a peculiar *O*-centered radical character in the reactive excited state. The structure of the excited PC*_HAT_ strictly resembles electrophilic alkoxyl radicals (Scheme 5) behaving as excellent hydrogen abstractors to cleave a C–H bond in the chosen substrate. This leads to the formation of the (protonated) reduced form of the PC (PC^**•**^-H). At each catalytic cycle, the spent PC must be recovered back to its original state, so that it can promote over and over again the process, according to the definition of “photocatalyst” offered by the IUPAC: “*Catalyst able to produce, upon absorption of light, chemical transformations of the reaction partners. The excited state of the photocatalyst repeatedly interacts with the reaction partners forming reaction intermediates and regenerates itself after each cycle of such interactions*”.^186^ The actual mechanism of the PC restoration depends on the synthetic application and can involve a back-HAT step or a sequential electron/proton transfer (ET/PT) mechanism toward a chemical species (Y, Scheme 5) present in the reaction mixture (e.g., a sacrificial hydrogen acceptor) or transiently formed during the process, also dictating the overall redox balance of the synthetic transformation.^187^

**Figure 1:**
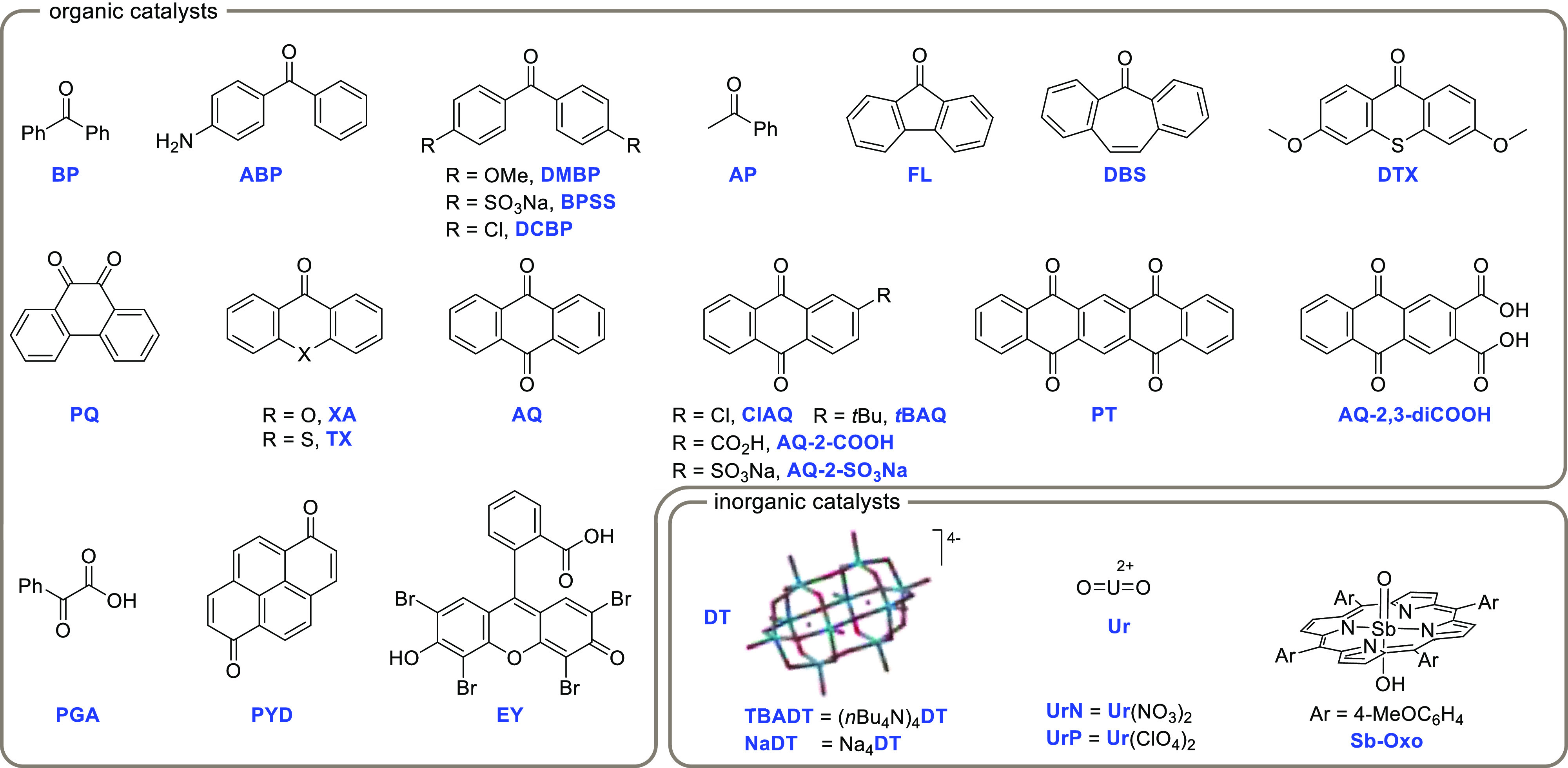
Main photocatalysts (PCs_HAT_) used in photocatalyzed **HAT: BP**, benzophenone; **ABP**, aminobenzophenone; **DMBP**, 4,4′-dimethoxybenzophenone; **BPSS**, disodium benzophenonedisulfonate; **DCBP**, 4,4′-dichlorobenzophenone; **AP**, acetophenone; **FL**, 9-fluorenone; **DBS**, dibenzosuberenone; **DTX**, 3,6-dimethoxy-9H-thioxanthen-9-one; **PQ**, 9,10-phenanthrenequinone; **XA**, xanthone; **TX**, thioxanthone; **AQ**, anthraquinone; **ClAQ**, 2-chloroanthraquinone; *t***BAQ**, 2-*tert*-butylanthraquinone; **AQ-2-COOH**, anthraquinone-2-carboxylic acid; **AQ-2-SO_3_Na**, anthraquinone-2-sulfonic acid sodium salt; **PT**, 5,7,12,14-pentacenetetrone; **AQ-2,3-diCOOH**, anthraquinone-2,3-dicarboxylic acid; **PGA**, phenylglyoxylic acid; **PYD**, 1,6-pyrenedione; **EY**, Eosin Y; **DT**, decatungstate anion (**TBADT**, tetrabutylammonium decatungstate; **NaDT**, sodium decatungstate); **Ur**, uranyl cation (**UrN**, uranyl nitrate hexahydrate; **UrP**, uranyl perchlorate); **Sb-Oxo**, antimony-oxo tetra-(*p*-methoxyphenyl)porphyrin.

Depending on the X-element carrying the oxo moiety, PCs_HAT_ can be grouped within different families (Figure 1). These comprise the class of carbonyl derivatives (Z = C),^188,189^ encompassing simple (aromatic) ketones and aldehydes,^190−192^ α-diketones,^193^ α-ketoacids,^194^ and (anthra)quinones,^195−197^ as well as the xanthene dye Eosin Y.^198,199^ On the other hand, inorganic derivatives including the decatungstate anion [W_10_O_32_]^4–^ (Z = W)^64,200−205^ and the uranyl cation [UO_2_]^2+^ (Z = U)^206,207^ as well as antimony oxo porphyrin complexes (Z = Sb)^208^ have been likewise proposed as PCs_HAT_. A notable exception of PC_HAT_ lacking the oxo group is known, namely the electrogenerated tris(amino)cyclopropenium (TAC) radical dication.^209^

Apart from structural similarities, the behavior of excited PCs_HAT_ featuring an oxo moiety shows many analogies. Thus, the capability of carbonyl derivatives, especially (aromatic) ketones, to act as hydrogen abstractor has been known for a long time.^296−301^ In particular, the photochemistry of these compounds is dominated by the triplet excited state and this is due to the very efficient intersystem crossing (ISC) from the first-formed singlet state. These triplet states have a lifetime in the microsecond range^302^ (Table 1) and, depending on the structure of the carbonyl derivative, may have a nπ* or a ππ* character (the former typically accountable for HAT reactivity).^296,303−309^ In carbonyl derivatives, the PC^**•**^-H form is a stable, long-living ketyl radical species (Scheme 5),^310^ featuring a very weak O–H bond (the calculated BDE of the O–H bond of the ketyl radical derived from acetone is ca. 16 kcal/mol).^311^ Accordingly, the restoration of the carbonyl group is the driving force for the recovery of the spent photocatalyst. However, a common drawback when using ketones as PCs_HAT_ is that ketyl radical may dimerize in solution to form pinacols thus decreasing the efficiency of the reaction.^312^ A particular case is that of the excited carbonyl of **EY** that has some phenoxyl radical character.^220^

**Table 1:** Photophysical Properties of Selected PCsHAT

Photocatalyst	λ_max_ (nm)	λ_use_ (nm)	Φ_ISC_	τ_T_ (μs)
**AP**	243, 279, 315 (THF or MeCN)^253^	19 W CFL^224^	∼1 (computational)^221^	6.6 (H_2_O/MeCN 9:1)^210^
**ABP**	337 (*i*-PrOH),^254^ 303 (C_6_H_12_),^254^ 308 (CCl_4_)^254^	365, UV LED^225^	0.82 (C_6_H_12_)^211^	3.6^211^
**AQ**	325, 370 (DCM)^255^	360,^226^ 390, 427^227^	0.95 (MeCN)^212^	1.62 (MeCN)^212^
**BP**	347, 415^256^	350,^228^ Hg lamp,^229−232^ 350 Rayonet,^233^ 366 Rayonet,^234^ 365 LED lamp,^235^ 18 W CFL,^236^ 400 nm LED^237^	1 (MeCN)^213^	19.6 (MeCN)^213^
**BPSS**	330^214^	315, 366^238^ sunlight^239^	1 (H_2_O)^214^	352 (H_2_O)^214^
**ClAQ**	256, 265, 274, 325 (MeOH)^257^	365 LED lamp,^240^ Xe lamp^241^		1150 (CF_3_CH_2_OH at 77 K)^215^
**DMBP**	339^258^	365 UV fluorescent lamp^242^		3.4–17.6 (CH_2_Cl_2_/CHCl_3_ 1:1 at 77 K)^216^
**DBS**	251, 306, 347 (C_6_H_12_),^259^ 253, 316, 326 (EtOAc),^259^ 253, 306, 347 (MeCN),^259^ 255, 305, 348 (*i*-PrOH),^259^ 255, 306, 349 (MeOH)^259^	CFL^243^		980 (C_6_H_12_, Ar),^217^ 890 (EtOH, Ar),^217^ 740 (MeOH, Ar),^217^ 650 (C_6_H_6_, Ar)^217^
**PQ**	268, 324, 500 (CHCl_3_),^260^ 319, 312, 503 (C_6_H_6_)^261^	Blue LED strip^244^	1^222^	2.2^218^
**PGA**	350 (dioxane)^262^	household lamps^245^	∼1^194^	8^194^
**DTX**	354 (MeCN)^219^	365 LED lamp^246,247^	0.93^219^	862 (MeCN)^219^
**EY**	541 (DMF)^263^	Blue LED,^248,249^ 460 Blue LED,^250^ 470 blue LED,^251^ 520 Green LED,^250^ Xe lamp (400 nm cutoff)^252^	0.32^223^	∼21^220^
**FL**	382 (C_6_H_12_),^287^ 380 (C_6_H_6_),^287^ 377 (MeCN),^287^ 379 (EtOH)^287^	Xe lamp,^271^ CFL lamp^272^	0.97 (MeCN)^213^	70 (MeCN)^213^
**NaDT/TBADT**	260, 323 (H_2_O),^273^ 272, 321^288^	BLB lamp,^273^ 310 Multilamp reactor,^274^ 366 LED,^275^ Xe lamp,^271^ Solar light,^276^ 390 LED^277^	0.5 (MeCN)^264^	50 (MeCN)^264^
**PT**	270, 343 (CHCl_3_)^289^	365, 425,^278^ 390, 427,^279,280^ 455^281^		
**PYD**	427 (MeCN/THF 1:1),^290^ 266, 277, 398, 424, 448 (ether)^291^	Blue LED^282^		
**Sb-Oxo**	440^265^	405, 455^265^		8 (MeCN/H_2_O 95:5)^265^
**TX**	380^219^	405 LED^271^	0.99 (MeCN),^213^ 0.76^267^	28 (MeCN),^213,266^ 760 ns (CD_3_CN),^219^ 45 (MeCN)^267^
**UrN**	414^292−294^	456,^283^ Blue LED^284^	1^270^	400 (80 K, MeOH)^268^
**XA**	338^295^	365 LED,^285^ CFL^272,286^	1 (MeCN)^213^	4.8–8.3 (MeCN)^213,269^

Another deeply studied family of PCs_HAT_ is that of inorganic polyoxometalate (POM) derivatives. The first reports describing a HAT reactivity upon excitation of these metal–oxygen clusters in the presence of organic substrates (mostly alcohols) dates back to the 1980s.^313−317^ It was soon realized that tungsten-based POMs, in particular **DT**, outperform all the other known POMs in terms of HAT reactivity,^200,318^ offering a catalytic tool for the elaboration of C–H bonds.^319−330^ A common occurrence in photocatalytic systems based on **DT** is the observation of a typical blue color of the PC^**•**^-H form ([W_10_O_32_]^5–^, either protonated or not)^331^ that accumulates in solution.^332^ The studies of **DT**-based systems by means of time-resolved spectroscopic techniques^333−339^ concluded that the state responsible for the HAT reactivity is a relaxed excited state (“**wO**” having a lifetime of 55 ns in acetonitrile),^333^ probably of triplet multiplicity,^340^ not directly accessible upon excitation (a so-called dark state).^341,342^ Theoretical simulations supported these experimental spectroscopic works.^264,343,344^

Turning to the uranyl cation, despite the very weak absorption in the blue region of the spectrum (ε ∼ 10 M^–1^ cm^–1^ at λ = 423 nm), visible light irradiation can be adopted to trigger its photochemistry. This transition has been proposed to populate a long-lived (μs range lifetime) state which contains an extremely reactive oxyl radical, well explaining the HAT reactivity.^207,270,345−349^

A partial oxyl radical character of the triplet excited state has been likewise postulated to be the active species in the hydrogen abstraction operated by **Sb-Oxo**.^265^ In the latter case, Sb^V^ (a high-valent oxidation state element of the *p*-block) is used in the dihydroxo form that contains two hydroxyl groups in the axial positions. Upon treatment with a base, one of the two hydroxy groups turns into the desired oxo moiety; the excited state of the so-generated oxo species was exploited for hydrogen abstraction.^265,350^ Even in this case the lifetime of the triplet involved is in the microsecond range (Table 1).

It is important to stress, however, that PCs_HAT_ may be engaged in photocatalytic processes different from HAT (mainly electron transfer, but energy transfer may not be excluded).^185,265,309,351^ Accordingly, the real mechanism has to be checked carefully to ascertain if a HAT process is involved rather than an electron transfer followed by proton transfer or even a proton-coupled electron transfer (PCET) mechanism.^162^

In view of the above, the aim of the present review is to offer an overview of the synthetic applications based on photocatalyzed *direct* HAT (*d*-HAT), wherein the excited PC_HAT_ triggers the HAT step. On the other hand, examples dealing with a photocatalyzed *indirect* HAT (*i*-HAT)^181−184^ or *remote* HAT (*r*-HAT)^183^ as well as the activation of C–H bonds via a PCET mechanism^352^ will be excluded. The threshold that we used throughout the entire work to consider an approach photocatalytic is 20 mol % of catalyst loading. We then considered photocatalytic HAT reactions where the generated radical must be incorporated in the desired compound, so the photogeneration of a thermally active redox agent will not be treated here.^290,353−355^ Photoinitiated processes wherein the light-absorbing species undergoes degradation during the process have been likewise excluded.^356−358^

Similarly, the adoption of a *d*-HAT strategy in polymerizations will not be mentioned; however, the reader is invited to refer to seminal works in the field.^359−364^ Thus, synthetic applications are preferentially treated here avoiding (when possible) the works simply devoted to mechanistic purposes and where the PC_HAT_ tested gave a very low yield or a very low reagent consumption.

The following sections have been organized based on the bond being formed during the transformation and found in the final product, while different types of transformations (e.g., dehydrogenation and fragmentation reactions) have been reported in the final part of the review. Thus, under the section “formation of a C(sp^3^)–C(sp^2^) bond”, examples wherein a C(sp^3^)-centered radical (formed from the photocatalyzed homolysis of a C(sp^3^)–H bond via a HAT step) will be attached to a C(sp^2^) atom in the final product will be described. Moreover, despite the fact that most of the examples reported in this review deal with the functionalization of C–H bonds, the elaboration of P–H, Si–H, and S–H bonds via a photocatalyzed *d*-HAT step has been mentioned, for the sake of comprehensiveness.

All the schemes have been color-coded so that the bond activated via HAT has been reported in violet, while the bond formed has been highlighted in red.

## 2 Formation of C–C Bonds

### 2.1 Formation of C(sp^3^)–C(sp^3^) Bonds via Addition onto C=C Bonds

A typical reactivity mode that can be exploited to forge a C(sp^3^)–C(sp^3^) bond is the radical addition of nucleophilic radicals onto Michael acceptors. In this scenario, the C-centered radical generated via photocatalyzed HAT is trapped by an electrophilic olefin and the resulting radical adduct is quenched via back hydrogen atom transfer (or sequential electron/proton transfer) from the reduced form of the photocatalyst (PC^**•**^–H; see Scheme 5), thus closing the photocatalytic cycle. This is a very reliable and general protocol as also demonstrated by the vast amount of hydrogen donors that can be profitably employed, with notable examples including alcohols, ethers, dioxolanes, sulfides, amides, nitriles, as well as simple hydrocarbons such as toluenes, allylated derivatives, and even (cyclo)alkanes.

#### 2.1.1 Oxygen- and Sulfur-Containing Compounds as Hydrogen Donors

**Scheme 6:**
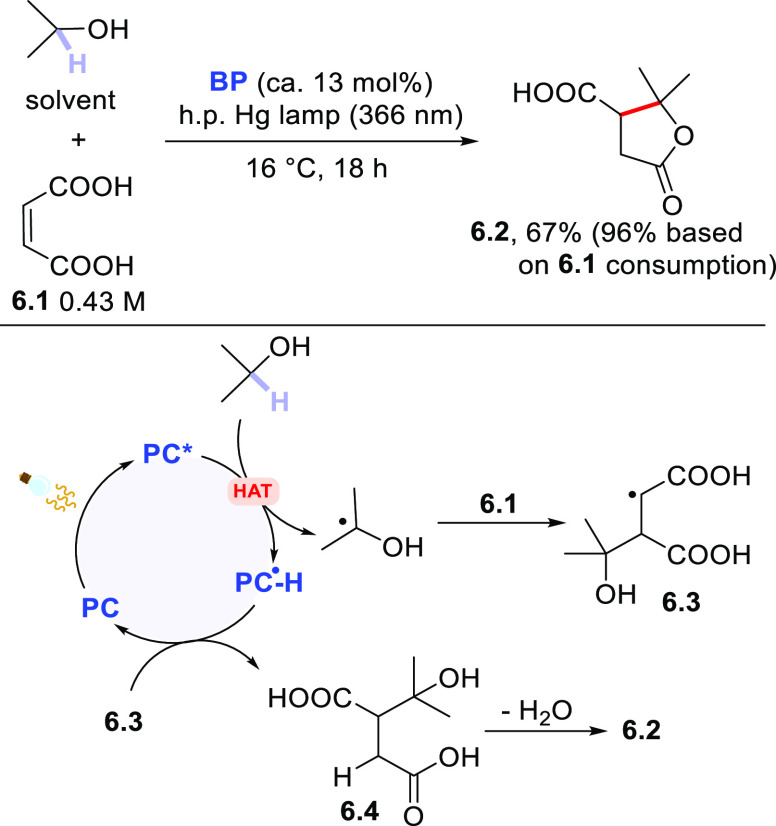
Photoaddition of Isopropanol onto Maleic Acid

Oxygenated derivatives have been the elective substrates for this reactivity manifold since the earliest reports on photocatalyzed HAT; this is because of the low BDE of the α-to-O C–H bond and the relatively stable α-oxy radical generated. In particular, to the best of our knowledge, alcohols are the first hydrogen donors ever investigated,^365^ and the earliest preparative example appeared in 1957 dealing with the photoaddition of isopropanol onto maleic acid **6.1** to give terebic acid **6.2** (Scheme 6).^366^ In those days, aromatic ketones (e.g., **BP**) were the elective class of PCs_HAT_ to perform the reaction under UV light coming from a Hg lamp.^366^ Due to the low cost of isopropanol, this was used as the reaction medium. The mechanism is depicted in Scheme 6 (lower part) and, as mentioned in the introduction to this section, it is quite common in all cases wherein a radical Michael addition takes place. Thus, the excited PC_HAT_ abstracts a hydrogen atom from the hydrogen donor and the resulting radical adds onto the olefin to give the radical adduct **6.3**. Back hydrogen atom transfer from the reduced form of the PC to **6.3** yielded the hydroxy acid **6.4** (and **6.2** from it by spontaneous lactonization) with the concomitant regeneration of PC_HAT_.

The same reaction was later on replicated on a gram scale by using the SOLFIN (SOLar synthesis of FINe chemicals) apparatus as solar light concentrator placed in Almeria (Spain).^239^ In this case, **BPSS** was used in the role of PC_HAT_, which was synthesized by sulfonation of parent **BP** (the sulfonation took place both at the 3- and 4-positions of the aromatic rings). The reason was that the thus-obtained PC_HAT_ was easily removed at the end of the reaction by extraction with water. Thus, ca. 14 g of terebic acid (**6.2**) was isolated in 75% yield upon 14 h solar light irradiation of an isopropanol/water 1:1 solution of **6.1** in the presence of 10 mol % **BPSS**.^239^ This PC_HAT_ has been likewise used to trigger the addition of alcohols (isopropanol, ethanol, and methanol) onto α,β-unsaturated aldehydes for the preparation of γ-lactols and γ-lactones upon treatment of the crude lactols with bromine.^238^ When maleic or fumaric acids were converted to the corresponding chiral (−)-menthyl diesters, the **BP** (19 mol %) photocatalyzed addition of isopropanol gave the acyclic diaterebic acid ester (63% yield) with a modest degree (8%) of diastereoselectivity.^367^

**Scheme 7:**
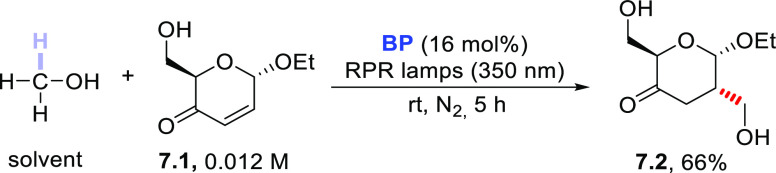
Photocatalyzed Functionalization of an α-Enone

Alcohols (in particular, methanol) were used to functionalize carbohydrate enones, such as hex-2-enopyranosid-4-ulose **7.1**, to form branched-chain monosaccharides (Scheme 7).^233,368−370^ Irradiation of this α-enone in MeOH in the presence of **BP** afforded 1,4-ketoalcohol **7.2** in 66% yield. Interestingly, the incorporation of the alcohol took place from the less-hindered side of the enone in a complete stereo- and regioselective fashion.^369^

**Scheme 8:**
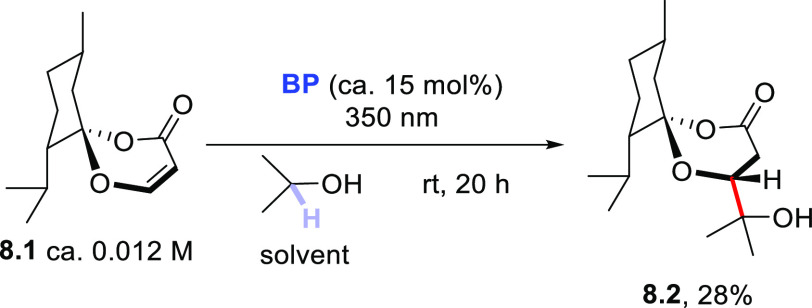
Photocatalyzed Synthesis of 1,5-Dioxaspiro[5.5]undecane-2-ones

Isopropanol was likewise used for the derivatization of 1,3-dioxin-4-ones having a (−)-menthone moiety embedded as chiral auxiliary in the 2-position (**8.1**, Scheme 8). The resulting 1,5-dioxaspiro[5.5]undecane-2-one (**8.2**) was formed, however, in a poor yield (<30% by using 15 mol % **BP**).^371^

On the other hand, the adoption of **TBADT** allowed the activation of isopropanol even by using a low amount of the PC_HAT_ (2–4 mol %) in the reaction with acrylonitrile (72% yield).^81,372^

**Scheme 9:**
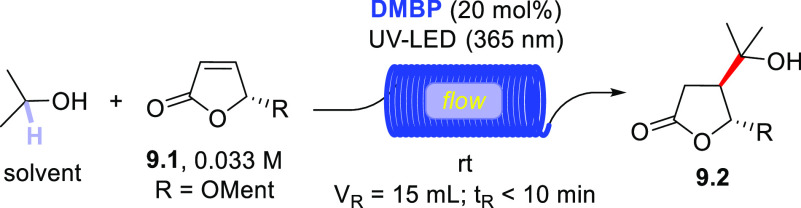
Photocatalyzed Functionalization of Furanones under Flow Conditions

The addition of isopropanol onto a Michael acceptor (e.g., furanone **9.1**, Scheme 9) was likewise carried out under flow conditions^373^ by using either an LED-driven microchip reactor,^374^ a continuous-flow photoreactor with parallel capillaries,^375^ or a multimicrocapillary flow reactor.^242^ In all cases, the adduct **9.2** was formed in a less than 10 min irradiation. Of note, **DMBP** was found to be the best PC_HAT_ among the several aromatic ketones tested.^242^

**Scheme 10:**
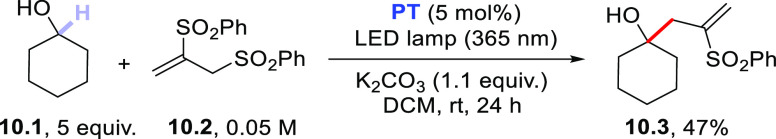
Synthesis of Homoallyl Alcohols

Cyclic alcohols have been rarely used, but the methine hydrogen atom in cyclohexanol (**10.1**, Scheme 10) was selectively abstracted by the excited state of **PT** and the radical formed was engaged in an allylation reaction to give homoallyl alcohol **10.3**.^289^

**Scheme 11:**
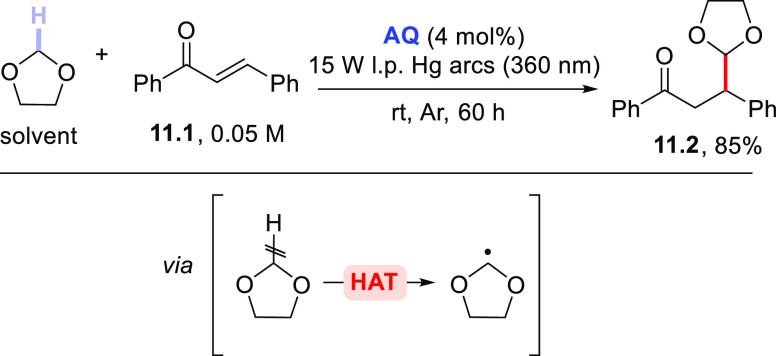
Photocatalyzed Incorporation of a Masked Formyl Group

The lability of the C–H bonds in position 2- in (2-substituted) 1,3-dioxolanes has been exploited for the generation of dioxolan-2-yl radicals, which moiety was used to formally introduce a masked formyl group. The reaction was initially tested on α-enones similar to **7.1** by using 1,3-dioxolane as the solvent.^233^ Later on, the process was extended to other enones, such as 1-phenyl-2-propen-1-one or chalcone **11.1** (Scheme 11).^226^ In that case, **AQ** was adopted as a visible-light-absorbing photoorganocatalyst (POC)^153^ and, despite the long reaction time needed (ca. 60 h), the final adduct **11.2** was isolated in 85% yield. The same reaction described in Scheme 11 was also performed in a 3D-printed, chemically resistant, nonswelling, and UV–vis transparent postfunctionalized flow reactor by using **ABP** as an immobilized PC_HAT_; however, an unsatisfactory yield (13%) was reported.^225^

The **BP**-photocatalyzed addition of 1,3-dioxolane onto 5-alkoxymethyl-2(5*H*)-furanone was used as the key step for the preparation of a bis-tetrahydrofuranyl ligand for HIV protease inhibitor UIC-94017 (TMC-114).^376^**BPSS** was likewise used as PC_HAT_ to promote the radical addition of 1,3-dioxolane onto α,β-unsaturated aldehydes to give 1,4-monoprotected succinaldehydes upon solar light exposure.^239^ The radical 1,3-dioxolanylation of alkenoic acids was also performed by using **DTX** (10 mol %) as POC.^247^ Shifting to a metal-based PC, the adoption of **UrN** allowed the hydrogen activation in 1,3-dioxolane for the addition onto Michael acceptors.^283^

Along the same line, 2-alkyl-1,3-dioxolanes were exploited as hydrogen donors for the (formal) incorporation of a ketone moiety, but they had to be used as cosolvents. In such a way, 1,4-monoprotected ketoaldehydes were obtained upon radical alkylation of α,β-unsaturated aldehydes.^238^

**Scheme 12:**
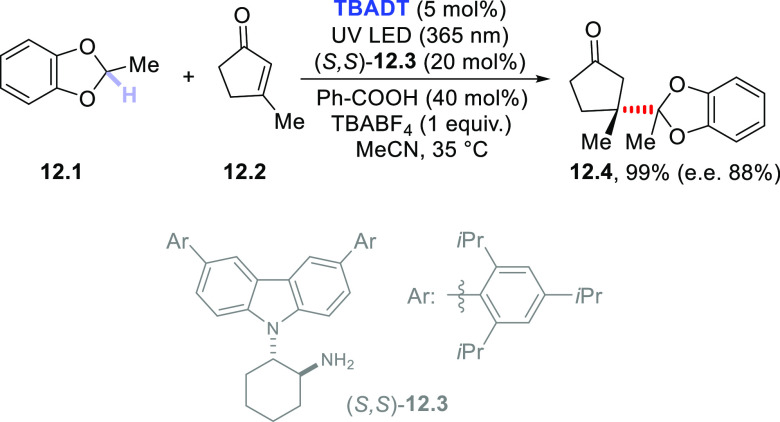
Dual-Catalytic Asymmetric Formation of Quaternary Carbons

**TBADT** was the elective PC_HAT_ for the activation of the methylene hydrogens in substituted 1,3-benzodioxoles (e.g., **12.1**) to give the corresponding 2-substituted derivatives by reaction with various Michael acceptors^377^ or with styrene^378^ (in the latter case in the presence of a disulfide cocatalyst). When the process was carried out on the β-substituted cyclic enone **12.2** (Scheme 12) in the presence of a chiral organocatalyst (i.e., carbazole derivative (*S,S*)-**12.3**), an enantioselective radical conjugate addition took place with formation of **12.4** in 99% yield. Notably, the latter product was formed with e.e. 88% and contains two quaternary carbon stereocenters.^379^ The reaction is based on an electron relay mechanism. In fact, the carbazole moiety is oxidized by an intramolecular electron transfer with the unstable radical cation formed by radical addition onto the chiral iminium ion intermediate, thus functioning as an electron donor.

**Scheme 13:**
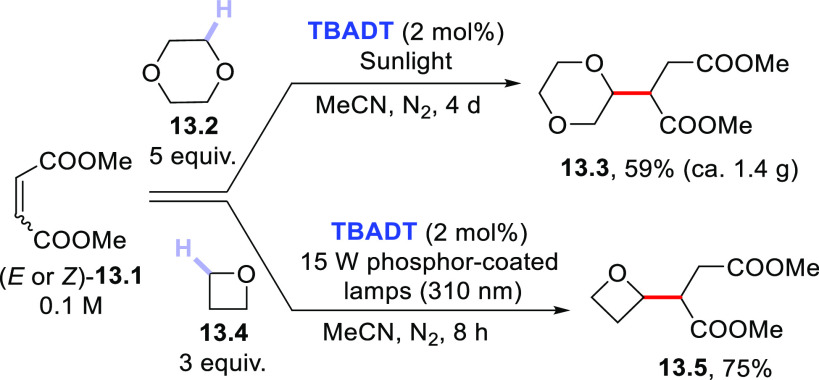
Cyclic Ethers as H-Donors in the Functionalization of Butendioate Esters

Another class of widely used oxygen-based hydrogen donors is that of cyclic ethers, wherein the HAT step occurs at the labile α-to-O C–H bonds. It is perhaps important to stress here that cyclic ethers cannot be easily activated otherwise; in fact, they are routinely used as inert solvents. Scheme 13 collects some representative examples concerning the derivatization of butendioate esters **13.1**. **TBADT** enabled the facile cleavage of the C–H bond adjacent to the oxygen atom both in 1,4-dioxane **13.2**(276) and in oxetane **13.4**.^380^ In the former case, sunlight was effectively used to irradiate the solution poured in a glass vessel placed on a window ledge. Despite the long time required (4 days), the reaction did not make use of any external source of artificial energy.^276^ The same process was performed upon UV light irradiation under flow conditions in a shorter period.^381^

**Scheme 14:**
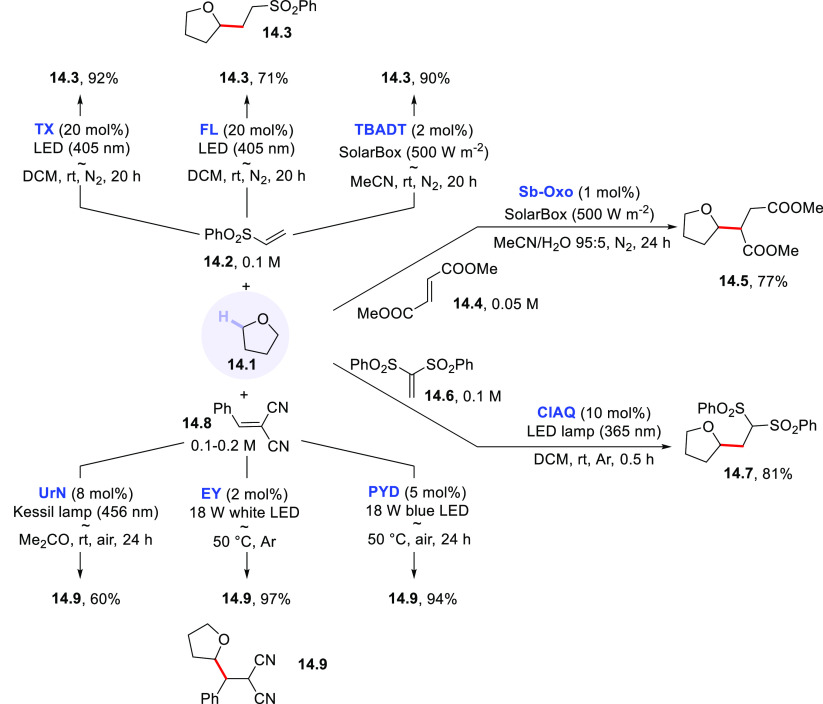
Different PCs_HAT_ for the Photocatalyzed Cleavage of the C–H Bond in THF

The generation of radicals from tetrahydrofuran (**14.1**) is useful to compare different PCs_HAT_ in their role and to stress the versatility of the photocatalyzed HAT process (Scheme 14). Thus, **14.1** may be photoactivated by having recourse to several PCs, including aromatic ketones such as **TX** and **FL** under visible light LED irradiation,^271^**TBADT** under solar simulated conditions,^271,382^ as well as with **Sb-Oxo**,^265^**UrN**,^283^**PYD**,^282^**EY**,^220^ and **ClAQ**.^240^ In all cases, satisfactory yields of adducts **14.3**, **14.5**, **14.7**, and **14.9** were obtained.

**Scheme 15:**
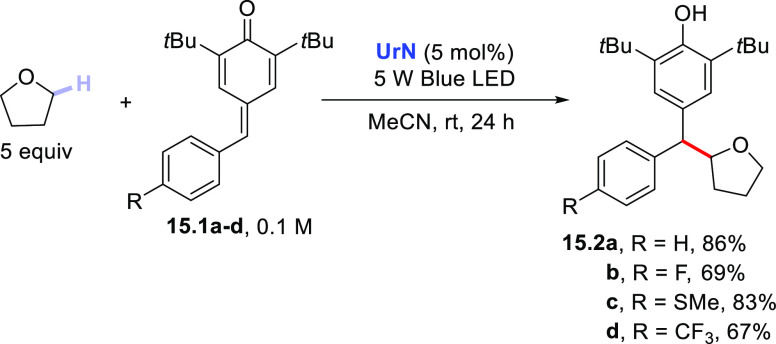
Photocatalyzed Addition of THF onto Cyclohexa-2,5-dien-1-ones

The photocatalyzed addition of THF was also applied to quinones **15.1a–d** (4-benzylidene-2,6-di-*tert*-butylcyclohexa-2,5-dien-1-ones, Scheme 15) under blue LED irradiation by using **UrN** (5 mol %) to give 2,6-di-*tert*-butyl-4-[phenyl(tetrahydrofuran-2-yl)methyl]phenols **15.2a–d**.^383^

**Scheme 16:**
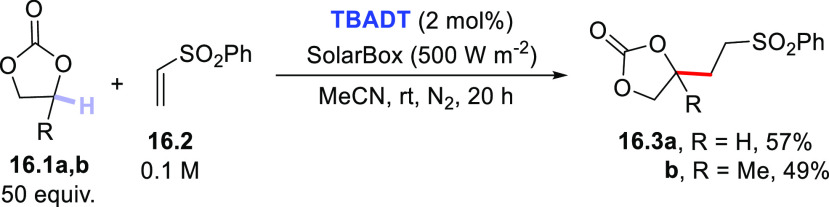
TBADT-Mediated Derivatization of Cyclic Carbonates

In rare instances, the activation of the C–H bond was applied to cyclic carbonates (**16.1a,b**, Scheme 16), where the presence of the carbonyl group did not hamper the C–H cleavage in these substrates. The importance of using **TBADT** is evident in this case, since the same process promoted by aromatic ketones gave no products **16.3a,b**.^271^ Introducing a methyl group in carbonate **16.1b** drove the cleavage to the most labile C–H bond present.

**Scheme 17:**
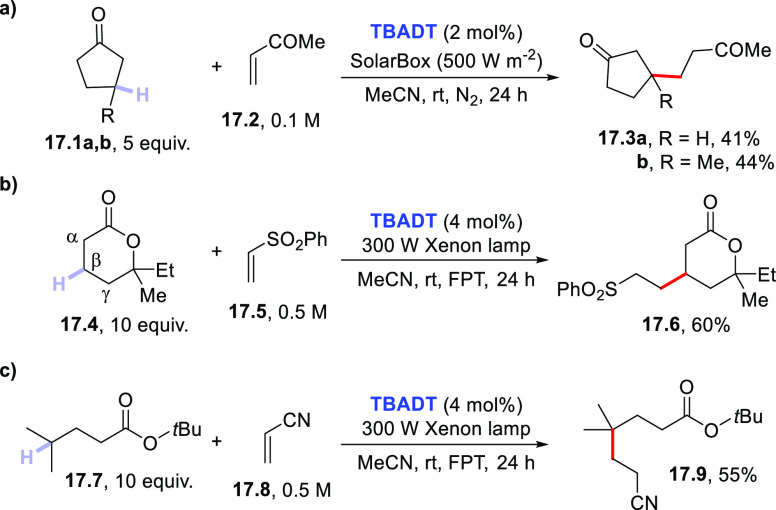
Selective C–H Cleavage in Ketones and Esters

Apart from the case of carbonates, other carbonyl-containing derivatives (ketones, esters, lactones, etc.) can be used as hydrogen donors. However, it is important to remember that in these cases the selectivity of the HAT step is shifted toward remote positions due to the mismatched polarity (Scheme 2). A typical case is the photoactivation of cyclopentanones **17.1a,b** (Scheme 17a). Despite the lability (and the acidity) of the α-C–H bonds with respect to β-C–H bonds in compound **17.1a**, the former are left untouched under the action of **TBADT** and a selective β-C–H to C–C bond conversion occurred.^83^ A more favorable polar HAT transition state has been invoked in this case to rationalize the observed regioselectivity.^83b^ The presence of a methyl group in compound **17.1b** made the methine hydrogen sufficiently labile to allow the preparation of compound **17.3b** as the sole product. Notably, the combination of polar and steric effects may direct the selective C–H cleavage in cycloalkanones and lactones. As an example, compound **17.4** underwent a selective β-C–H cleavage since both the hydrogen abstraction from the α-C–H and the γ-C–H bonds is prevented by polar and steric effects, respectively (Scheme 17b).^384^ In particular cases, the regioselective cleavage may be induced even in open chain esters (e.g., **17.7**) exploiting the lability of the methine hydrogen of the isopropyl group and taking advantage of the bulkiness of the *t*Bu group that prevents any other competitive C–H cleavage (Scheme 17c).^384^

**Scheme 18:**
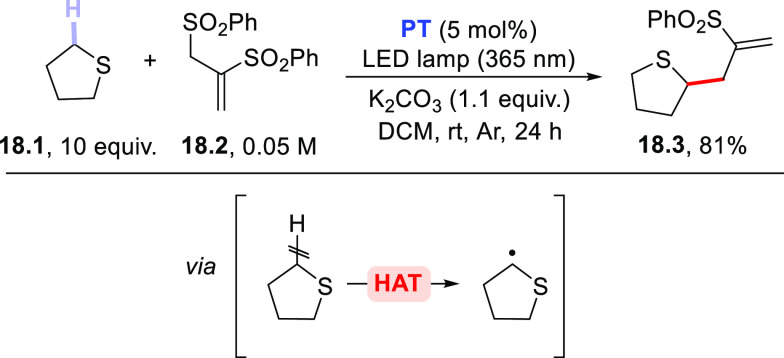
Allylation of Tetrahydrothiophene

Sparse examples have been reported involving the use of sulfides (mainly cyclic derivatives) as hydrogen donors. Thus, tetrahydrothiophene **18.1** (or thioxane) was allylated at the C–H bond adjacent to the *S*-atom by reaction with allyl sulfone **18.2** (Scheme 18).^289^ Despite the easy oxidizability of these sulfides, the adoption of **PT** avoided any competitive electron transfer reaction. Additionally, both **ClAQ**(240) and **EY**(220) were likewise effective PCs_HAT_ to trigger the C–H to C–C bond conversion in tetrahydrothiophene.

#### 2.1.2 Nitrogen-Containing Compounds as Hydrogen Donors

**Scheme 19:**
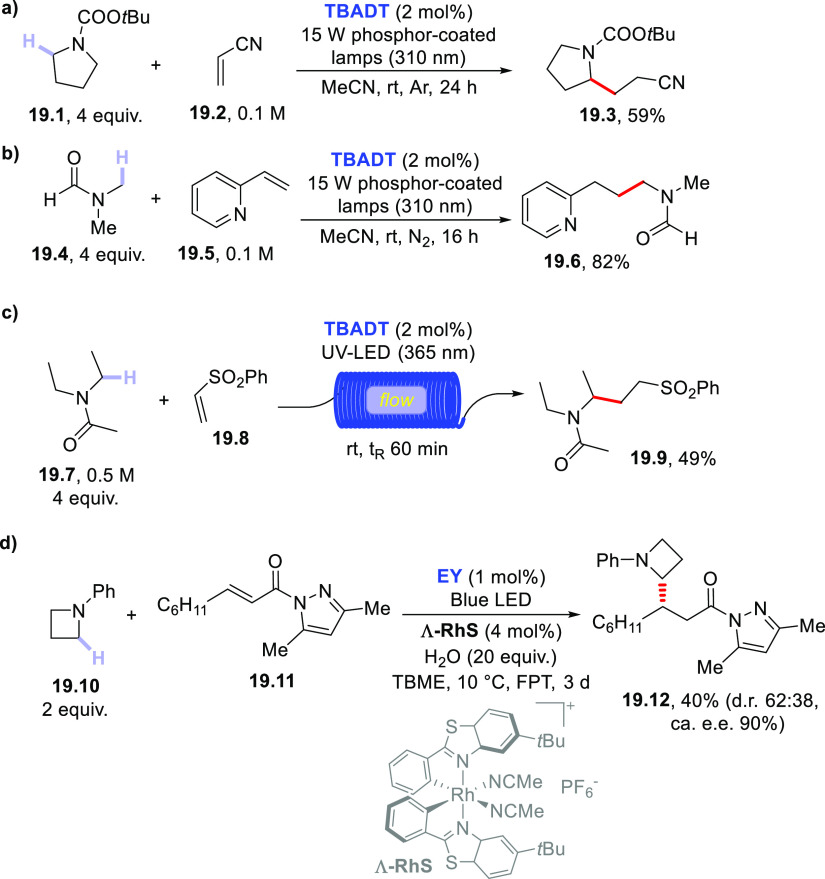
Amides, Carbamates, and Amines as H-Donors

A widely used class of nitrogen-containing hydrogen donors is that of amides (often used as the solvent) and carbamates; albeit, often the PC_HAT_ has to be used in a (super)stoichiometric amount for their activation.^157^ Nevertheless, catalytic amounts of **TBADT** smoothly promoted the C–H functionalization in amides and carbamates, used only in a 4 equiv excess (Scheme 19). The C–H bonds adjacent to the nitrogen atom in protected pyrrolidine **19.1** were sufficiently labile to be cleaved under photocatalyzed conditions to afford nitrile **19.3** (Scheme 19a), while excess **19.1** could be recovered during the purification.^385^ The reaction was found to be effective even under sunlight exposure.^276^ A similar C–H activation has been reported by using **ClAQ** (10 mol %)^240^ and **PT** (5 mol %)^289^ as PCs_HAT_.

As an alternative to typical Michael acceptors, the α-amidoalkyl radical formed from dimethylformamide **19.4** in the presence of **TBADT** was trapped by vinyl pyridine **19.5** to give adduct **19.6** in a very high yield (82%, Scheme 19b).^386^ It is important to note that in the latter case no C–H cleavage of the formyl hydrogen competed (see further section 2.5).

A related C–H functionalization of carbamates to perform a Giese-type alkylation was carried out by combining the action of a POC (**BP**, 20% mol) with a catalytic amount of Cu(OAc)_2_ (2% mol) under UV-A irradiation. In this case, the copper species prevents the otherwise feasible polymerization of Michael acceptors, such as unsubstituted acrylates, acrylonitrile, or methyl vinyl ketone.^387^

Even **EY** may be used for the selective, photocatalyzed addition of acetamide onto benzylidenemalononitrile.^220^ When using **UrN**, the C–H cleavage in **19.4** was not selective since competitive hydrogen abstraction from the C(sp^2^)–H bond took place (ca. 1/3 ratio).^283^ When *N*-methylacetamide was subjected to a hydrogen abstraction reaction by using **DCBP** (20 mol %), the resulting α-amidoalkyl radical was trapped by β-phenyl allyl sulfone to give the corresponding allylated derivative.^388^

The **TBADT**-photocatalyzed addition of tertiary amides (e.g., **19.7**, Scheme 19c) onto vinyl sulfones under flow conditions was selected for the easy preparation of γ-aminopropylsulfones (**19.9**).^389^ The latter conditions allowed scale-up of the process with a substrate concentration up to 0.5 M.^389^

In rare instances, an amine functioned as the hydrogen donor. Indeed, the electron-donor capability of such substrate may engage an electron transfer rather than a hydrogen atom transfer reaction (this is a typical case when using aromatic ketones).^185,351^ However, **EY** was able to functionalize amine **19.10** via enantioselective addition onto α,β-unsaturated *N*-acyl-3,5-dimethylpyrazole **19.11** (Scheme 19d).^390^ The asymmetric Giese-type addition of the photogenerated α-amino radical was promoted by the presence of the chiral rhodium Lewis acid catalyst **Λ-RhS**. As a result, adduct **19.12** was formed in a modest yield but with a high e.e..^390^

**Scheme 20:**
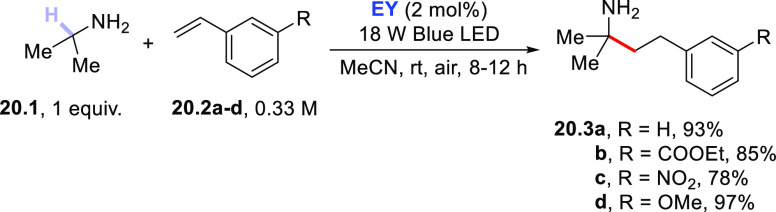
Photocatalyzed C(sp^3^)–H Alkylation of Amines

Similarly, in one instance a primary amine was derivatized under photocatalyzed conditions (Scheme 20). Thus, the visible light irradiation of a mixture of amine **20.1**, a styrene (**20.2a–d**), and a catalytic amount of **EY** caused the C–H cleavage of the methine hydrogen in **20.1**, finally affording the hoped-for 2-methyl-4-arylbutan-2-amine derivatives **20.3a–d**.^391^ Apart from the mildness of the reaction conditions, this is an important example dealing with the derivatization of vinyl aromatics.^391^

**Scheme 21:**
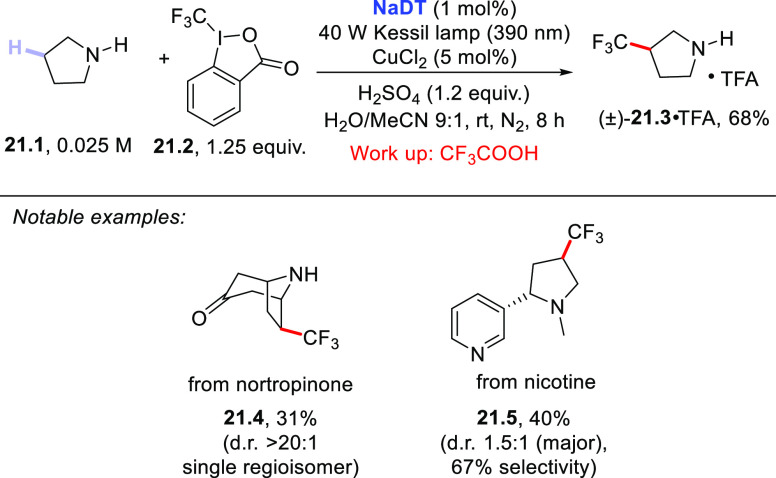
Metallaphotoredox Strategy for the Trifluoromethylation of Amines

A particular case in the activation of the C–H bond in amines is depicted in Scheme 21 and deals with the introduction of a trifluoromethyl group, which is known to improve the pharmacokinetic properties of drugs.^392^ This challenging C(sp^3^)–C(sp^3^) bond formation was made possible by merging **NaDT** chemistry with copper catalysis and made use of the Togni’s reagent **21.2** as the trifluoromethylating agent.^393^ The adopted acidic conditions here caused the formation of the ammonium salt of pyrrolidine **21.1**, thus deactivating the C–H bonds adjacent to the nitrogen atom (Scheme 2). Overall, the strategy is based on the addition of the photocatalyzed C-centered radical onto a Cu^II^–CF_3_ species. The hoped-for trifluoromethylated product **21.3** (trifluoroacetate salt) was then obtained in 68% isolated yield as a single regioisomer. The same procedure enabled the trifluoromethylation of benzylic C–H bonds and of biologically valuable compounds such as lidocaine, prilocaine, celecoxib, and torsemide. Mechanistic studies are consistent with the involvement of a “Cu-CF_3_ complex”.^393^

**Scheme 22:**
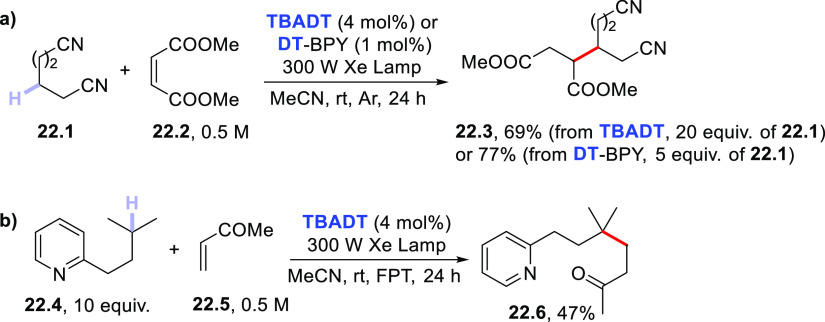
Regioselective Photocatalyzed C–H Cleavage in (a) Aliphatic Nitriles and (b) Alkylpyridines

Other nitrogen-containing hydrogen donors have been reported, such as aliphatic nitriles and alkylpyridines, where the influence of the heteroatom is not so important as in the previous cases; albeit, it still has a role in directing the C–H cleavage event. A representative case is that of adiponitrile **22.1** (Scheme 22a). The electron-withdrawing effect of the cyano group hampers the cleavage of α-C–H but not of β-C–H bonds. Thus, the **TBADT**-photocatalyzed reaction between **22.1** and dimethyl maleate **22.2** easily gave tetrafunctionalized adduct **22.3** in a satisfying yield.^394^ Related reactions involve the photocatalyzed addition of 4-methylpentanenitrile to phenyl vinyl sulfone (under flow conditions)^381^ or to a vinylpyridine.^386^

The same site-selective C–H to C–C bond conversion in nitriles took place when **DT** was incorporated within the pores of a copper-based metal organic framework (MOF) ([Cu_4_(BPY)_6_Cl_2_(W_10_O_32_)]·3H_2_O; **DT**-BPY, BPY = 4,4′-bipyridine). This is a rare case where the HAT process is carried out under heterogeneous conditions. The new PC_HAT_ showed high catalytic efficiency, high stability, and good recyclability, allowing use of a lower excess of the aliphatic nitrile substrate (only 5 equiv), thus improving the sustainability of the process (Scheme 22a).^395^

The C–H activation in alkylpyridines is interesting, since the labile benzylic hydrogens are not involved in the process, at variance with the alkylbenzene counterparts (see also section 2.1.3). This is well exemplified by the case of **22.4**, wherein the methine hydrogen was selectively cleaved and the resulting tertiary radical was then trapped by ketone **22.5** to give adduct **22.6** (Scheme 22b).^396^ In the last case, preference of the excited PC_HAT_ to abstract the less acidic (or, in other words, the less electrophilic) hydrogen atom in the investigated alkylpyridine was observed.

#### 2.1.3 Hydrocarbons as Hydrogen Donors

In hydrocarbons, it is possible to find quite labile hydrogens that can be easily cleaved under photocatalytic conditions. Hydrocarbons displaying labile benzylic^397^ and allylic C–H bonds can be easily cleaved at these sites under photocatalytic conditions. In fact, the BDEs of the most labile C–H bonds in toluene and cyclohexene are 88 and 82 kcal/mol, respectively (see Scheme 3).

**Scheme 23:**
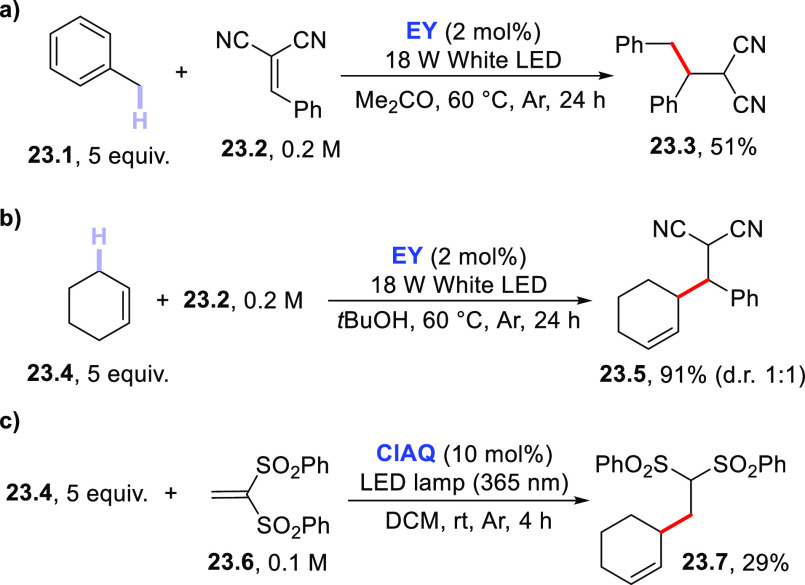
Functionalization of (a) Benzylic and (b,c) Allylic Hydrogens

The main problem here is the high stability of the radical formed and its reluctancy to react with the reaction partner (e.g., a C=C bond) to forge a C(sp^3^)–C(sp^3^) bond. This probably explains why very few processes involving these substrates have been reported. Simple alkylaromatics have been derivatized by using **TBADT** to perform valuable benzylations. However, only easily reducible olefins, including fumaronitrile, maleic anhydride, and substituted maleic imides, gave good results.^398^ The same conjugate radical additions were carried out in a mesoscale flow photoreactor by adopting a water-cooled 500 W medium-pressure Hg-vapor lamp as the light source. The use of this apparatus led to a marked increase of the STY (space time yield) and a reduction of the irradiation time compared with the same processes developed under batch conditions.^381^ Other PCs_HAT_ were likewise useful for this C–H activation strategy, as collected in Scheme 23. Thus, the benzylic position in toluene **23.1** was functionalized under **EY** photocatalysis despite heating at 60 °C being required (Scheme 23a).^220^ The activation of allylic hydrogens was also attempted by using **DT**, albeit not on a preparative scale.^200^

More recently, cyclohexene has been used as the hydrogen donor for the preparation of allylated derivatives **23.5** and **23.7 (**Schemes 23b,c). Both **EY**(220) and **ClAQ**(240) were employed in the functionalization of very good Michael acceptors **23.2** and **23.6**.

**Scheme 24:**
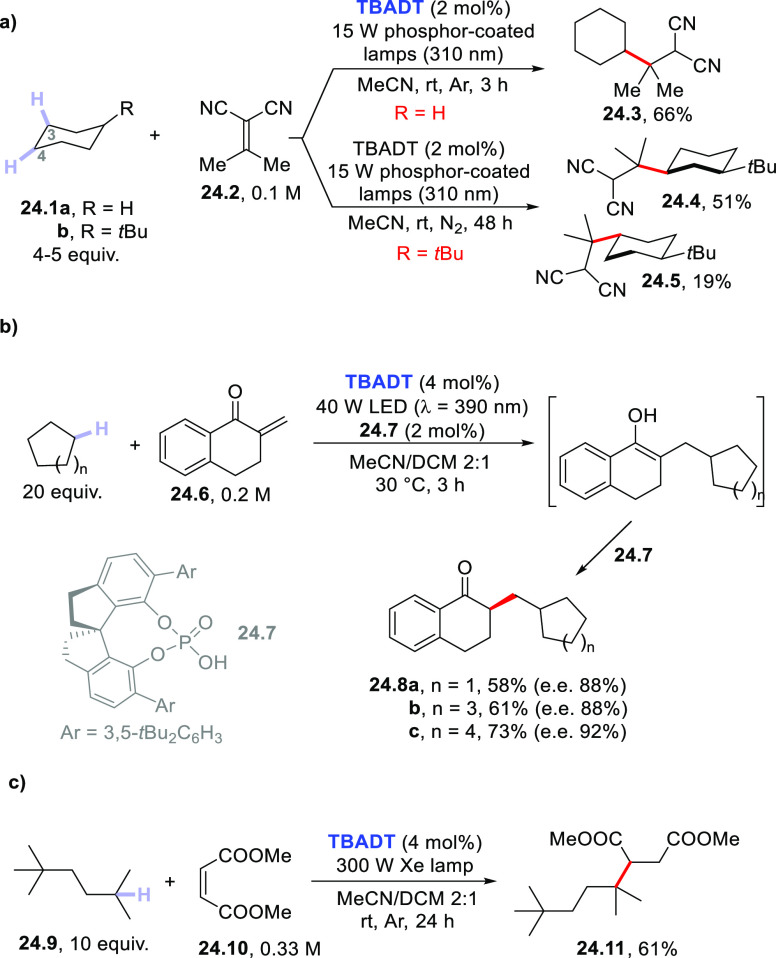
Photocatalyzed C–H Cleavage in (Cyclo)alkanes

The most challenging reaction for the construction of C(sp^3^)–C(sp^3^) bonds is related to the functionalization of (cyclo)alkanes,^124^ due to the high BDE of the C–H bonds involved (ca. 100 kcal/mol, see Scheme 3). Early photocatalytic experiments made use of a high amount of the PC_HAT_ to pursue this issue,^157^ but the use of **DT** allowed performing a real photocatalyzed process with only a few mol % loading of the PC_HAT_. Simple symmetric cycloalkanes were the preferred substrates.^81,274,399^ As shown in Scheme 24a, cyclohexane **24.1a** easily gave access to the corresponding cycloalkyl radical that was in turn trapped by dinitrile **24.2** to give **24.3** through a C–C bond formation step.^274^

Similarly, various 5- to 12-membered cycloalkanes were used to functionalize conjugated enones (**24.6**)^400^ even with the help of a chiral spiro phosphoric acid (**24.7**) to promote an asymmetric C–H functionalization (Scheme 24b).^401^ A chiral phosphoric acid similar to **24.7** was likewise adopted as a chiral proton-transfer shuttle in the cycloalkane addition onto α-substituted acrylates (e.g., *N*-acyl dehydroalanine benzyl esters) used as Michael acceptors for the smooth forging of enantioenriched α-stereogenic esters.^402^

Methylene norbornanone was alkylated in a good yield by using cyclohexane as the hydrogen donor under **UrN** photocatalysis.^283^ Similarly, the activation of nonacidic C(sp^3^)–H bonds in cyclohexane (or adamantane) was carried out upon UV light irradiation (**ClAQ** as the PC_HAT_) by using 1,1-bis(phenylsulfonyl)ethylene as the radical trap.^240^ The allylation of alkanes has been performed by means of the **PT**-photocatalyzed addition of cyclohexane, cyclododecane, or adamantane onto 1,2-bis(phenylsulfonyl)-2-propene as the allyl source.^289^

In rare instances, the reaction was applied to substituted cycloalkanes. Thus, the presence of a *t*Bu group in compound **24.1b** exerted a profound effect in steering the hydrogen abstraction process. In fact, the bulkiness of the *t*Bu group completely shielded the hydrogens in positions 1- and 2-, allowing the selective C–H cleavage in positions 3- and 4- (Scheme 24a). The bulkiness of the PC_HAT_ and the radical trap **24.2** further helped in reducing the number of possible isomers formed, with only *cis*-3-substituted **24.4** and *trans*-4-substituted **24.5** formed in an overall 70% yield.^77^ Interestingly, in the latter case, when **BP** (1 equiv) was used in place of **TBADT**, the same product distribution was roughly observed.^77^ However, when alkane **24.1b** reacted with acrylonitrile (**TBADT** as the PC_HAT_), a more complex mixture resulted.^77^

Open-chain alkanes were poorly investigated. A rare case is that reported in Scheme 24c. Despite the fact that compound **24.9** has five different types of hydrogen atoms, only the methine C–H position was effectively cleaved, and the reaction with maleate **24.10** led to diester **24.11** as the sole product.^372^ Even in this case, the bulkiness of the *t*Bu group helped in the regioselective cleavage of the C–H bond.

**Scheme 25:**
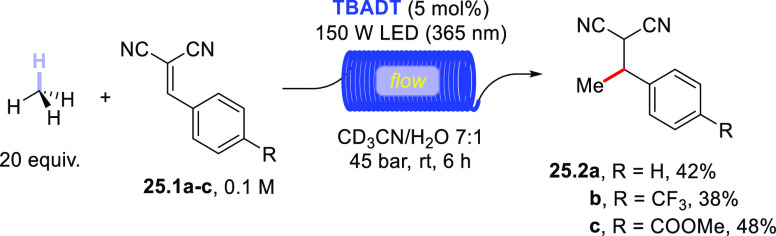
Photocatalyzed Derivatization of Methane under Flow Conditions

The activation of methane (BDE = 105 kcal/mol) was proved to be feasible by adopting **DT** photocatalysis. The process required specifically optimized conditions, namely the adoption of flow conditions and application of a high pressure (45 bar), to allow the correct mixing of the reagents (Scheme 25). Unfortunately, the C–H bond was so reluctant to undergo cleavage that acetonitrile competed in the HAT event. Accordingly, during the alkylation of dinitriles **25.1a–c**, a deuterated acetonitrile/water 7:1 mixture was mandatory to obtain a decent yield of methylated derivatives **25.2a–c**.^403^ The functionalization of ethane and propane was likewise carried out under milder conditions, with no need of deuterated solvents.^403^

**Scheme 26:**
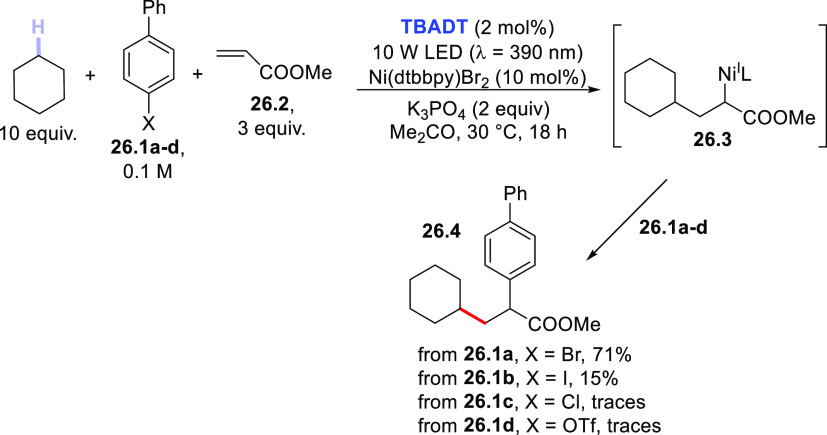
Photocatalyzed Three-Component Difunctionalization of Alkenes

The addition of an alkyl radical onto an electron-poor olefin may ultimately lead to difunctionalization of the double bond thanks to a dual-catalytic approach, as shown in Scheme 26. **TBADT** was again used as PC_HAT_ to generate an alkyl radical from cyclohexane. In this case, however, the adduct radical formed by addition of the cyclohexyl radical onto acrylate ester **26.2** was intercepted by a Ni^0^ catalyst to form the alkyl-Ni^I^ intermediate **26.3**. Oxidative addition of selected aryl derivatives **26.1a–d** onto **26.3** led to ester **26.4** in variable amounts depending on the leaving group X on the aromatic ring, with the bromine atom being the best choice. This approach showed a broad substrate scope since it may be applicable to several functionalized tertiary, secondary, and primary alkyl radicals.^404^ A related approach was likewise devised by combining a POC (**BP**) with the same [Ni(dtbbpy)Br_2_] catalyst.^405^

Another recent example where HAT catalysis was merged with metal catalysis involved a Pd-catalyzed allylic alkylation. In this strategy, the alkyl radical was trapped by a Michael acceptor and the resulting adduct radical was reduced and the resulting carbanion interacted with *in situ* formed π-allylpalladium species that finally released the desired allylation product.^406^ The approach developed was then used for the concise synthesis of (±)-mesembrine.^406^

As a final note to this section, it is worth highlighting that the addition of photogenerated radicals onto olefins different from Michael acceptors (e.g., electron-rich C=C bonds or captodative olefins) intended for the formation of C(sp^3^)–C(sp^3^) bonds has only a few precedents in the literature. These processes, however, took place only in the presence of high PC_HAT_ loadings or showed a low conversion of the starting materials.^157,200,325^

**Scheme 27:**
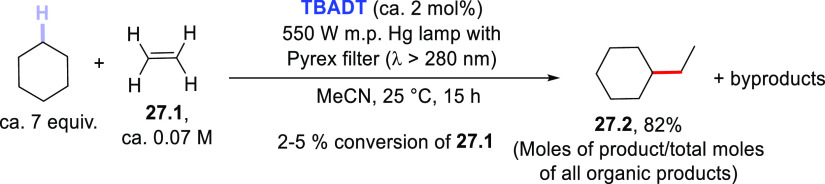
Photocatalyzed Derivatization of Electron-Rich Olefins

A representative example is shown in Scheme 27 where ethylene **27.1** was alkylated under **TBADT** photocatalysis to give **27.2** as the major product.^325^ Such examples have not been presented in detail here due to their limited synthetic significance.

### 2.2 Formation of C(sp^3^)–C(sp^3^) Bonds via Addition onto C=X (X = N, O) Bonds

**Scheme 28:**
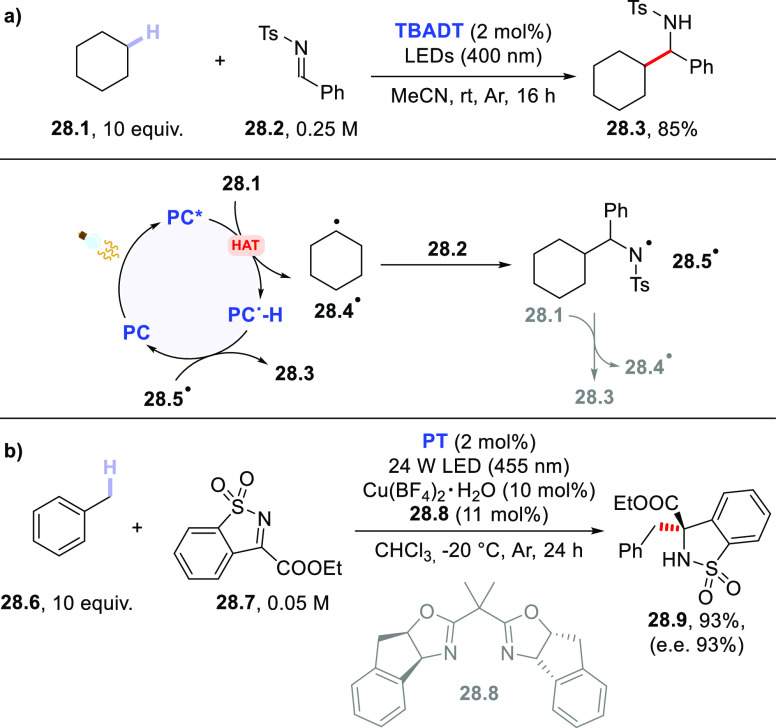
Photocatalyzed Addition of Hydrocarbons onto Imine Derivatives

The formation of C(sp^3^)–C(sp^3^) bonds can be realized also via the addition of a photogenerated radical onto a C=X (X = N, O) double bond. In order to promote reactivity, the *N*-atom typically bears an electron-withdrawing S-based substituent, either S(=O)_2_R or S(=O)R. Thus, *N*-tosylimines have been reported to act as excellent radical traps in **TBADT**-triggered alkylations with alkanes, ethers, and DMF. As reported in Scheme 28a, cyclohexane **28.1** (10 equiv) underwent addition onto the C=N bond of **28.2** to give the hydroalkylated adduct **28.3** in 85% yield in the presence of **TBADT** (2 mol %) upon irradiation with 400 nm LEDs (16 h). The occurrence of a chain mechanism (at least in part), however, could not be excluded.^407^ Similarly, chiral *N*-sulfinyl imines were smoothly alkylated by adamantane scaffolds in the presence of a catalytic amount of **PT** (5 mol %) upon irradiation with 390 nm LEDs. Notably, this strategy allowed the enantioselective synthesis of the saxagliptin core, containing an adamantyl-glycine motif.^279^ In another instance, a dual-catalytic system based on **PT** and a chiral Cu-based complex containing a bisoxazoline (BOX) ligand allowed the regio- and stereoselective functionalization of benzylic, allylic, and even unactivated hydrocarbons with an imine derivative. As shown in Scheme 28b, toluene **28.6** (10 equiv) reacted with **28.7** to give product **28.9** in an excellent yield (93%) and enantioselectivity (e.e. 93%) in the presence of **PT** (2 mol %) and Cu(BF_4_)_2_ (10 mol %) and chiral BOX ligand **28.8** (11 mol %).^281^

**Scheme 29:**
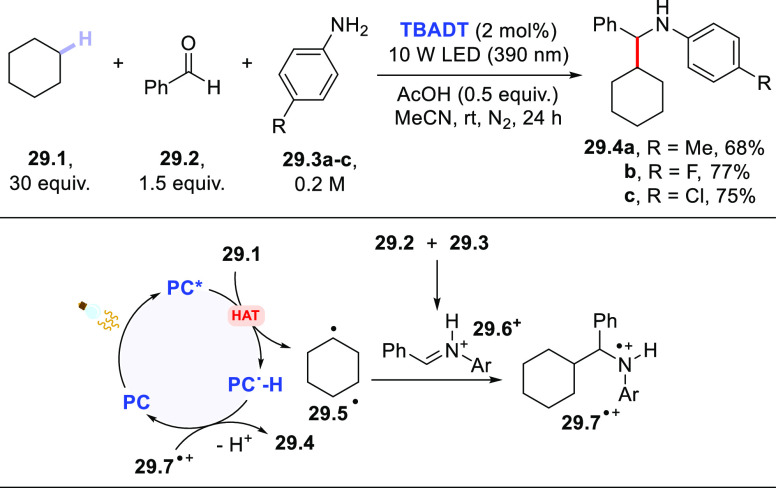
Multicomponent Synthesis of Secondary Amines

Very recently, the preparation of amines has been realized via a multicomponent carbonyl alkylative amination strategy. The protocol was promoted by **TBADT** (2 mol %) and comprised of *N*-arylamines, aldehydes, and hydrocarbons as starting materials. Slightly different conditions were required depending on the nature of the amine, being either an aniline or a diphenylamine. As shown in Scheme 29, the process involved the *in situ* formation of an iminium ion (**29.6**^**+**^), which acted as the trap of the photogenerated radical. When adopting cyclohexane **29.1**, benzaldehyde **29.2**, and anilines **29.3a–c**, secondary amines **29.4a–c** were obtained in good yields upon irradiation at 390 nm for 24 h, only requiring acetic acid (0.5 equiv) as an additive.^408^

**Scheme 30:**
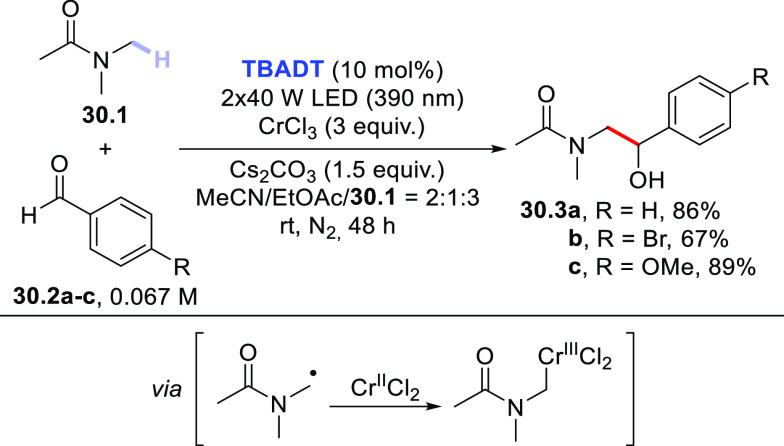
Photocatalyzed Functionalization of C=O Bonds

The challenging addition of photogenerated intermediates onto C=O bonds has been realized only in a few instances. One notable example involved a strategy comprised of **TBADT** and a Cr^III^ salt, where the role of the latter was to promote the formation of an organochromium compound via interception of the photogenerated radical. Indeed, this approach has been exploited to trigger the alkylation, aminomethylation, and oxymethylation of both aliphatic and aromatic aldehydes. Thus, *N*,*N*-dimethylacetamide **30.1** reacted with aldehydes **30.2a–c** to give 1,2-aminoalcohol derivatives **30.3a–c** in the presence of **TBADT** (10 mol %) and CrCl_3_ (3 equiv) upon irradiation with 390 nm LEDs for 48 h (Scheme 30).^409^

### 2.3 Formation of C(sp^3^)–C(sp^2^) Bonds

This section describes the formation of C(sp^3^)–C(sp^2^) bonds between a photocatalytically generated C(sp^3^)-centered radical and suitable reaction partners, which include alkynes via an addition process or vinyl/aryl derivatives via a (formal) substitution or cross-dehydrogenative coupling reaction. Likewise, the addition onto carbon monoxide (CO) or carbon dioxide (CO_2_) will be reported here.

**Scheme 31:**
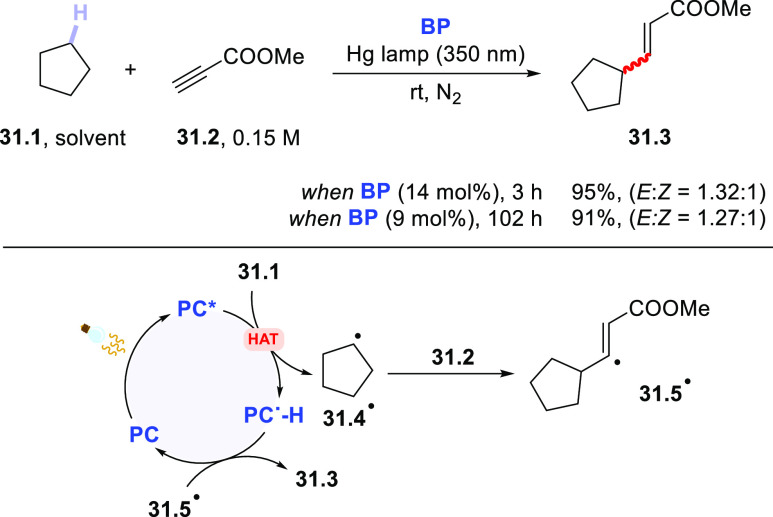
Photocatalyzed Addition of Cycloalkanes onto Alkynes

Seminal works in the field focused on the addition of cycloalkyl radicals, obtained from the corresponding hydrocarbons, onto electron-poor alkynes in the presence of aromatic carbonyls. Although these PCs_HAT_ were routinely adopted stoichiometrically, the reaction was demonstrated to work smoothly also in the presence of a catalytic amount of **BP**. Thus, methyl propiolate **31.2** was functionalized by cyclopentane (**31.1**, used as the solvent) to give an *E*/*Z* mixture of vinylcycloalkanes **31.3** (Scheme 31) in a very good yield. **BP** loading could be lowered to 9 mol % without affecting the reaction yield; albeit, a longer irradiation time was required in the latter case.^228^ Notably, the employed aromatic ketone could be supported onto a solid material (a polystyrene matrix or silica), rendering the PC_HAT_ potentially recyclable. This heterogeneous variant has been shown to work to some extent under natural sunlight irradiation.^410,411^ When applied to alcohols as substrates and dimethyl acetylenedicarboxylate as radical trap, this (heterogeneous) methodology opened the way to the generation of α-hydroxyalkyl radicals and to the preparation of γ-butenolides from them.^412^ Very recently, an analogous strategy based on the use of chloroalkynes (and, in selected cases, terminal alkynes) has been reported. **DCBP** (15 mol %) was used as the PC_HAT_, while the substrate scope included alcohols, ethers, amides, and even alkanes. Furthermore, when applied to THF, this process could be performed on the gram scale, without any significant yield decrease. Mechanistic studies revealed that this process occurred with a quantum yield >1, indicating the involvement of a radical chain mechanism.^413^ Similarly, the functionalization of chloroalkynes to give functionalized vinyl chlorides has been likewise carried out in the presence of **EY**(220) and **TBADT**,^277^ respectively.

**Scheme 32:**
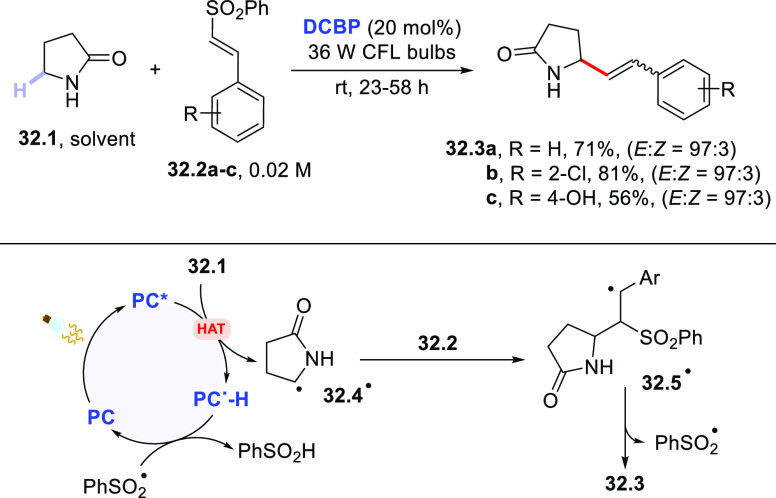
Photocatalyzed Alkenylation of Amides

Another option to forge a C(sp^3^)–C(sp^2^) bond is to intercept the photogenerated radical with an olefinic reaction partner containing a suitable radicofugal group. Thus, the alkenylation of ethers and amides with a library of vinyl sulfones smoothly occurred in the presence of **DCBP** (20 mol %) upon irradiation with CFL bulbs, wherein the loss of a sulfonyl radical occurred during the process. Thus, 2-pyrrolidone **32.1** (used as the solvent) reacted with sulfones **32.2a–c** to give the expected alkenylated amides **32.3a–c** in good yields and with a marked preference for the formation of the *E*-isomer (Scheme 32).^388^

**Scheme 33:**
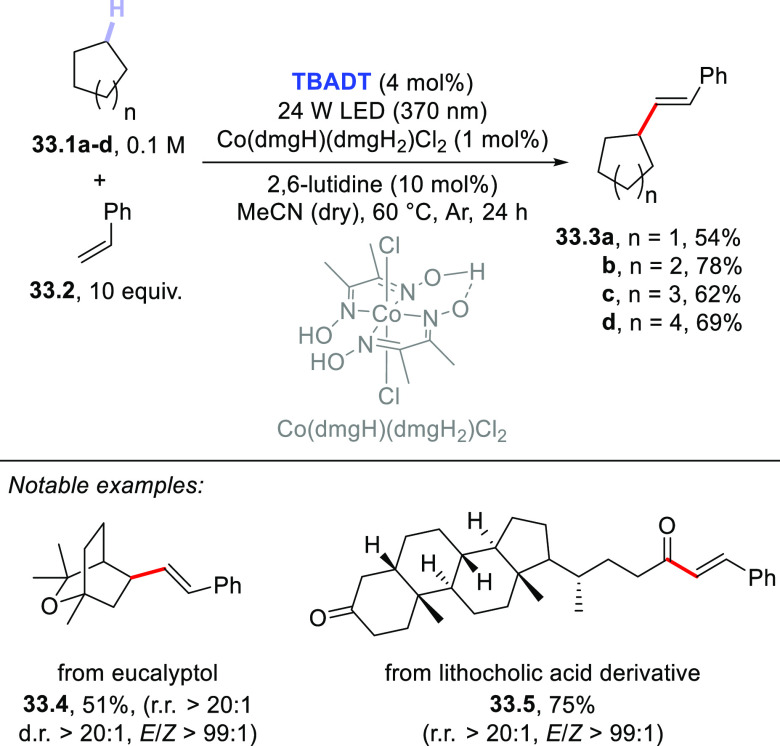
Dual-Catalyzed Dehydrogenative (*E*)-Alkenylation of Cycloalkanes

The dehydrogenative coupling between alkanes and aryl alkenes is also possible and has been recently realized thanks to a dual-catalytic strategy. This approach relies on the synergistic combination of **TBADT** photocatalysis with cobaloxime-mediated hydrogen-evolution cross-coupling. The Co-catalyst is responsible for intercepting the radical adduct formed upon addition of the photogenerated radical onto the olefin and then undergoes a photoinduced β-hydride elimination, restoring the original double bond. As depicted in Scheme 33, a series of cycloalkanes (**33.1a–d**) was alkenylated by styrene **33.2** (10 equiv) in the presence of **TBADT** (4 mol %), Co(dmgH)(dmgH_2_)Cl_2_ (1 mol %; dmgH_2_ and dmgH: dimethylglyoxime and its monoanion), and 2,6-lutidine (10 mol %) as the ligand to deliver adducts **33.3a–d** in good yields with complete regio- and stereoselectivity. The reaction took place in acetonitrile at 60 °C upon irradiation with a 370 nm LED and could be applied in the late-stage alkenylation of natural products, including steroid derivatives.^414^

**Scheme 34:**
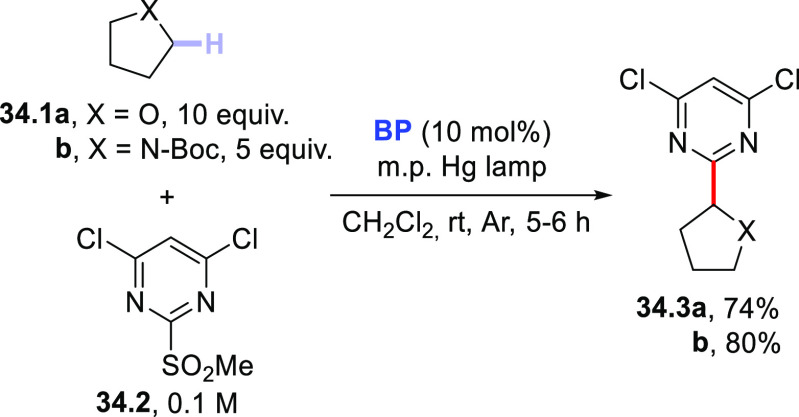
Photocatalyzed Arylation of Five-Membered Heterocycles

Turning to arylation reactions, the preparation of alkylated pyrimidines was realized through the coupling of saturated heterocycles (including oxygen-, nitrogen-, and sulfur-based derivatives) with sulfonylated pyrimidines in the presence of **BP** (10 mol %). As an example, 5-membered heterocycles **34.1a,b** were arylated by **34.2** to give pyrimidine derivatives **34.3a,b** in good yields upon irradiation with a medium-pressure Hg lamp via an *ipso*-substitution process (Scheme 34).^415^ Similarly, **EY** (2 mol %) was employed to promote the arylation of THF at the 2-position upon reaction with 2-phenylsulfonylbenzothiazole.^220^ In a related instance, the 4-pyridination of cumene at the benzylic position was performed in the presence of a catalytic amount of **BP** (10 mol %). In the process, the photogenerated radical added onto 4-cyanopyridine, while the desired product was formed upon loss of HCN from the initially formed adduct.^229^ In a very recent report, *N*-aminopyridinium salts have been likewise used as radical traps for photogenerated C-centered radicals (**AQ** as the photocatalyst) and enabled the site-selective C–H pyridylation of unactivated alkanes. Notably, this protocol could be adopted for the late-stage site-selective functionalization of biorelevant compounds.^416^

**Scheme 35:**
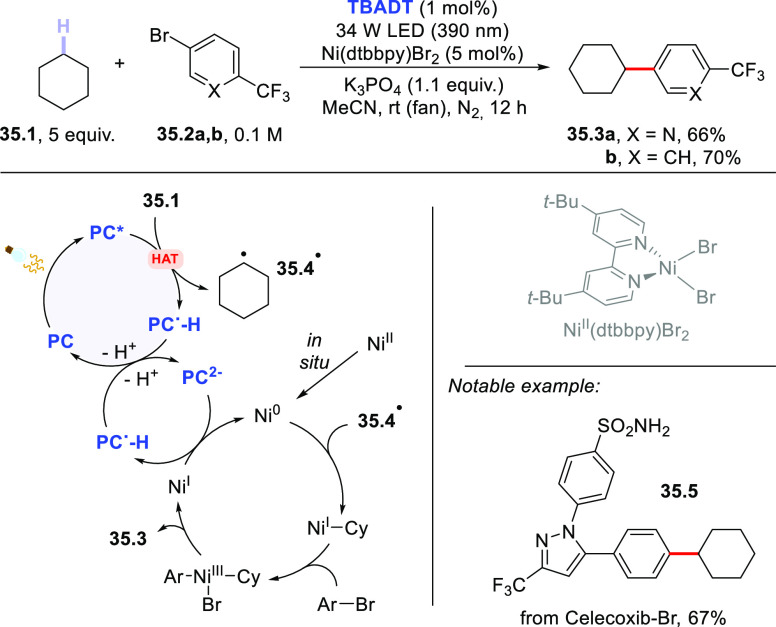
Arylation of Strong C–H Bonds via a TBADT/Nickel Dual-Catalyzed Strategy

The merging of HAT photocatalysis with Ni-catalysis opened new avenues on the route toward the arylation of (strong) aliphatic C–H bonds, allowing adoption of aromatic halides (mostly, bromides) as coupling partners. In particular, the Ni-based cocatalyst was responsible for activating the C(sp^2^)–Br bond and intercepting the photogenerated radical. This chemistry was successfully combined with different classes of PCs_HAT_, including **DT**(417) and aromatic carbonyls.^418,419^ Thus, cyclohexane **35.1** was functionalized by (hetero)aryl bromides **35.2a,b** to deliver cross-coupled products **35.3a–b** in very good yields upon irradiation with a 390 nm LED (Scheme 35). Of note, this protocol could be applied to the manipulation of natural products (see the case of **35.5**).^417^ Very recently, a dual-catalytic strategy based on **DCBP** and a Ni-based complex enabled the construction of C(sp^3^)–C(sp^2^) bonds via the acylation of methylbenzenes with *N*-acylsuccinimides.^420^

**Scheme 36:**
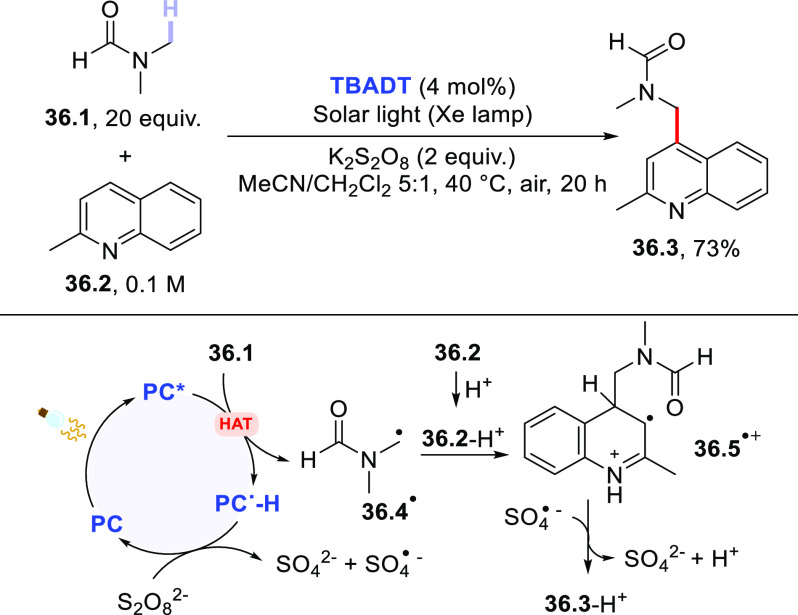
Photocatalyzed Cross-Dehydrogenative Coupling between Amides and Heteroarenes

Another opportunity for C(sp^3^)–C(sp^2^) bond construction is represented by photocatalytic cross-dehydrogenative couplings (CDC)^421^ between aliphatic H-donors and (hetero)arenes. These processes require the adoption of oxidative conditions to remove the extra electrons, and this has been realized either by having recourse to a chemical oxidant or through electrochemical means. As for the former case, **DT** photocatalysis has been successfully exploited to trigger the functionalization of alkanes, ethers, and amides with heteroarenes in the presence of a persulfate salt. As an example, this Minisci-type reaction allowed the functionalization of quinaldine (**36.2**) with DMF (**36.1**) to give **36.3** as the only product (73% isolated yield) in the presence of **TBADT** (4 mol %) and K_2_S_2_O_8_ (2 equiv) upon irradiation with simulated solar light (Scheme 36).^422^ More recently, a similar strategy has been applied to the preparation of 2-alkylated benzothiazoles under chemical oxidant-free photoelectrochemical conditions.^423^

**Scheme 37:**
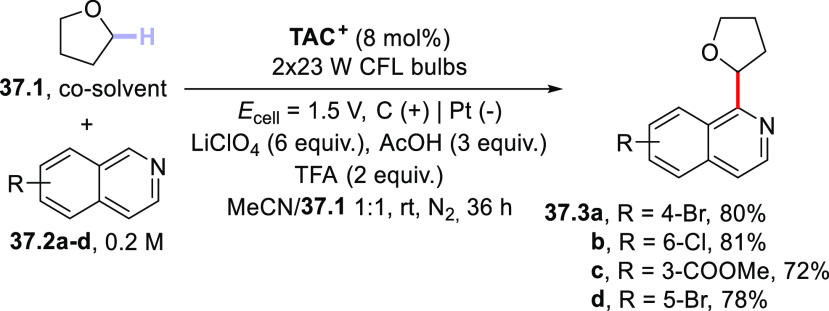
Electrophotocatalytic Arylation of Ethers Mediated by the Trisaminocyclopropenium (TAC^+^) Ion

A different cross-dehydrogenative coupling encompassing the merging between HAT photocatalysis and electrochemistry allowed the regioselective functionalization of ethers with isoquinolines. This strategy, tagged “electrophotocatalysis”, was based on the use of a trisaminocyclopropenium ion (TAC^+^), which was electrochemically converted to the stable **TAC**^**•2+**^ species via one-electron oxidation. The latter species then underwent excitation and, once in the excited state, triggered the desired HAT from the chosen ether. Thus, adducts **37.3a–d** have been prepared by reaction between THF (**37.1**) and substituted isoquinolines **37.2a–d** in the presence of TAC^+^ (perchlorate salt; 1 mol %) upon application of a constant potential (*E*_cell_ = 1.5 V) under irradiation with a CFL (Scheme 37).^209^

**Scheme 38:**
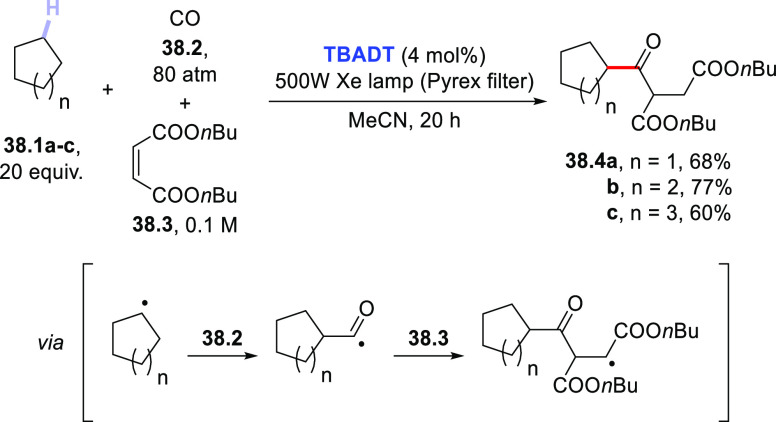
Three-Component Photocatalyzed Synthesis of Unsymmetrical Ketones

Carbon monoxide (CO) is an excellent radical trap and has been frequently exploited to get access to valuable acyl radicals.^424−426^ Seminal examples of this chemistry within photocatalytic applications date back to the early 90s, when aromatic carbonyls where adopted to promote the carbonylation of alkanes (mainly, cyclohexane) to afford cyclohexanecarboxaldehyde either under high CO pressure (20–80 atm)^427^ or in the presence of metal carbonyl complexes based on Ir, Rh, or Ru.^428^ Around the same period, an 8% formation of cyclohexanecarboxaldehyde from cyclohexane and CO (1 atm) in the presence of **TBADT** was reported.^326^ More recently, the functionalization of alkanes with electron-poor olefins under photocatalytic conditions mediated by **TBADT** was realized in the presence of CO. This allowed the preparation of unsymmetrical ketones in an atom-economical fashion in an overall multicomponent process, where the photogenerated alkyl radical was trapped by CO to form a C(sp^3^)–C(sp^2^) bond and then by the chosen electron-poor olefin. Thus, upon irradiation with a Xe lamp equipped with a Pyrex filter, 5- to 7-membered cycloalkanes **38.1a–c** reacted with dibutyl maleate **38.3** under an atmosphere of CO (**38.2**, 80 atm) in the presence of **TBADT** (4 mol %) to afford ketones **38.4a–c** in good isolated yields (Scheme 38).^429^ Later, the same protocol was applied to the regioselective β-acylation of cyclopentanone in the role of H-donor,^83^ as well as to the preparation of acyl hydrazides using diisopropyl azodicarboxylate (DIAD) in place of electron-poor olefins.^430^

In one instance, the carboxylation of the allylic position in simple alkenes by CO_2_ has been realized in the presence of 3,6-diphenylxanthone and a Cu-based complex. The process has been proposed to occur through a sequence involving two independent steps, where activation of the allylic C–H bond was promoted by the excited carbonyl, while the copper complex operated the desired carboxylation, also restoring the initial ketone. Indeed, it was demonstrated that both the xanthone derivative and the copper complex behaved catalytically in the overall process.^285^

### 2.4 Formation of C(sp^3^)–C(sp) Bonds

**Scheme 39:**
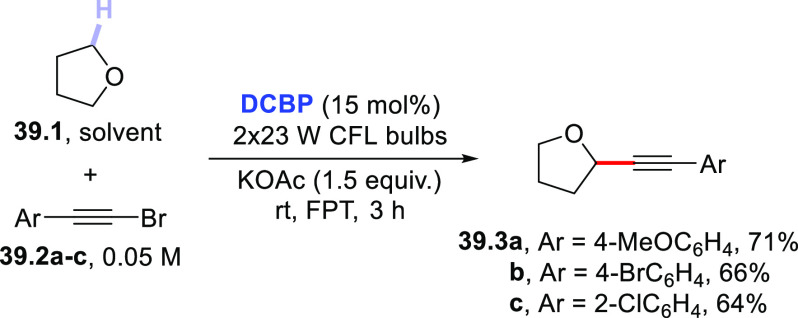
Photocatalyzed Alkynylation of Ethers by Bromoalkynes

The HAT-photocatalyzed C(sp^3^)–H to C(sp^3^)–C(sp) bond conversion can be related to two different families of processes, namely the introduction of an alkynyl or a cyano group. As for the first instance, the photogenerated radicals have been trapped by suitable alkynylating agents, namely alkynes substituted with a convenient radicofugal group. Indeed, only a handful of examples of this chemistry have been reported, which are based on the use of bromoalkynes,^413^ alkynylbenziodoxolones,^237^ or alkynylsulfones.^277,388^ Either the aromatic ketone **DCBP**(237,388,413) or **DT**(277) was used as the PCs_HAT_. As an example, THF (**39.1**, used as the solvent) reacted with bromoalkynes **39.2a–c** in the presence of **DCBP** (15 mol %) and KOAc (1.5 equiv) to give alkynes **39.3a–c** in good isolated yield, independently from the electronic character of the aromatic substituent in **39.2a–c** (Scheme 39). In the process, the formation of a vinyl bromide intermediate initially takes place, which then undergoes HBr elimination to give the desired alkynylated product aided by the employed base (KOAc).^413^

**Scheme 40:**
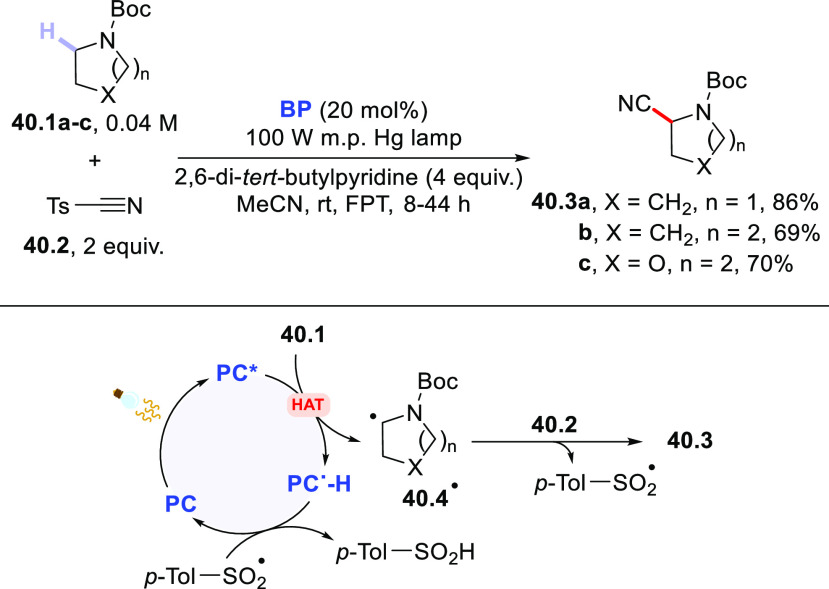
BP-Photocatalyzed Cyanation of Nitrogen-Containing Heterocycles

Turning to cyanation processes, an excellent option to intercept the photogenerated radical is using tosyl cyanide, which allows introduction of the desired cyano group via displacement of the sulfonyl moiety. Thus, **BP** can be successfully adopted to trigger this transformation in a variety of substrates, including ethers, alkanes, and nitrogen-containing substrates; however, only in the latter case it behaves as a real PC_HAT_. Thus, protected nitrogen-heterocycles **40.1a–c** were cyanated by tosyl cyanide **40.2** (2 equiv) to give **40.3a–c** in good to excellent yield in the presence of **BP** (20 mol %) and 2,6-di-*tert*-butylpyridine (4 equiv; functioning as an acid scavenger) upon irradiation with a medium-pressure Hg lamp (Scheme 40). Worthy of notice is the example related to **40.1c**, wherein the functionalization of the α-to-N position occurred chemoselectively.^230^ Similarly, **EY** (2 mol %) has been adopted in the C–H to C–CN conversion in 1,4-dioxane in the presence of tosyl cyanide.^220^ A different strategy is based on the use of a seven-coordinated (chiral) **Ur** salen complex (2 mol %), which was used for the cyanation of a variety of (substituted) *N*,*N*-dimethylanilines under oxidative conditions (H_2_O_2_) in the presence of NaCN and AcOH.^431^

### 2.5 Formation of C(sp^2^)–C(sp^3^) Bonds

**Scheme 41:**
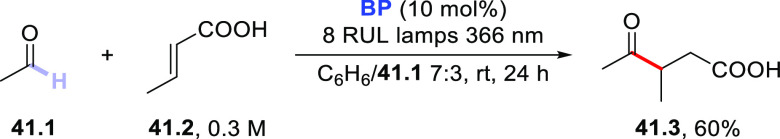
Photocatalyzed Acetylation of Crotonic Acid

This section gathers examples enabling the formation of a C(sp^2^)–C(sp^3^) bond via the intermediacy of a photogenerated C(sp^2^)-hybridized radical. Specifically, either aldehydes or formamides (see also section 2.1.2) can be exploited as H-donors in the formation of acyl and carbamoyl radicals, respectively. Seminal works in the area involved the use of **BP** to trigger the acylation of enones (mainly carbohydrate enones, see also Scheme 7 for the analogous C(sp^3^)–C(sp^3^) bond formation)^233^ and α,β-unsaturated esters or acids^234^ with aldehydes. An interesting example is reported in Scheme 41, showing the hydroacylation of crotonic acid **41.2** with acetaldehyde **41.1** to give 4-oxoalkanoic acid **41.3** (60% yield) in the presence of **BP** (10 mol %) upon irradiation at 366 nm for 24 h.^234^

**Scheme 42:**
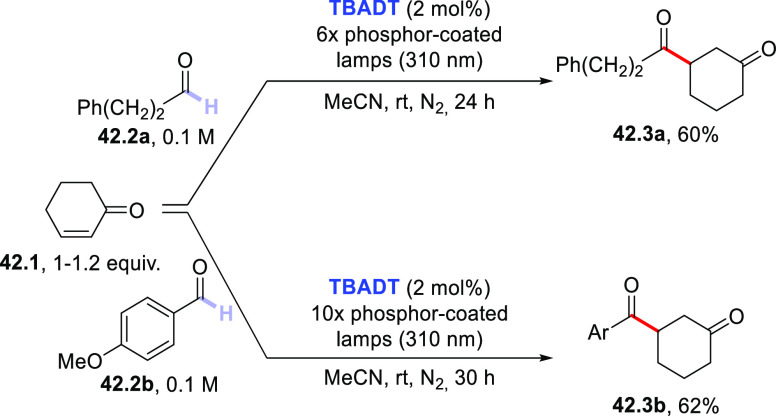
Cyclohexenone Acylation by Addition of Aliphatic (Upper Part) and Aromatic (Lower Part) Aldehydes

More recently, given their excellent reactivity as H-donors, aldehydes have been adopted as substrates in combination with a plethora of PCs_HAT_ to perform the hydroacylation of a huge variety of unsaturated systems. One of the most studied systems involves the functionalization of electron-poor olefins (α,β-unsaturated esters, ketones, and nitriles, as well as vinyl sulfones) triggered by **TBADT**.^382,432,433^ As an example, 2-cyclohexenone **42.1** was smoothly acylated by both hydrocinnamaldehyde **42.2a** and *p*-anisaldehyde **42.2b** to give interesting 1,4-diketones **42.3a,b** in a good yield (Scheme 42). Thus, the optimal **TBADT** loading was 2 mol %, but contrary to aliphatic aldehydes, a slight excess of **42.1** (1.2 equiv), a longer irradiation time (30 vs 24 h), and an increased light intensity were required in the preparation of **42.3b.**(432,433) Of note, these reactions have been demonstrated to occur under natural sunlight irradiation by simply exposing the reaction vessel containing the mixture on a window ledge for a few days. In the acylation of dimethyl maleate with heptanal, it was possible to increase the concentration of the starting materials up to 0.5 M, therefore reducing the amount of solvent needed and bringing about important ecological advantages.^276^

The environmental performance of these acylations was further ameliorated making use of continuous flow conditions.^381^ The adoption of this operation mode also allowed design of multistep procedures, wherein the photocatalytic C(sp^2^)–C(sp^3^) bond formation was followed by additional thermal steps on the resulting acylated derivatives.^434,435^

**Scheme 43:**
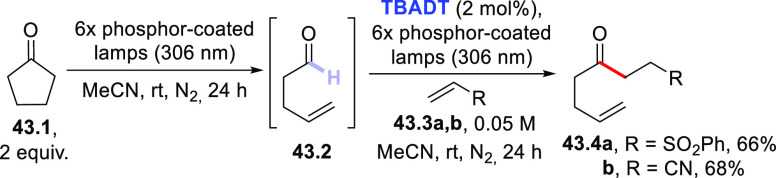
Preparation of Homoallyl Ketones from Cyclopentanones via a Two-Step Photochemical Norrish Type-I Cleavage/Photocatalyzed Hydroacylation Sequence

An elegant one-pot protocol comprised of two distinct photochemical steps was adopted for the preparation of homoallyl ketones starting from cyclopentanones and electron-poor olefins. The sequence involved an initial Norrish type-I photoinduced fragmentation of the 5-membered ring to give a 4-pentenal derivative followed by the **TBADT**-photocatalyzed hydroacylation of an electron-poor olefin. Thus, cyclopentanone **43.1** underwent ring opening via photoinduced cleavage of the C_1_–C_2_ bond to give aldehyde **43.2**. Next, **TBADT** (4 mol %) and the chosen electron-poor olefins (e.g., **43.3a,b**) were added to the crude mixture, finally affording the desired adducts (**43.4a,b**) in a good yield upon irradiation for an additional 24 h (Scheme 43).^436^

**Scheme 44:**
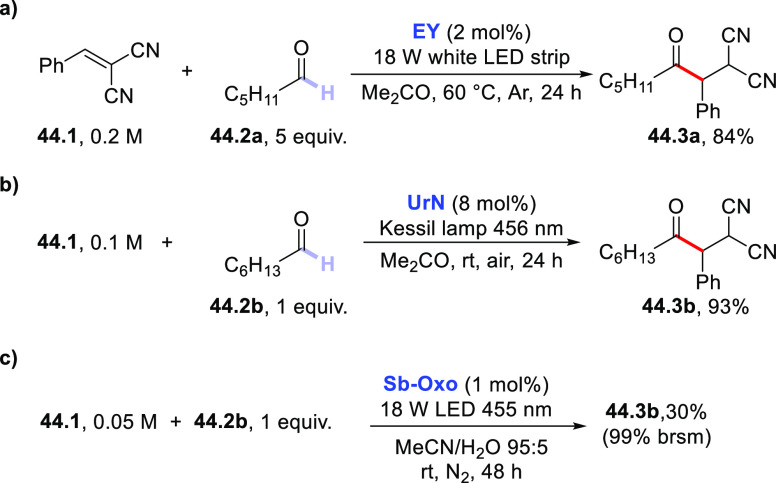
Hydroacylation of Benzylidene Malononitrile Triggered by Different PCs_HAT_

Apart from **TBADT**, other PCs_HAT_ have been recently reported to promote the hydroacylation of electron-poor olefins under visible light irradiation, including **EY**,^220^**UrN**,^283^ and **Sb-Oxo.**(265)Scheme 44 gathers selected examples describing the hydroacylation of benzylidene malononitrile **44.1**. Thus, **EY** and **UrN** allowed the hydroacylation of **44.1** with hexanal **44.2a** and heptanal **44.2b** to give the corresponding adducts **44.3a** and **44.3b** in 84 and 93% isolated yield, respectively (Schemes 44a,b).^220,283^ On the other hand, the preparation of **44.3b** in the presence of **Sb-Oxo** proceeded with a partial conversion of the starting materials; however, an almost quantitative yield based on remaining starting material (99% brsm) was observed (Scheme 44c).^265^

**EY** has been further tested for the asymmetric synthesis of 1,4-dicarbonyls. This strategy encompassed a dual-catalytic system, which also involved the use of a chiral rhodium catalyst responsible for coordinating the chosen electron-poor olefin (an unsaturated *N*-acylpyrazole) and driving the radical addition step (for a related example, see Scheme 19d).^390^

**Scheme 45:**
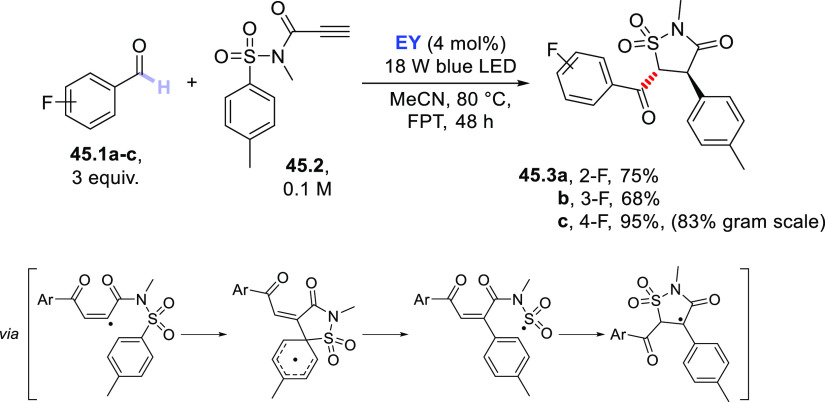
Photocatalyzed Preparation of Isothiazolidin-3-one 1,1-Dioxides

Very recently, the formation of a C(sp^2^)–C(sp^3^) bond by the **EY**-photocatalyzed reaction of aldehydes with *N*-(hetero)arylsulfonyl propiolamides has been proposed. This transformation led to the preparation of the isothiazolidin-3-one 1,1-dioxide core and proceeded through a cascade involving addition of the photogenerated radical onto the C≡C triple bond of propiolamide, Smiles rearrangement, and 5-*endo*-trig cyclization.^437^ As an example, *N*-heterocycles **45.3a–c** have been prepared from aldehydes **45.1a–c** and amide **45.2** upon irradiation with blue light for 48 h in the presence of **EY** (4 mol %, Scheme 45). The preparation of **45.3c** has been successfully realized on a gram scale in 83% yield, and of note, some of the synthesized compounds may have potential anticancer activity.^437^

**Scheme 46:**
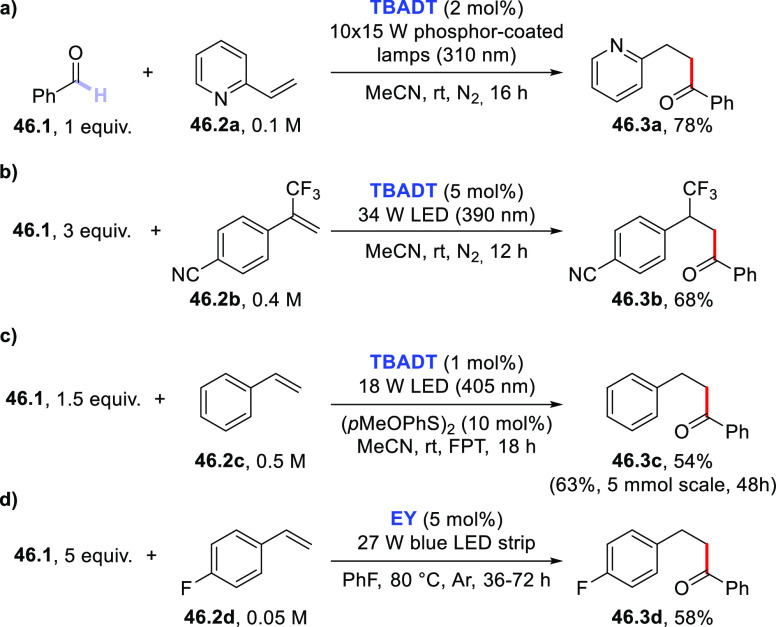
Photocatalyzed Hydroacylation of Vinyl (Hetero)aromatics

Apart from the use of electron-poor olefins, (hetero)aromatic alkenes can be used as well as radical traps for the photogenerated acyl radicals, with the driving force for the process being the formation of a stabilized benzyl radical. Thus, different combinations of the PC_HAT_ and alkenes have been adopted, including vinylpyridines,^386^ α-trifluoromethyl aryl alkenes (in this case, an aliphatic trifluoromethylalkene has been used as well),^438^ and aryl alkenes.^378,439^Scheme 46 gathers the case of benzaldehyde **46.1**, that has been adopted for the functionalization of 2-vinylpyridine **46.2a**, CF_3_-substituted alkene **46.2b**, styrene **46.2c** (in all cases **TBADT** as the PC_HAT_), and *p*-fluorostyrene **46.2d** (**EY** as the PC_HAT_).

Finally, *N*-tosyl imines (for a related example, see Scheme 28a)^407^ and dehydroalanine derivatives^440^ have been likewise adopted as acyl radical traps under **TBADT**-mediated photocatalytic conditions.

**Scheme 47:**
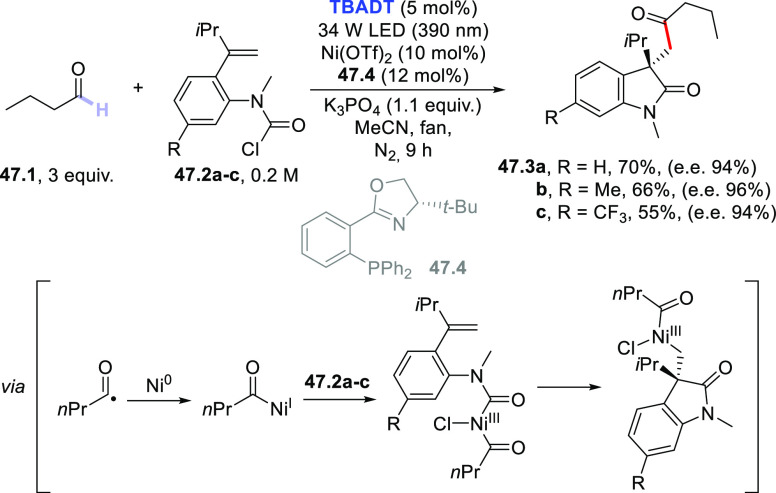
Asymmetric Acyl-Carbamoylation of Alkenes

Along the same line, a dual-catalytic strategy comprised of **TBADT** and a Ni-based cocatalyst allowed the asymmetric acyl-carbamoylation of alkenes starting from aldehydes and a carbamoyl chloride incorporating a C=C double bond. In this case, the Ni-based cocatalyst intercepted the photogenerated acyl radical and triggered the activation of the carbamoyl chloride, supervising the sequence of steps leading to the formation of the final product. Thus, butanal **47.1** reacted with aryl carbamic chlorides **47.2a–c** in the presence of **TBADT** (5 mol %), Ni(OTf)_2_ (10 mol %), ligand **47.4** (12 mol %), and K_3_PO_4_ (1.1 equiv). The process took place in MeCN upon irradiation with a 390 nm LED for 9 h, delivering oxindoles **47.3a–c** in good yields and excellent enantioselectivity (Scheme 47).^441^

**Scheme 48:**
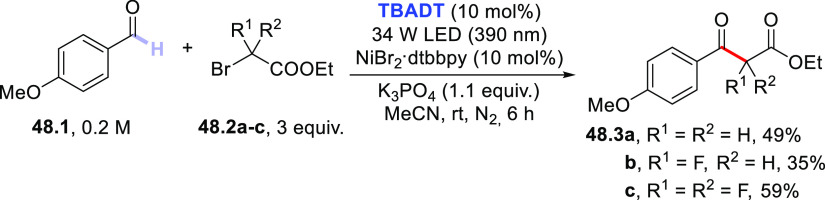
Synthesis of 1,3-Dicarbonyl Derivatives via a Dual-Catalytic Strategy

A similar reaction system, comprised of **TBADT** and a Ni-based cocatalyst, enabled the cross-coupling between acyl radicals, photogenerated from aldehydes, and (fluorinated) α-bromoacetates. As reported in Scheme 48, 1,3-dicarbonyl derivatives **48.3a–c** were readily accessed upon reaction between *p*-anisaldehyde **48.1** and esters **48.2a–c**.^442^

As mentioned above, apart from aldehydes, formamides can be likewise adopted as H-donors for the generation of carbamoyl radicals. These, in turn, are interesting intermediates for C(sp^2^)–C(sp^3^) bond formation campaigns.^157^ Thus, only a handful of PCs_HAT_ have been employed to trigger this reactivity, including **TBADT**(381,382,385,386,389) and **UrN**.^283^ In the latter case, however, this chemistry represented a minor pathway with respect to the preferred α-to-N C–H cleavage (see also section 2.1.2).^283^

**Scheme 49:**
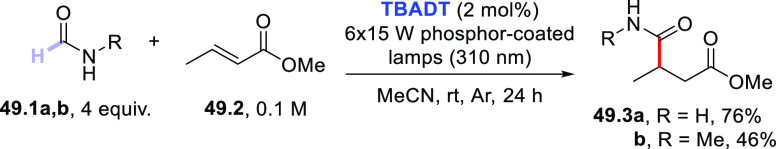
TBADT-Photocatalyzed Carbamoylation of Electron-Poor Olefins

On one hand, **TBADT** has been shown to cleave chemoselectively the C(=O)–H bond in primary and secondary formamides, while a completely different reactivity has been observed with tertiary formamides, (e.g., DMF, see Scheme 19b).^385^ As shown in Scheme 49, methyl crotonate **49.2** has been successfully carbamoylated by formamide **49.1a** and *N*-methyl formamide **49.1b** in the presence of **TBADT** (2 mol %) upon irradiation with phosphor-coated lamps centered at 310 nm. Indeed, the former H-donor led to the formation of product **49.3a** in a higher yield (76%) than the latter (**49.3b**, 46% yield). The carbamoylation of electron-poor olefins has been successfully implemented under continuous flow conditions, delivering the desired products in shorter reaction times and increased productivity.^381,389^ More recently, the same reactivity has been likewise applied to the carbamoylation of vinylpyridines^386^ and styrenes^378^ (in the latter case, in the presence of a disulfide cocatalyst). In sharp contrast, **UrN** enabled the functionalization of the C(=O)–H bond even when using DMF as substrate, being a competitive path to the usual α-to-N functionalization.^283^

### 2.6 Formation of C(sp^2^)–C(sp^2^) Bonds

As in the previous section, the formation of C(sp^2^)–C(sp^2^) bonds made use of aldehydes or formamides as radical precursors. The photogenerated radicals, however, are here used in the addition onto C≡C triple bonds or in (formal) substitution reactions or cross-dehydrogenative couplings with substituted arenes or alkenes.

**Scheme 50:**
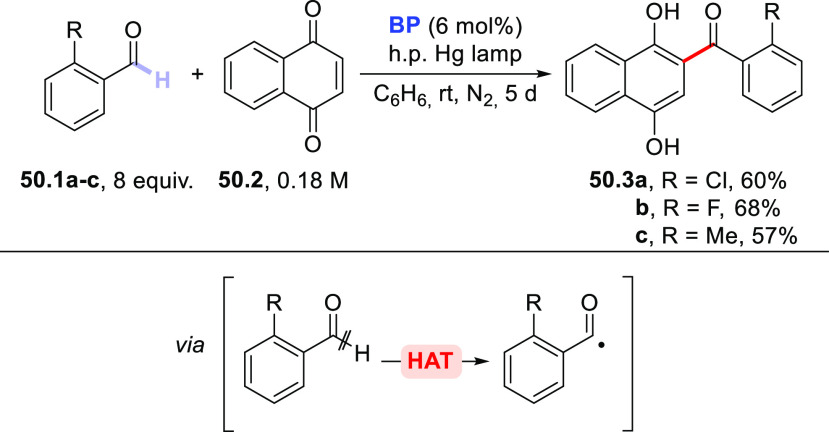
Photocatalyzed Acylation of 1,4-Naphthoquinone

Early examples described the acylation of quinones in the presence of **BP**,^231,232^ in what has been tagged as “photo-Friedel–Crafts acylation”.^232^ Albeit the reaction proceeded to some extent also in the absence of **BP**, its addition in catalytic quantities allowed an improvement of the product yield. As depicted in Scheme 50, 1,4-naphthoquinone **50.2** reacted with *ortho*-substituted benzaldehydes **50.1a–c** to afford hydroquinones **50.3a–c** in a good yield upon irradiation with a high-pressure mercury lamp for 5 days in the presence of **BP** (6 mol %).^231^

**Scheme 51:**
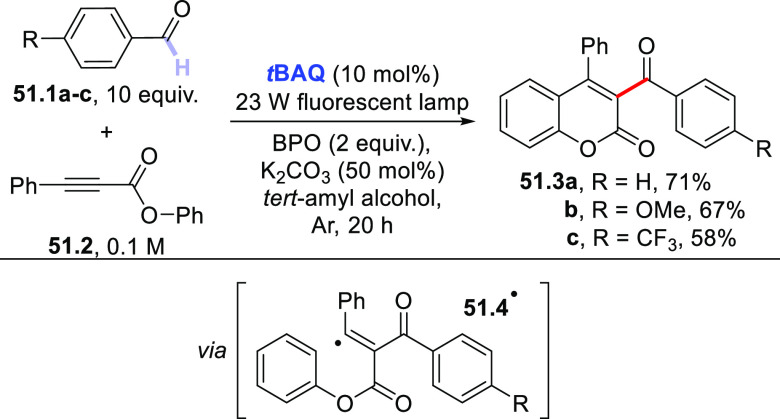
Photocatalyzed Acylation of Ynoates on the Route to Coumarin Scaffolds

More recently, the construction of the 3-acyl-4-arylcoumarin scaffold has been realized upon photocatalyzed addition of aldehydes (e.g., **51.1a–c**) onto an aromatic ynoate (**51.2**) triggered by an anthraquinone derivative (Scheme 51). Thus, the photogenerated acyl radical added regioselectively onto the α-position of the ynoate to give the vinyl radical **51.4**^**•**^, which cyclized onto the tethered aromatic ring followed by rearomatization to give coumarins **51.3a–c** in good yields. The reaction was promoted under visible light irradiation by adopting *t***BAQ** (10 mol %) and benzoyl peroxide (BPO) as a stoichiometric oxidant. A possible role of the carboxyl radical resulting from BPO decomposition in the desired C–H cleavage has been proposed. Of note, the biological activity of some of the synthesized coumarins has been studied, suggesting their efficacy as potential candidates for new therapeutics.^443^

**Scheme 52:**
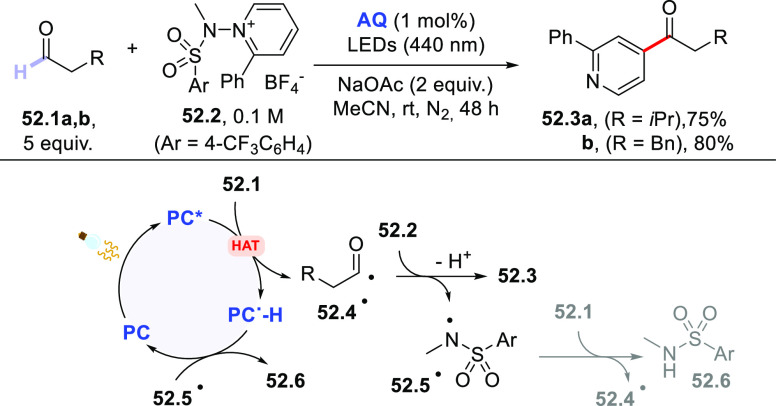
Photocatalyzed Preparation of Pyridyl Ketones

Very recently, **AQ** (1 mol %) has been used as the PC_HAT_ to promote the C(=O)–H pyridylation of a small library of aldehydes and formamide with *N*-aminopyridinium salts. As an example, Scheme 52 depicts the reaction between aldehydes **52.1a,b** and **52.2**, which took place upon visible light irradiation for 48 h, to deliver acylated pyridines **52.3a,b**. In the process, **AQ** triggered the formation of acyl radicals **52.4**^**•**^, which underwent addition onto the pyridinium derivative in a regioselective fashion and ultimately gave the desired products upon deprotonation and release of sulfonamidyl radical **52.5**^**•**^ via N–N bond cleavage. Indeed, the possible involvement of sulfonamidyl radical **52.5**^**•**^ in a chain pathway has been likewise proposed.^416^

**Scheme 53:**
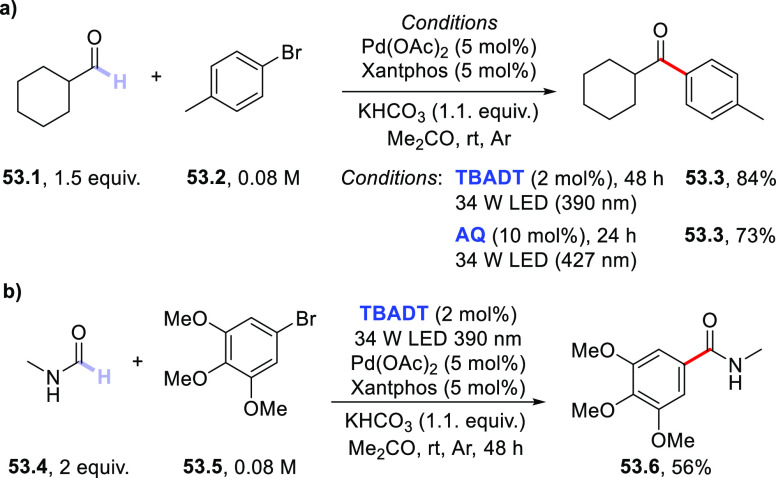
Photocatalyzed Preparation of Aromatic Ketones and Benzamides

Another opportunity for C(sp^2^)–C(sp^2^) bond formation is the cross-coupling between aldehydes and aromatics. Most of the reported routes rely on the adoption of a dual-catalytic strategy comprised of a PC_HAT_ (**TBADT**^227,442^ or an aromatic ketone^227^) and a transition-metal cocatalyst (based on Ni^441^ or Pd^227^), responsible for intercepting the photogenerated acyl radical and activating the aromatic derivative. Scheme 53a describes the arylation of cyclohexanecarboxaldehyde **53.1** with 4-bromotoluene **53.2**. Interestingly, two different PCs_HAT_ have been used in the preparation of **53.3**, either **TBADT** (2 mol %) or **AQ** (10 mol %), in the presence of the same cocatalyst, viz. Pd(OAc)_2_ (5 mol %) complexed with the Xantphos ligand (5 mol %). Notably, while the former system required UV light irradiation (390 nm), the latter worked upon visible light exposure (427 nm). Apart from aryl bromides, aryl iodides and triflates have been demonstrated to be competent substrates as well. One example also demonstrated that *N*-methyl formamide **53.4** could be used instead of aldehydes, enabling the carbamoylation of aryl bromide **53.5** to give benzamide **53.6** (Scheme 53b).^227^ Very recently, a dual-catalytic approach based on **PQ** and a Pd-based cocatalyst enabled the acylation of arenes via a double C–H activation strategy. The process took place under visible light irradiation in the presence of Ag_2_O as the terminal oxidant.^444^ In another instance, the net-oxidative cross-coupling between heptanal and quinaldine in the presence of **TBADT** (4 mol %) and K_2_S_2_O_8_ (2 equiv) has been described (see Scheme 36 for a related process).^422^ Also in this case, the aldehyde could be substituted with *N*-methyl formamide as the H-donor to unlock quinaldine carbamoylation.^422^

**Scheme 54:**
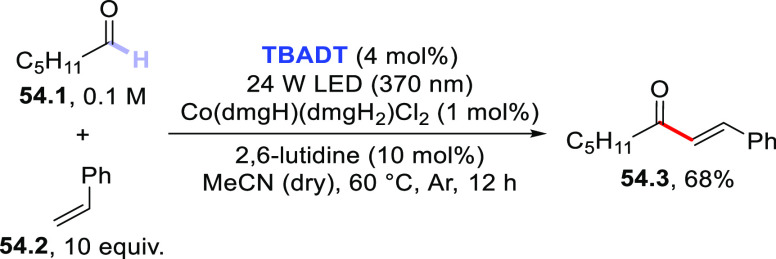
Dual-Catalytic Cross-Dehydrogenative Coupling between Aldehydes and Aryl Alkenes

Apart from aromatic derivatives, the formation of C(sp^2^)–C(sp^2^) bonds starting from aldehydes and alkenes has been reported. Only a couple of examples of this chemistry are available in the literature, both making use of dual-catalytic strategies. The first one enabled the cross-dehydrogenative coupling between aldehydes and alkenes, based on the use of **TBADT** and a Co-based cocatalyst. Thus, adopting the same protocol described in Scheme 33, hexanal **54.1** reacted with styrene **54.2** to give the α,β-unsaturated ketone **54.3** in a good yield (Scheme 54). Notably, the process occurred in a stereoselective fashion, affording exclusively the *E*-isomer.^414^ In one instance, the alkenylation of cyclohexanecarboxaldehyde with β-bromostyrene has been likewise realized through the merging of **TBADT** photocatalysis with Pd-catalysis (see also Scheme 53).^227^

### 2.7 Formation of C(sp^2^)–C(sp) Bonds

**Scheme 55:**
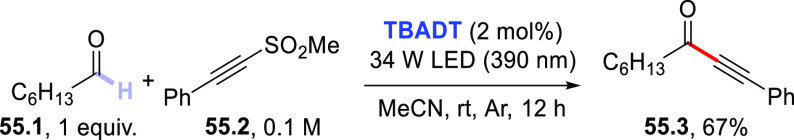
TBADT-Photocatalyzed Alkynylation of Aldehydes

Very recently, an example of C(sp^2^)–C(sp) bond formation has been reported, making use of a SOMOphilic alkynylation protocol. As shown in Scheme 55, heptanal **55.1** reacted with alkynyl sulfone **55.2** to give ynone **55.3** in a good isolated yield (67%) upon irradiation with a 390 nm LED in the presence of **TBADT** (2 mol %).^277^

### 2.8 Functionalization of Carbon Nanostructures

**Scheme 56:**
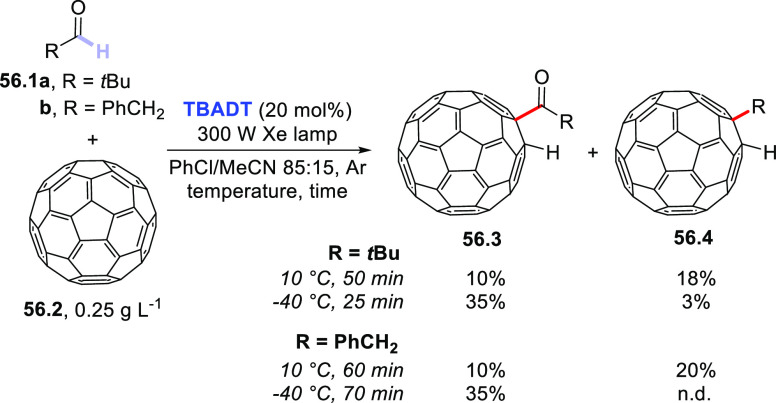
Functionalization of Fullerene-C60 via Addition of Photogenerated Acyl Radicals

Fullerene is one of the most investigated nanomaterials nowadays, and this is mainly due to its versatility in supramolecular chemistry,^445,446^ in technological applications,^447,448^ as well as in medicinal chemistry and nanotechnology.^449^ One of the most convenient ways to edit the fullerene structure, taking advantage of the high number of unsaturations, is by radical addition, wherein fullerene works as a radical sponge.^450^ However, the challenging aspect of this chemistry stems from the tendency of C_60_ to give a complex mixture of multiadducts. This reactivity could be tamed by having recourse to **DT**-photocatalyzed HAT;^451−453^ albeit, only in one case the PC_HAT_ could be used in a 20 mol % loading. Therein, the functionalization of fullerene C_60_ with acyl radicals generated from aromatic, α,β unsaturated and aliphatic aldehydes was reported.^454^ The mechanism is based on a fast radical–radical anion coupling between the acyl radical and the fullerene radical anion generated concomitantly with the recovery of the PC_HAT_. Intriguingly, it was found that when a pivaloyl or phenylacetyl radical was generated (for both intermediates the decarbonylation rate is >10^5^ s^–1^), CO loss occurred already at 10 °C. Notably, the decarbonylation step could be suppressed and the corresponding acylated fullerenes were synthesized with great selectivity by decreasing the temperature down to −40 °C. In fact, when pivalaldehyde (**56.1a**) or phenylacetaldehyde (**56.1b**) was subjected to the optimized reaction conditions at 10 °C, a mixture of **56.3** and **56.4** was obtained in the ratio 1:1.8 or 1:2, respectively. However, by decreasing the temperature, the ratio was completely reversed to 12:1 for **56.1a**, while for **56.1b** the decarbonylation product was not even observed (Scheme 56).

In another instance, the **DT**-photocatalyzed PEGylation of carbon nanotubes was reported.^455,456^ In particular, when a solution of single-walled carbon nanotubes was irradiated in the presence of **TBADT** in a PEG400/MeCN solution, the grafting occurred smoothly in 48 h, albeit with modest efficiency (20 wt %, 1 PEG400 chain every 120 carbon atoms). Interestingly, it was shown that sunlight could be used as an inexpensive light source and afforded the PEGylated carbon nanotubes in a comparable yield.^455,456^

## 3 Formation of C–Y Bonds (Y ≠ C)

Besides the formation of C–C bonds, photocatalyzed HAT has been successfully employed to forge C–Y bonds. Specifically, this section gathers synthetic examples dealing with the formation of C–N, C–O, C–S, C–F, and C–Cl bonds. Finally, the adoption of *d*-HAT for the deuteration of organic molecules is presented.

### 3.1 Formation of C–N Bonds

Diisopropyl azodicarboxylate (DIAD) is one of the most widely adopted unsaturated compounds to forge C–N bonds via photocatalyzed HAT. Thus, its remarkable electrophilic character makes DIAD an excellent trap for nucleophilic radicals such as alkyl, acyl, α-oxyalkyl, and α,α-dioxyalkyl. Upon addition of these nucleophilic radicals onto the N=N double bond, the corresponding N-centered radical is generated, which is typically entrusted for the recovery of the spent PC_HAT_.

**Scheme 57:**
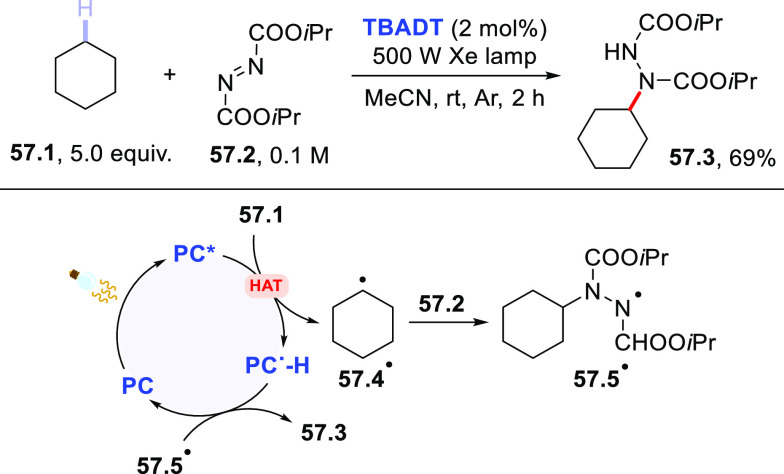
TBADT-Photocatalyzed Synthesis of Hydrazides

In one instance, DIAD was exploited for the synthesis of hydrazides, as shown in Scheme 57.^430^ Therein, **TBADT** was used for the functionalization of (cyclo)alkanes (e.g., cyclohexane **57.1**), ethers, and acetals via homolytic C–H bond cleavage. The photogenerated C-centered radicals were trapped by DIAD **57.2** to deliver the corresponding hydrazides (e.g., **57.3**) in good to excellent yields. Interestingly, aldehydes were competent substrates as well, allowing the synthesis of acyl hydrazides through the formation of a C(sp^2^)–N bond.^430^

**Scheme 58:**
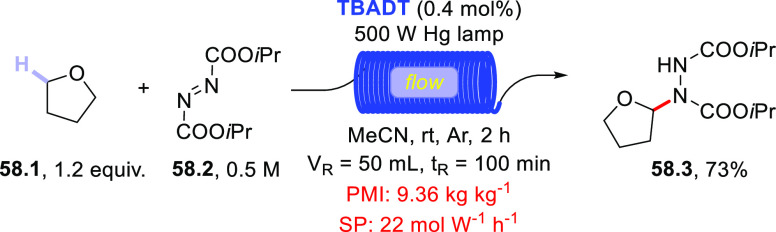
TBADT-Photocatalyzed Formation of a C–N Bond in Flow

The synthesis of hydrazides, including adduct **57.3**, was also reported under continuous-flow conditions. In such case, the photochemical reactor (V = 50 mL) consisted of a water-cooled 500 W medium-pressure Hg-vapor lamp wrapped in FEP (fluorinated ethylene propylene) tubing. When a MeCN solution of THF (**58.1**), DIAD, and **TBADT** (0.4 mol %) was flown through the tubing, the labile α-to-O C(sp^3^)–H bond of **58.1** was cleaved, thus delivering the corresponding α-oxyalkyl radical and, ultimately, product **58.3** in 73% yield upon reaction with **58.2** (Scheme 58). Interestingly, this reaction showed an excellent process mass intensity (PMI, i.e. the ratio of the total mass of materials to the mass of isolated product) of 9.36 kg kg^–^^1^ and a specific productivity (SP, i.e. mmol of product formed with respect to the energy consumed) of 22 mol W^–^^1^ h^–1^.^381^

**Scheme 59:**
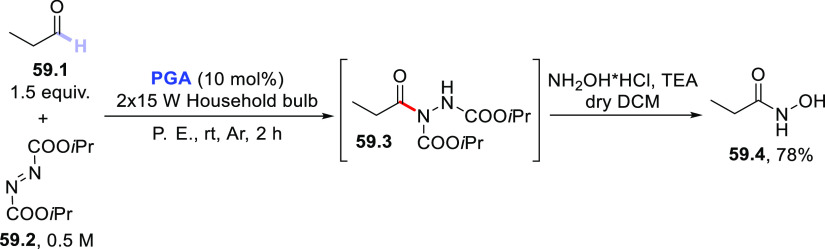
One-Pot Synthesis of Hydroxamic Acids from Aldehydes

A similar approach was reported for the one-pot synthesis of hydroxamic acids from aldehydes.^245^ In detail, acyl radicals were generated via HAT by **PGA** (10 mol %) and trapped by DIAD. The resulting acylhydrazides (e.g., **59.3**) were then converted in a one-pot fashion to the corresponding hydroxamic acids via nucleophilic acyl substitution with hydroxylamine. For example, propanal (**59.1**) was smoothly converted to hydroxamic acid **59.4** in 78% yield over two steps (Scheme 59).

This approach was also extended to the synthesis of amides by employing primary and secondary amines in the place of hydroxylamine.^457^ The validity and the robustness of the protocol were demonstrated through the preparation of Moclobemide (∼30% yield over two steps), a drug used against depression and social anxiety, starting from 4-chlorobenzaldehyde.^457^

**Scheme 60:**
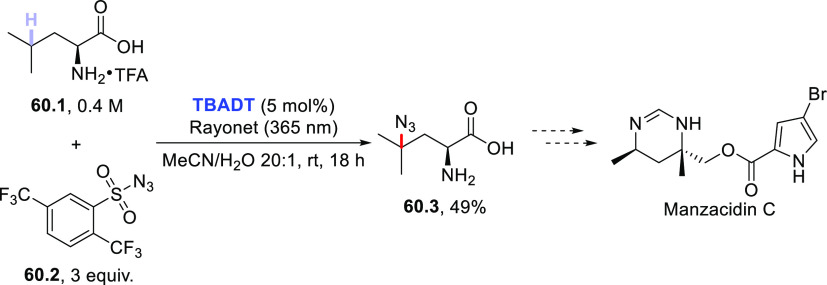
Azidation of the Methine Site of Leucine

A conceptually different route for the formation of C–N bonds consists in the synthesis of organic azides. This approach is particularly appealing since these compounds are widely used in medicinal chemistry for biorthogonal labeling via the Cu-catalyzed Huisgen reaction. In one instance, photocatalyzed HAT has been used to introduce the N_3_ group on leucine in the synthesis of Manzacidin C.^458,459^ The first step of the synthetic route consisted in the **TBADT**-promoted activation of the methine site of the amino acid (**60.1**); ensuing quenching of the photogenerated radical with sulfonyl azide **60.2** readily afforded the hoped-for azide **60.3** in 49% yield (Scheme 60).

**Scheme 61:**
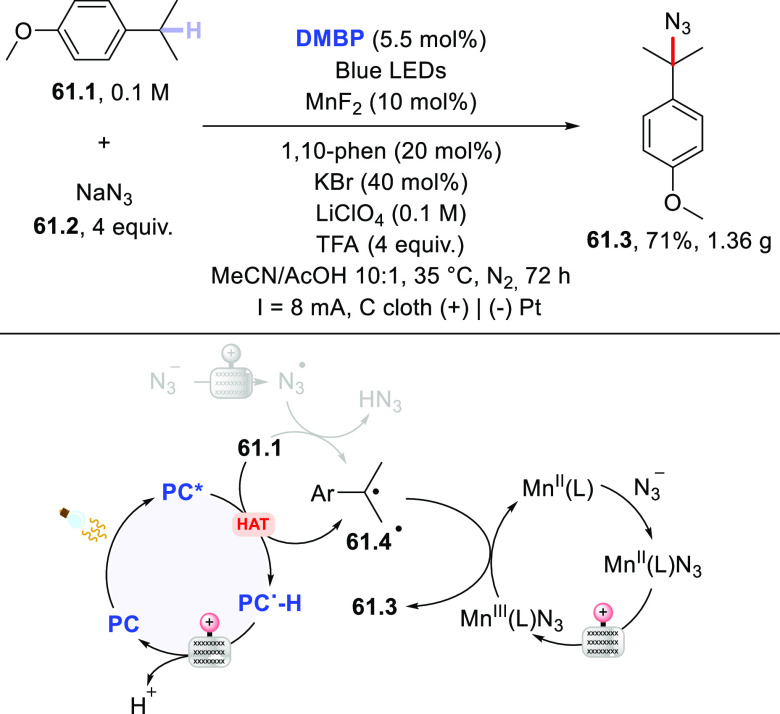
Electrophotocatalytic Azidation via a DMBP/Mn Dual-Catalytic System

Recently, the azidation of C(sp^3^)–H bonds via HAT has been achieved through an electrophotocatalytic strategy, where **DMBP** (5.5 mol %) was used as PC_HAT_ and a Mn-salt as the cocatalyst.^460^ In detail, compound **61.1** was activated via HAT to afford the corresponding (stabilized) benzyl radical. In the meanwhile, the manganese ion (from MnF_2_) was complexed by the added ligand (1,10-phenantroline) and the azide anion to afford a Mn^III^/L–N_3_ complex after anodic oxidation. The interaction between the benzyl radical and the latter species afforded the hoped-for organic azide and the Mn^II^ ion, prone to start a new catalytic cycle. It is however important to mention that the reaction proceeded to a certain extent (>40%) even without the photocatalyst, suggesting that a competitive direct anodic oxidation of the N_3_^–^ anion might be likewise responsible for the observed reactivity. Notably, the reaction could be run on a gram scale and product **61.3** was obtained in 71% yield (1.36 g, Scheme 61).

**Scheme 62:**
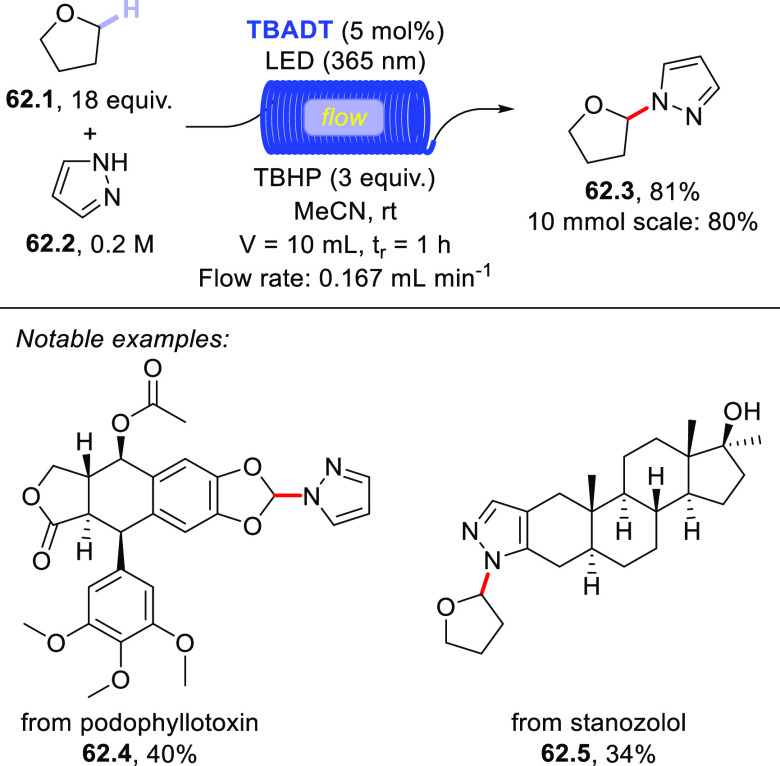
C(sp^3^)–H Heteroarylation via Radical-Polar Crossover

Very recently, a different concept for the C–H to C–N bond conversion based on radical-polar crossover (RPC) has been disclosed. RPC opens the world of polar chemistry to radicals and enables a whole new range of synthetic possibilities for radical chemistry.^461,462^ More specifically, HAT was coupled with a subsequent chemical oxidation of the first-formed C-centered radical to afford a carbocation. The latter intermediate could be conveniently trapped with *N*-heteroaryl-based nucleophiles, thus establishing the targeted C–N bond.^463^ Thus, when an acetonitrile solution of tetrahydrofuran (**62.1**, 6 equiv), pyrazole (**62.2**), **TBADT** (5 mol %), and TBHP (*tert-*butyl hydroperoxide, 3 equiv) was irradiated with a UV LED (365 nm) for 16 h, product **62.3** was formed in 86% ^1^H NMR yield. The reaction was made more efficient by adopting a flow apparatus, which allowed reduction of reaction time to just 1 h giving a similar result (86% ^1^H NMR yield, 81% isolated yield), even though 18 equiv of **62.1** was required (Scheme 62). Notably, the reaction remained efficient when performed on a 10 mmol scale (80% yield) and the synthetic approach could be employed for the late-stage functionalization of biologically active molecules such as acetylated podophyllotoxin (**62.4**) and stanozolol (**62.5**).^463^

### 3.2 Formation of C–O Bonds

The formation of C–O bonds is a widely investigated transformation among those that can be carried out via photocatalyzed HAT. In these manifolds, the C-centered radical generated via HAT is typically intercepted by a sacrificial oxidant, such as molecular oxygen (O_2_). The first-formed oxygenated intermediate is a hydroperoxyl radical, which can be isolated as the corresponding hydroperoxide or *in situ* converted to alcohols, ketones, and even carboxylic acids. Besides its operational simplicity, this transformation has remarkable commercial and industrial implications. For example, when using cyclohexane as the starting material, a mixture of cyclohexanol and cyclohexanone is obtained, the so-called KA–oil (ketone–alcohol mixture).^464^ The latter has an immense value in modern industry, since it is used for the synthesis of adipic acid, in turn needed to produce Nylon-6,6.^465^

In view of the above, several research groups have designed photocatalytic systems able to perform this transformation in a sustainable way and carried out meticulous mechanistic studies. Most of them rely on the use of metal-oxo compounds (e.g., the **DT** or **Ur** ions) or POCs (e.g., aromatic ketones) under aerobic conditions. Homogeneous conditions are routinely adopted;^201,314,472−476,333,338,466−476^ albeit, heterogeneous systems have been reported as well.^288,477,478^

Hereby, the synthetic applications of this chemistry have been reviewed and classified according to the aimed-for product, namely hydroperoxides, alcohols, and ketones or carboxylic acids.

#### 3.2.1 Synthesis of Hydroperoxides

**Scheme 63:**
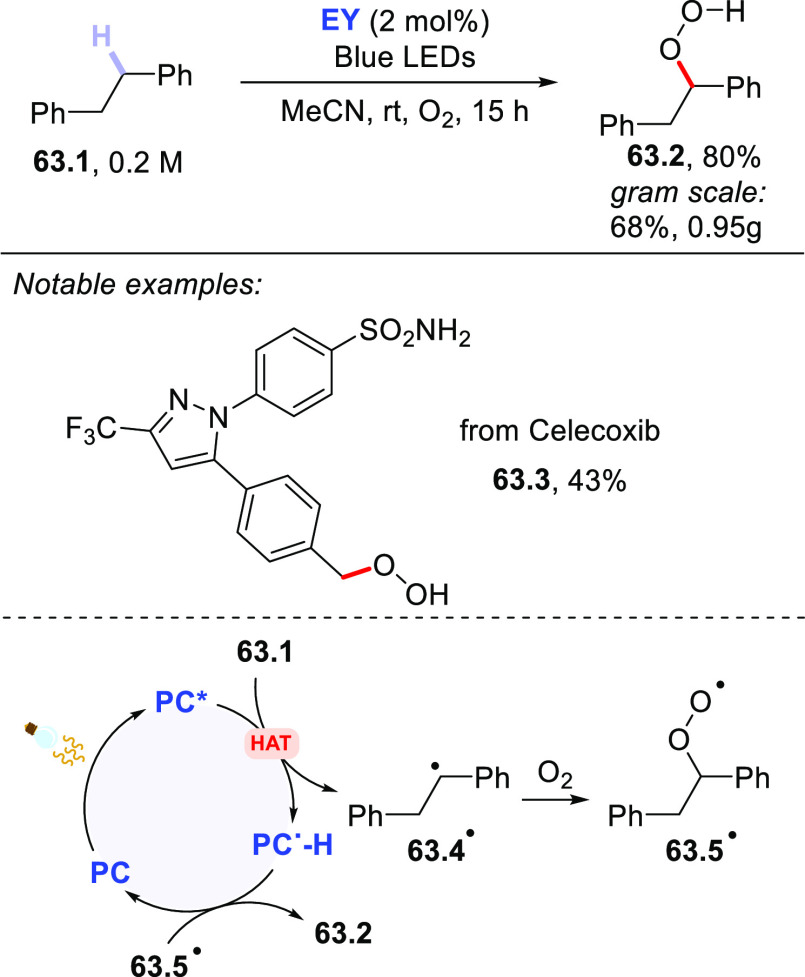
Photocatalyzed Benzylic Hydroperoxidation under Visible Light Irradiation

A seminal report on the synthesis of hydroperoxides via photocatalyzed HAT was published in the 1980s and focused on the oxidation of isobutane relying on the use of polyoxometalates as PCs_HAT_.^200,323^ In such a case, an acetonitrile solution of the light alkane was irradiated in the presence of **TBADT** (4 mol %) to afford *tert*-butylhydroperoxide in a quantitative yield (at 55% conversion of isobutane) after 2 h of irradiation with a 1000 W Xe lamp.^323^ In another instance, the hydroperoxidation of benzylic C–H bonds by using **EY** has been reported.^250^ The irradiation of an acetonitrile solution of **63.1** (0.2 M) under an O_2_-atmosphere with a blue LED in the presence of the organic dye (2 mol %) delivered compound **63.2** in 80% isolated yield (Scheme 63). Notably, the same reaction was run on a gram scale and 1.18 g of **63.1** was smoothly converted into 0.95 g of **63.2** (68% yield) after 15 h of irradiation.^250^

#### 3.2.2 Preparation of Alcohol and Ketone Mixtures

Under certain circumstances, hydroperoxyl radicals undergo spontaneous disproportionation to yield a mixture of the corresponding alcohol and ketone.^479^ This chemistry has been exploited in several instances, especially with cyclohexane as the substrate and **TBADT** as the PC_HAT_, to obtain a mixture of cyclohexanol and cyclohexanone.^322,480−487^

Thus, when a **TBADT** solution (2 × 10^–4^ M in CH_2_Cl_2_/C_6_H_12_/CH_3_CN 6:3:1) was irradiated at 325 nm with a 250 W Xe lamp (equipped with a grating monochromator), the formation of the KA–oil was observed with a quantum yield of 0.35.^480^ It is important to mention that a great influence of the oxygen pressure on the final product distribution was observed. In fact, while the formation of the alcohol was not affected when increasing O_2_ pressure from ∼0.03 to 1 atm, the percentage of ketone in the mixture increased considerably. Thus, while at 0.03 atm the alcohol/ketone ratio was found to be 7:3, at 1 atm the ratio was fully reversed (ca. 3:7).^480^ As stated by the authors, the distribution of the final products depends on a delicate interplay of the involved radical species. In particular, at low oxygen concentration, the reduced photocatalyst interacts with O_2_ to afford H_2_O_2_ and eventually OH^•^. Radical recombination of the latter intermediate with the photocatalytically generated cyclohexyl radical affords cyclohexanol. In such a scenario, the formation of the ketone is proposed to be a marginal phenomenon. Conversely, at high pressure of oxygen, different pathways are stimulated, such as radical trapping of cyclohexyl radical by triplet oxygen to get the corresponding hydroperoxyl intermediate. The decomposition of such an intermediate, as also mentioned above, leads to the formation of a mixture of ketone and alcohol through radical chain autoxidation, with a preference for the former.

#### 3.2.3 Synthesis of Carbonyl Derivatives

**Scheme 64:**
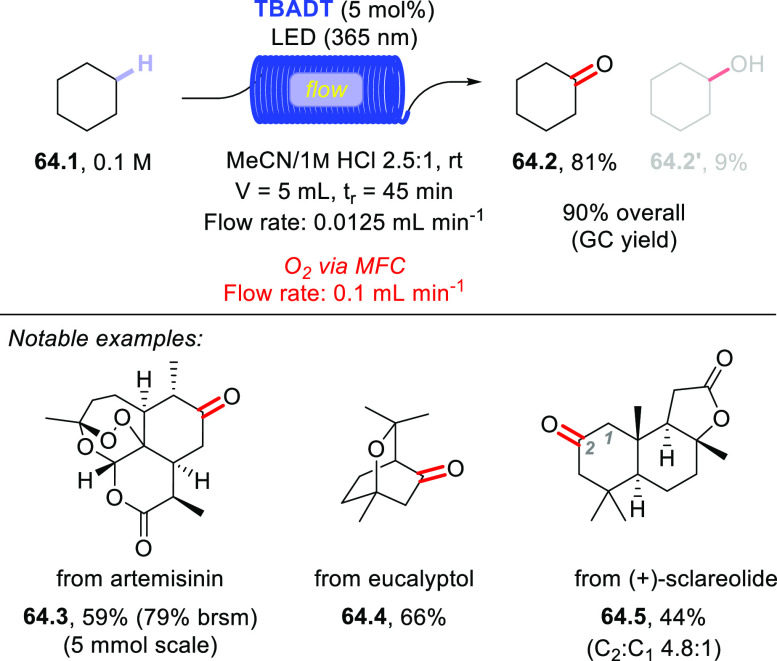
Molecular Oxygen as the Oxidant in the Selective CH_2_ to C=O Conversion

As mentioned in the previous paragraph, when **TBADT**-photocatalyzed HAT is carried out under an O_2_-rich atmosphere, the selectivity can be diverted toward the formation of the C=O double bond. The direct, 4-electron oxidation of a CH_2_ group into ketones has been achieved on a preparative scale by combining the use of **TBADT** (5 mol %) and O_2_ with a flow technology (V = 5 mL; PFA tubing, ø_in_ = 750 μm).^275^ Thus, a MeCN/1 M HCl 2.5:1 solution of cyclohexane (**64.1**, ∼0.1 M) and molecular oxygen were pumped through a PFA tubing coiled around a 3D-printed PLA cylinder and the mixture was irradiated with UV LEDs (λ = 365 nm). As a result, a cyclohexanone/cyclohexanol mixture (**64.2**/**64.2′** 9:1) was obtained in 90% overall yield from cyclohexane, outperforming the results obtained in batch. Interestingly, this methodology could be extended to natural scaffolds (**64.3**–**64.5**) and, for instance, allowed us to realize the oxidation of artemisinin to 9-artemisitone **64.3** with great efficiency and selectivity (59%, gram-scale) in a MeCN/DCM mixture (acid was omitted, Scheme 64).

**Scheme 65:**
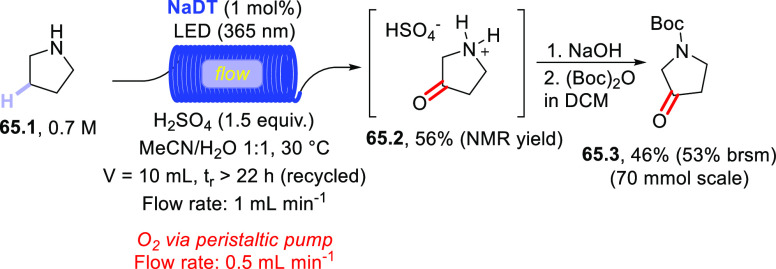
Gram-Scale Remote Oxidation of Pyrrolidine

In another instance, the use of **NaDT** for the remote oxidation of aliphatic amines was reported.^111^ Typically, protected amines undergo photocatalyzed HAT only at the α-to-N position. Building upon previous reports, however, it was envisaged that the protonation of amines could divert the HAT reactivity toward remote C–H bonds^98^ (see also the Introduction, Scheme 2). Thus, the use of flow technology enabled the conversion of pyrrolidine **65.1** to (protected) 3-pyrrolidinone **65.3** under mild conditions on a 5 g scale in 46% isolated yield in the presence of 1.5 equiv of sulfuric acid and molecular oxygen as the oxidant (Scheme 65). The initially formed ketoamine **65.2** was not stable enough for purification, so isolation was performed after derivatization, for example through Boc-protection to give **65.3**. The reaction could also be conducted in batch by adopting hydrogen peroxide as the oxidant.^111^

The direct oxidation of labile benzylic C–H bonds for the synthesis of carbonyl compounds was reported to take place under visible light. For example, **UrN** was used for the benzylic oxidation of indane and isochroman, respectively, to indanone and isochromanone in high yields (up to 87 and 80%) upon irradiation at 420 nm.^488^ Similarly, **UrP** was employed for the oxidation of toluene to benzaldehyde, with minor amounts of benzyl alcohol detected.^466^

Ketones can be smoothly prepared upon photooxidation of the corresponding alcohols via photocatalyzed HAT. Accordingly, **TBADT** was used to convert benzyl alcohols to the corresponding ketones in high yields on a 0.1 mmol scale.^489,490^ Molecular oxygen was used as the oxidant and was bubbled with a constant flow (20 mL min^–1^) in the solution. Thus, when an acetonitrile solution of 1-(4-methylphenyl)ethanol in the presence of 1 mol % of the PC_HAT_ was irradiated for 90 min, 82% conversion to the corresponding acetophenone was observed (>99% yield). Notably, when the very same PC_HAT_ was immobilized on TiO_2_, a competition between hydrogen atom transfer and single-electron transfer was observed and the conversion rate was halved (35% in 90 min).^489^ It is important to mention that, albeit homogeneous **TBADT** showed higher reaction rates, the heterogenized photocatalyst showed better chemoselectivity toward the α-to-O C–H bond when two distinguishable benzylic sites were present in the substrate, as demonstrated for 1-(4-ethylphenyl)ethanol.^489^

In another instance, **TBADT** immobilized on silica (10% w/w) promoted the oxygen-assisted chemoselective photooxidation of 1-pentanol or 3-pentanol to pentanal or 3-pentanone, respectively.^491^ When the alcohol (5 mM) was irradiated under O_2_ for 1 h at λ > 290 nm in the presence of the heterogeneous PC_HAT_ (8 g L^–1^), 90% mass balance was observed. Of note, no overoxidation products were detected in this case, contrary to what was observed under the same conditions when using homogeneous **TBADT**. The performances of homogeneous and heterogeneous **TBADT** were then directly compared, and the role of the matrix (silica) was evoked to explain the differences in reactivity. Indeed, the oxidation of 1-pentanol occurred 4-times faster when the PC_HAT_ was supported on SiO_2_ due to the positive effect of adsorption phenomena. Later on, this heterogeneous PC_HAT_ was also used for the oxidation of diols with similar results in terms of chemoselectivity.^492^ Similarly, silica-heterogenized **Ur** has been used for the oxidation of alcohols.^478^

**Scheme 66:**
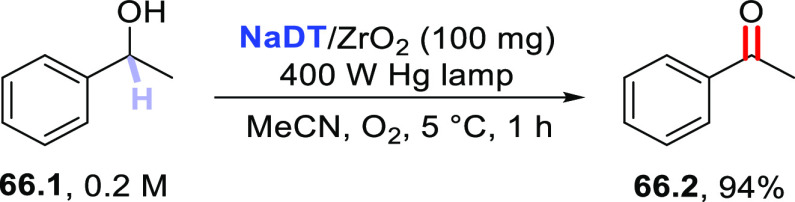
Heterogenized **NaDT**-Photocatalyzed Oxidation of Benzylic Alcohols

Intriguingly, a conceptually similar approach was reported for the oxidation of benzyl alcohols (and of aliphatic alcohols to some extent) by **NaDT** on a preparative scale.^493^ In detail, zirconia was used as a solid support for this PC_HAT_ (∼19% w/w) and the transformation occurred by irradiating a 0.2 M solution of the starting alcohol with a 400 W high-pressure Hg lamp using a cutoff filter (λ > 320 nm) for less than 2 h in most cases, delivering the expected ketone in >90% yield. However, when benzyl alcohols bearing strongly electron-withdrawing substituents were used, longer irradiation times were required (up to 4 h). As an example, the oxidation of 1-phenylethanol (**66.1**) occurred smoothly on a 2 mmol scale to give the corresponding acetophenone (**66.2**) in 94% yield after only 1 h of irradiation (Scheme 66). Notably, the use of the corresponding homogeneous PC_HAT_ led to decreased yields (68%). The photocatalyzed oxidation of **66.1** could be conducted on a 100 mmol scale in 3.25 h to deliver the expected ketone in 92% isolated yield. When the same methodology was applied to nonbranched benzylic alcohols, aldehydes were formed. Thus, benzyl alcohol was smoothly converted to benzaldehyde in 88% yield after 2 h of irradiation.

**Scheme 67:**
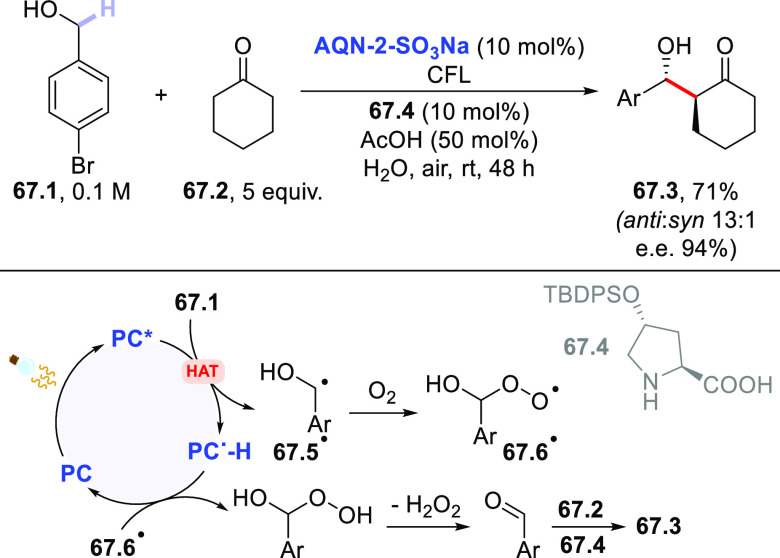
One-Pot Asymmetric Aldol Reaction Starting from Benzyl Alcohols under Aerobic Conditions

The photooxidation of benzyl alcohols to aldehydes under aerobic conditions has also been reported using water as a benign solvent.^494^ In particular, a tandem transformation was reported where this first step was triggered by **AQN-2-SO**_**3**_**Na** (10 mol %), followed by an asymmetric aldol reaction with ketones in the presence of a chiral pyrrolidine-based organocatalyst. Prolonged irradiation (48 h) and substoichiometric acetic acid (50 mol %) were needed to obtain full conversion of the starting materials. For example, (4-bromophenyl)methanol (**67.1**) was a competent substrate in the presence of cyclohexanone (**67.2**, 5 equiv), delivering product **67.3** in 71% yield with exquisite diastereo- and enantioselectivity. Benzyl alcohols bearing strong electron-donating substituents failed to give the expected products due to the diminished electrophilicity of the intermediate aldehyde (Scheme 67).

**Scheme 68:**
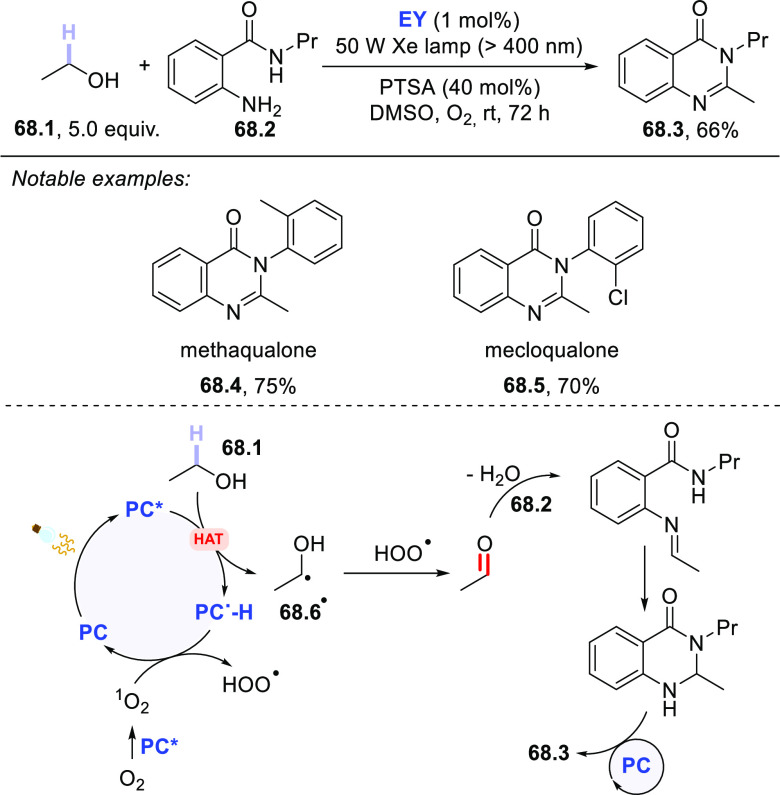
EY-Photocatalyzed Synthesis of Alkyl Quinazolinones via Oxidation of Primary Alcohols

The same PC_HAT_ was later used to promote the oxidation of both hydrocarbons and alcohols to the corresponding carbonyl compounds. For example, it was found that, upon irradiation with a 200 W white LED lamp, the water-soluble **AQN-2-SO**_**3**_**Na** (3 mol %) could be used to promote efficiently the oxidation of toluene to benzaldehyde (GC yield: 81%).^495^ It was observed that the transformation proceeded through the rate-determining formation of benzyl alcohol.^495^ In another instance, the use of **EY** as POC extended this transformation to primary alcohols, besides easily oxidizable benzyl alcohols, for the synthesis of quinazolinones in a one-pot fashion.^252^ As an example, when ethanol (**68.1**) was used as the substrate in DMSO in the presence of **EY** (1 mol %) and the mixture was irradiated for 72 h with a 50 W Xe lamp (cutoff > 400 nm) under O_2_ atmosphere, acetaldehyde was formed *in situ* and conveniently trapped by *o*-aminobenzamide **68.2** to give the expected product **68.3** in 66% yield (Scheme 68). Remarkably, this procedure was adopted for the synthesis of 2-alkylquinazolinones, including the sedative-hypnotic drugs methaqualone **68.4** and mecloqualone **68.5**.^252^

#### 3.2.4 Synthesis of Carboxylic Acids and Esters

**Scheme 69:**
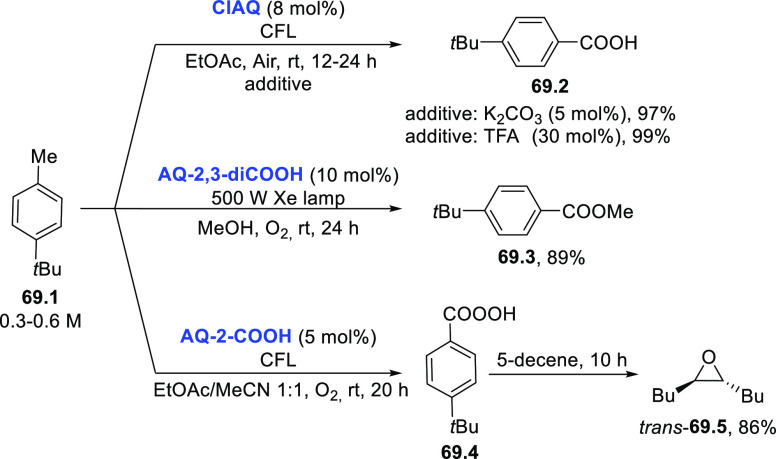
Anthraquinone-Photocatalyzed Benzylic Oxidation of Methylarenes

Photocatalyzed HAT was likewise exploited for the oxidation of methyl aromatics to the corresponding benzoic acids.^496^ As an example, when 4-*tert*-butyltoluene **69.1** was reacted under aerobic conditions in the presence of **ClAQ**, a mixture of the corresponding benzoic and perbenzoic acids was obtained. It was found that the use of either a base or an acid could steer the selectivity toward the acid. Accordingly, when a 0.3 M ethyl acetate solution of **69.1** was irradiated in the presence of **ClAQ** (8 mol %), the corresponding benzoic acid **69.2** was isolated in 97% and 99% yields when using K_2_CO_3_ or TFA as an additive, respectively (Scheme 69). A stepwise mechanism was unveiled, where the alkyl aromatic is first converted to the aldehyde via a hydroperoxide intermediate, which is in turn hydrated and then transformed into the acid by a similar photooxidative process.^496^

Later on, this strategy was used for the direct aerobic photo-oxidative synthesis of aromatic methyl esters starting from methyl aromatics.^241^ In detail, when methanol was adopted as the reaction solvent, the transient aldehyde was converted to the corresponding hemiacetal and, eventually, to the methyl ester. For example, **69.1** was converted to **69.3** in 89% isolated yield by irradiating a solution containing **AQ-2,3-diCOOH** (10 mol %) for 24 h in methanol (Scheme 69). Unfortunately, this strategy could not be extended to the synthesis of other alkyl carboxylates, since other alcohols, such as ethanol or propanol, were found to undergo uncontrolled oxidation when used as solvents.

Intriguingly, when **69.1** was reacted in the presence of **AQ-2-COOH** (5 mol %) in pure aerated ethyl acetate without any additive, the perbenzoic acid **69.4** was formed predominantly (88% overall ^1^H NMR yield, perbenzoic:benzoic acid ratio 2.8:1). This product could be in turn used in a one-pot fashion to epoxidize 5-decene to give **69.5** in 86% isolated yield when MeCN was used as cosolvent (Scheme 69).^497^

### 3.3 Formation of C–S Bonds

**Scheme 70:**
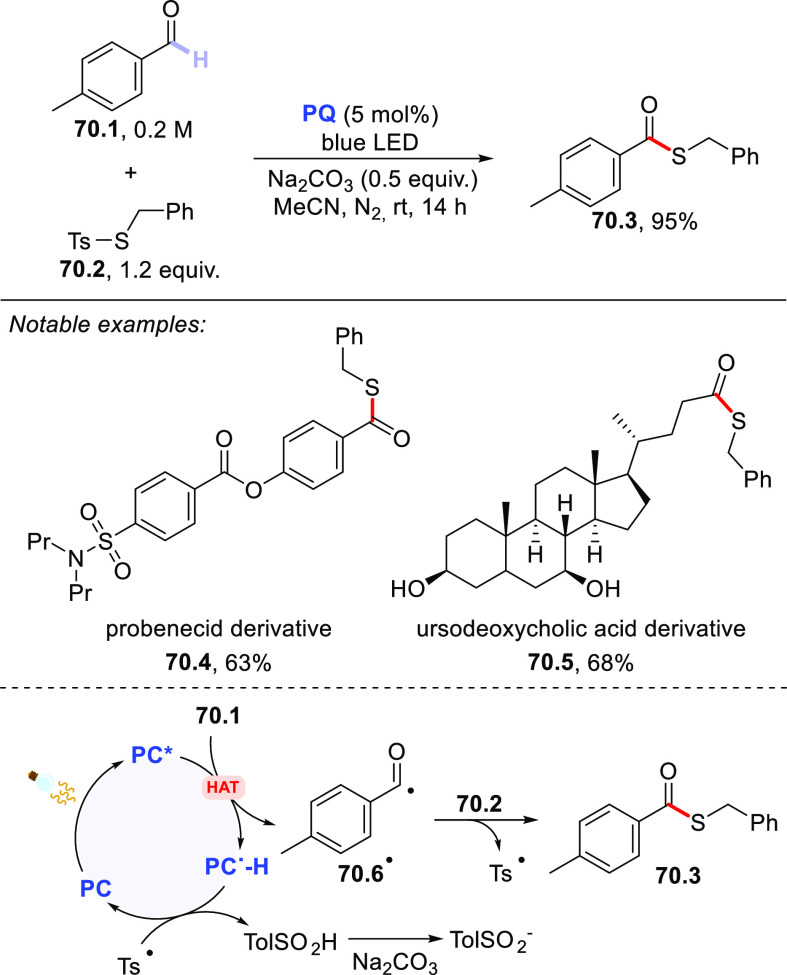
Photocatalyzed Thioesterification of Aldehydes

Photocatalyzed HAT was also employed to forge C–S bonds via addition of the photogenerated C-centered radicals onto a weak S–S single bond (see also section 4.3). In one instance, **PQ** was chosen as visible light-triggered PC_HAT_ (5 mol %) to promote the metal- and oxidant-free thioesterification of aldehydes.^244^ Thus, it was found that thiosulfonate **70.2** used in slight excess (1.2 equiv) was a suitable trap for the acyl radical generated via HAT from **70.1** (0.2 M) because of the high polarization of the S–SO_2_ bond (Scheme 70). Consequently, the generated sulfonyl radical accounted for the closure of the photocatalytic cycle, yielding product **70.3** in 95% yield after 14 h of irradiation under blue light. The presence of a base (Na_2_CO_3_, 0.5 equiv) was found crucial to neutralize the sulfinic acid developed during the reaction. Notably, this protocol could be extended to several structurally diverse aldehydes and thiosulfonate *S*-esters and could be adopted for the functionalization of complex biologically active molecules, such as probenecid and ursodeoxycholic aldehyde derivatives to give **70.4** and **70.5**, respectively.^244^

**Scheme 71:**
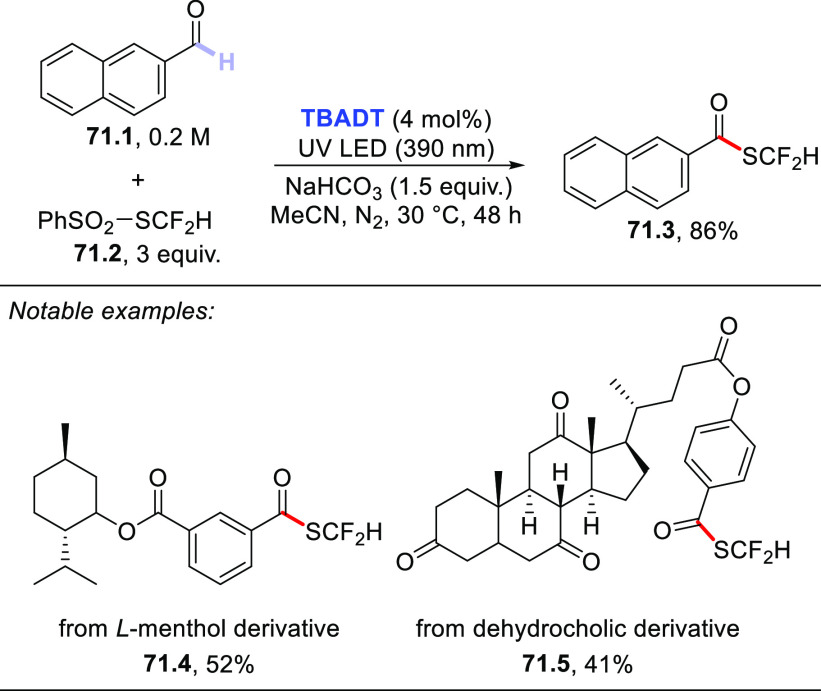
Difluoromethylthiolation of Aldehydes

Very recently, aldehydes functioned as substrates for a difluoromethylthiolation strategy triggered by photocatalyzed HAT (Scheme 71).^498^ Both aliphatic and aromatic aldehydes were activated by the excited state of **TBADT** (4 mol %) to form the corresponding acyl radicals that were in turn trapped by compound **71.2**, used in excess. Indeed, the expected difluoromethylthiolated product could be isolated in good yields on a 0.2 mmol scale. As an example, 2-naphthaldehyde **71.1** was converted to thioester **71.3** in 86% yield. As reported in Scheme 71, this methodology was exploited to functionalize biologically relevant molecules, such as *L*-menthol (product **71.4**) and dehydrocholic acid derivatives (product **71.5**).

**Scheme 72:**
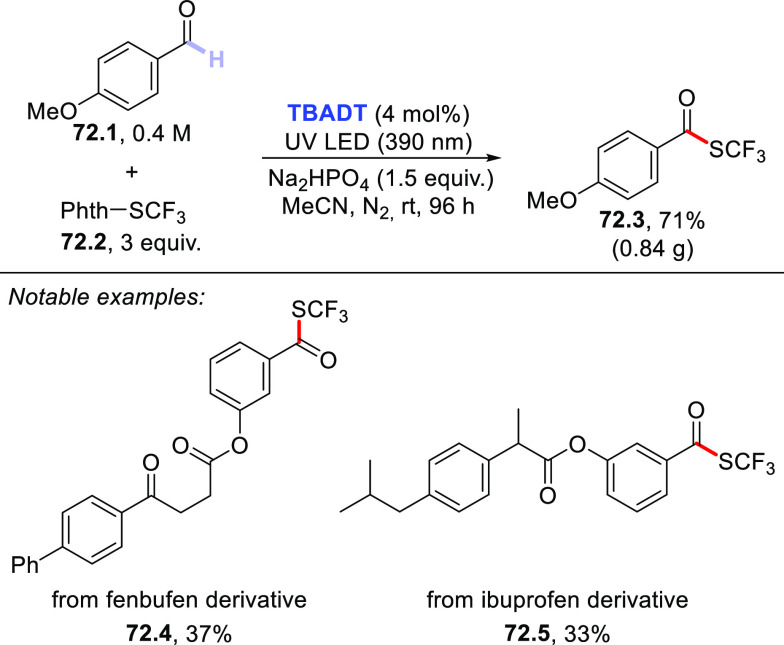
Trifluoromethylthiolation of Aldehydes

A conceptually similar procedure was reported for the trifluoromethylthiolation of aromatic aldehydes.^499^ For example, **TBADT** (4 mol %) was used to cleave the formyl C–H bond in *p*-anisaldehyde (**72.1**) and the acyl radical was readily trapped by *N*-(trifluoromethylthio)phthalimide (**72.2**) to afford the expected product (**72.3**, Scheme 72). Notably, the protocol proved robust and enabled this transformation on a gram-scale (5 mmol), as well as the functionalization of fenbufen and ibuprofen to deliver products **72.4** and **72.5**, respectively. It is perhaps important to mention that the authors found some limitations with substrates containing electron-withdrawing groups or substituents in the *ortho* position. They proposed the steric bulkiness of **TBADT** as the main reason for this diminished reactivity.^499^

**Scheme 73:**
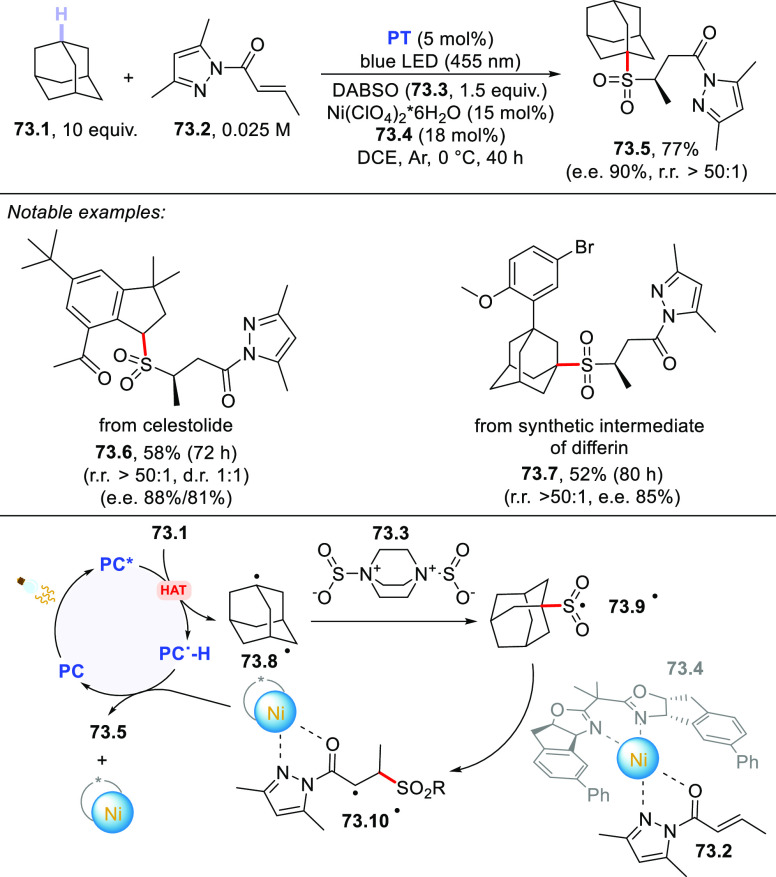
Three-Component Asymmetric Sulfonylation via HAT

Recently, a completely different strategy for the formation of C–S bonds has been disclosed. In this work, the authors made use of photocatalyzed HAT to generate C-centered radicals that were readily trapped by a SO_2_-surrogate (1,4-diazabicyclo[2.2.2]octane bis(sulfur dioxide), DABSO) to afford sulfonyl radicals. The latter intermediates were then exploited for a Ni-mediated enantioselective radical addition onto electrophilic olefins (Scheme 73).^500^ As an example, when adamantane (**73.1**) was irradiated in the presence of **PT** (5 mol %) under blue light (455 nm), the tertiary radical was generated. This intermediate was readily trapped by DABSO (**73.3**, 1.5 equiv) to forge the aimed-for C–S bond and afford a stable sulfonyl radical (e.g., **73.9**^**•**^). In the meanwhile, an asymmetric Ni complex was formed upon coordination of Ni^II^ by chiral ligand **73.4**; this Ni complex was responsible for imparting asymmetry on the following step via coordination of **73.2**. Finally, the sulfonyl radical added onto **73.2** to eventually afford the expected product **73.5** (77% yield, e.e. 90%, r.r. > 50:1).^500^ This protocol was likewise amenable to activated benzylic C–H bonds and to functionalize celestolide (to give **73.6**) and a synthetic intermediate of differin (**73.7**).

### 3.4 Formation of C–F Bonds

#### 3.4.1 Fluorination of C(sp^3^)–H Bonds

The introduction of a fluorine atom onto unactivated sites of organic molecules, such as strong aliphatic C(sp^3^)–H bonds, is a hot topic in medicinal and pharmaceutical sciences. In fact, fluorination makes a molecule more lipophilic, which results in ameliorated biological absorption and distribution. This transformation is of great importance in materials science too, where the incorporation of the halogen endows materials with a remarkable hydrophobicity.^501^ Photocatalyzed HAT has been widely used to promote this highly desirable transformation.^502^ This chemistry mainly relies on fluorinating agents containing a weak N–F bond (BDE = 61–63 kcal mol^–1^), such as *N*-fluorobenzenesulfonimide and Selectfluor.^503^

**Scheme 74:**
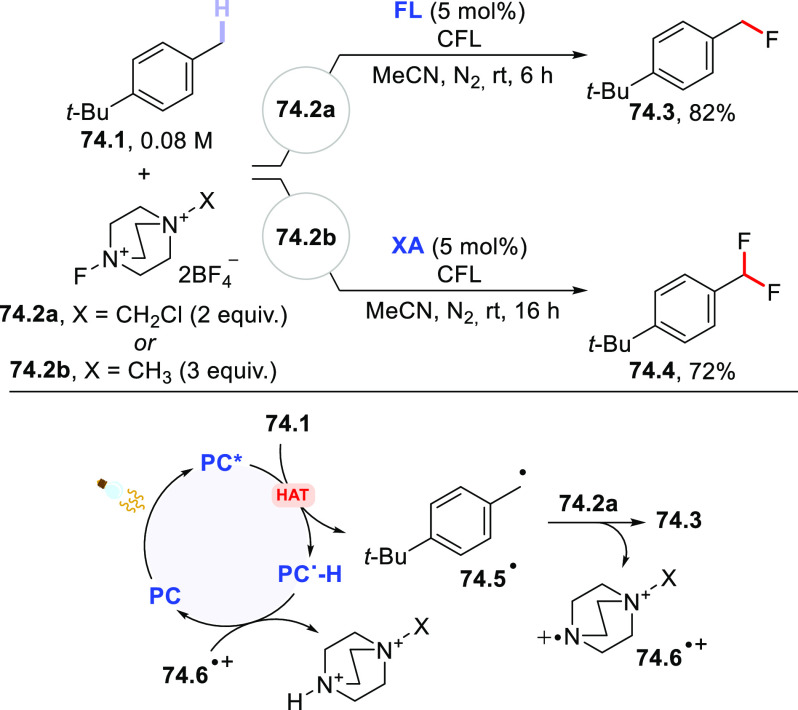
Diarylketone-Photocatalyzed Selective Benzylic Mono- and Difluorinations

In one of the earliest examples of this chemistry, the use of aromatic ketones for the mono- and *gem*-difluorination of benzylic C(sp^3^)–H bonds in the presence of Selectfluor was reported.^272^ In particular, when an acetonitrile solution of 1-(*tert*-butyl)-4-methylbenzene (**74.1**, 0.08 M) was irradiated with a CFL lamp in the presence of **FL** (5 mol %) and 2 equiv of Selectfluor (**74.2a**), the monofluorination at the benzylic site occurred in 82% yield to give compound **74.3** in 6 h (Scheme 74). However, when the reaction was performed in the presence of **XA** (5 mol %) and Selectfluor II (**74.2b**, 3 equiv) as PC_HAT_ and fluorinating agent, respectively, the *gem*-difluorinated product **74.4** was isolated in 72% yield after 16 h. From the mechanistic standpoint, the obtained organoradical was fluorinated by reaction with **74.2** to deliver the (di)fluorinated product and a *N*-centered radical cation, which is entrusted for the closure of the photocatalytic cycle.

**Scheme 75:**
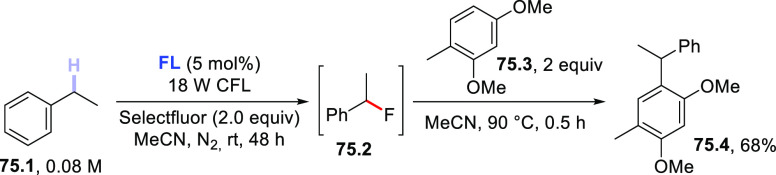
One-Pot Arylation of Unactivated Benzylic C–H Bonds

More recently, the above-mentioned procedure for the monofluorination of alkylaromatics was used to develop a one-pot, transition metal-free cross-dehydrogenative arylation of unactivated benzylic C–H bonds (Scheme 75).^504^ In particular, **FL** was used to trigger the halogenation step, while the so-formed benzyl fluorides (**75.2**) were used as electrophilic partners in a nucleophilic substitution with electron-rich (hetero)arenes (e.g. **75.3** to give **75.4**).^504^

**Scheme 76:**
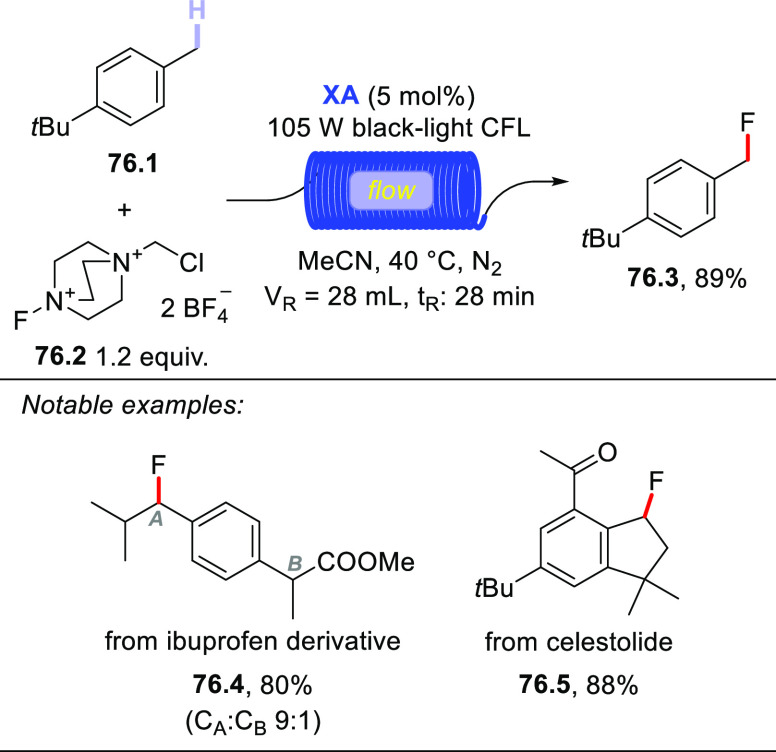
Photocatalyzed Benzylic Fluorination under Flow Conditions

In a related example, the same monofluorination reaction was performed under flow conditions by circulating an acetonitrile solution of **76.1** through a transparent FEP tubing coiled around a glass cylinder (V = 28 mL, flow rate = 1 mL·min^–1^) containing a 105 W CFL.^286^ Under these conditions, it was found that **XA** (5 mol %) could perform the monofunctionalization of the benzylic site to get **76.3** in 89% yield with a residence time of 28 min (temperature of the system = 40 °C). This reaction manifold was adapted also to the fluorination of drugs, such as celestolide and ester-protected ibuprofen (Scheme 76). As for celestolide, milder conditions were required (flow rate = 3 mL min^–1^, t_r_ = 9 min, temperature: 25 °C) to get the corresponding fluorinated compound in 88% yield and the reaction could be conveniently scaled up with comparable efficiency (87% yield). Contrarily, in the case of ibuprofen, “harsher” conditions were needed (flow rate = 1 mL min^–1^, t_r_ = 28 min, temperature: 60 °C) to get the expected product in 80% yield, with the secondary benzylic C–H position being almost exclusively functionalized (>90% selectivity).

**Scheme 77:**
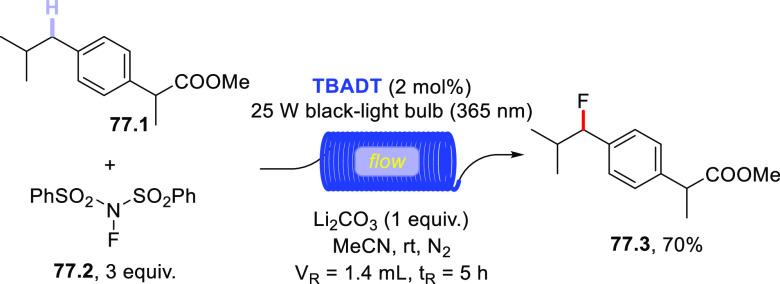
TBADT-Photocatalyzed Benzylic Fluorination in Flow

In another case, the benzylic fluorination of alkyl aromatics was achieved in the presence of **TBADT** and NFSI (**77.2**) as the fluorinating agent.^505^ Thus, ibuprofen methyl ester **77.1** was smoothly functionalized at the less-hindered benzylic position in 75% yield. It is important to stress that, when a flow apparatus was adopted (V = 1.4 mL, FEP tubing wrapped around a blacklight blue lamp; λ = 365 nm), the reaction was significantly sped up, from 24 h required in batch to only 5 h (70% yield, Scheme 77). Hydrolysis of the fluorinated ester gave fluoroibuprofen, whose metabolic stability in human and rat microsomes was evaluated to be somewhat higher compared to the parent drug.

Similarly, **DBS** was used to promote the selective fluorination of phenylalanine-like residues in amino acids and short chain peptides at the benzylic site. In this case, Selectfluor was employed as the fluorinating agent while a 14 W CFL was used as the light source.^243^

**Scheme 78:**
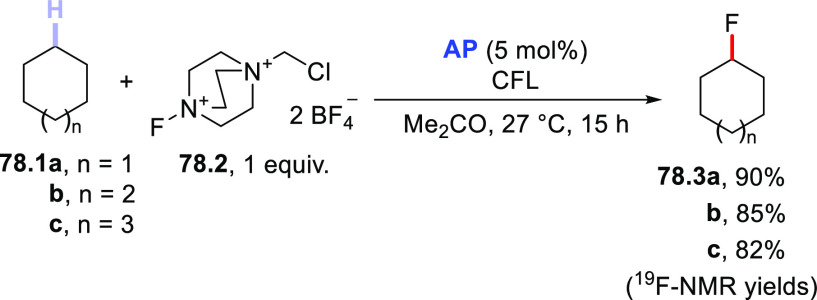
Photocatalyzed Preparation of Fluorocycloalkanes

Photocatalyzed HAT can be conveniently used also for the fluorination of strong aliphatic C–H bonds. In one case, the fluorination of cycloalkanes took place making use of aromatic ketones such as **AP**, which was effective in the C–H to C–F bond conversion upon irradiation with a commercially available CFL (Scheme 78). For example, the fluorination of cyclohexane, cycloheptane, and cyclooctane **78.1a–c** occurred in 90%, 85%, and 82% ^19^F NMR yield, respectively.^224^

**Scheme 79:**
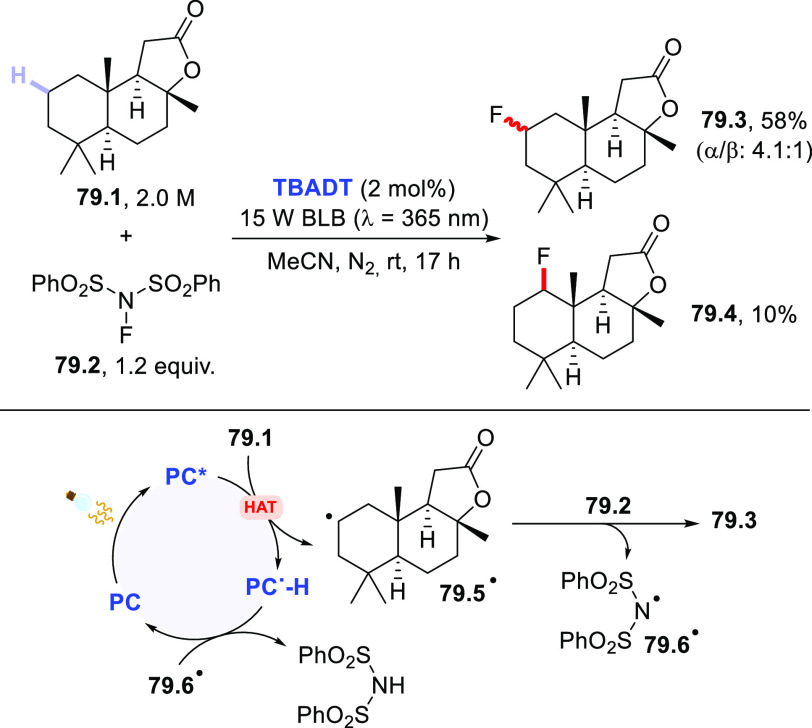
Photocatalytic Fluorination of Unactivated C–H Bonds

Another convenient PC_HAT_ that can be used for the fluorination of strong, unactivated C–H bonds is **TBADT** (Scheme 79).^506^ In particular, this polyoxometalate has been exploited for the fluorination of a wide array of compounds, including biologically valuable molecules such as (protected) amino acids and sclareolide (**79.1**). Thus, when an acetonitrile solution of **79.1** was irradiated for 17 h in the presence of NFSI (**79.2**, 1.2 equiv) and **TBADT** (2 mol %) at 365 nm (15 W black light bulbs), a mixture of the 8- and 9-fluorinated adducts was obtained (68% overall yield, ratio **79.3/79.4** 5.8:1). Interestingly, the selectivity enabled by this system is analogous to that previously reported adopting manganese porphyrins in the presence of fluoride anion.^507^

**Scheme 80:**
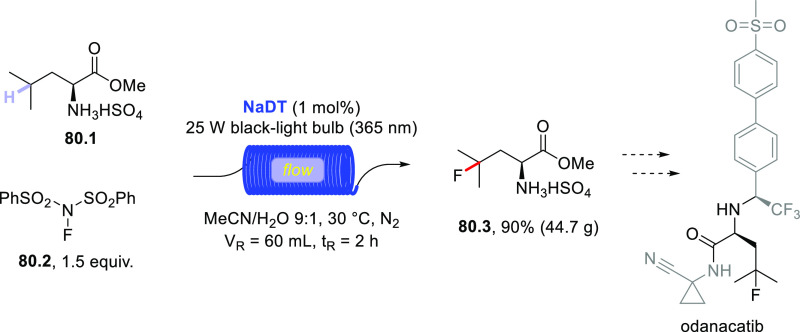
TBADT-Photocatalyzed Fluorination of Leucine in Flow

In this context, a similar strategy was adopted for the functionalization of the methine site in leucine. Thus, **NaDT** was found useful for the selective fluorination of γ-leucine methyl ester (**80.1**), a key intermediate on the route to odanacatib, a potent inhibitor of cathepsin K. Intriguingly, this transformation was carried out under flow conditions and could be easily scaled up, to get ∼45 g of product (90% yield, Scheme 80).^508^

The same procedure was used to achieve the site-selective ^18^F-fluorination of unprotected amino acids^273^ and peptides,^509^ which has an intrinsic value for positron emission tomography (PET) imaging. Later on, the same fluorination concept was adopted for the labeling of ZJ-43 analogues, potent binders for PSMA (prostate specific membrane antigen), that are overexpressed in the case of prostate cancer. Parent ZJ-43 could not be efficiently labeled (<12%) with ^18^F; however, the addition of pendant ammonium groups was found to accelerate the functionalization (>46% yield).^510^

**Scheme 81:**
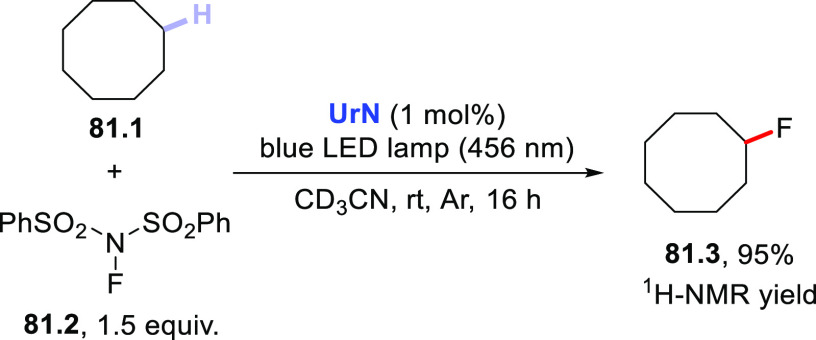
Visible Light Fluorination of Cyclooctane

Quite recently, the **UrN** photocatalyst has been used for the fluorination of cycloalkanes under visible light irradiation. Thus, a deuterated acetonitrile solution of cyclooctane (**81.1**) was irradiated in the presence of **UrN** (1 mol %) and 1.5 equiv of NFSI (**81.2**) for 16 h under inert atmosphere to afford fluorocyclooctane (**81.3**) in 95% ^1^H NMR yield (Scheme 81).^284^

#### 3.4.2 Fluorination of C(sp^2^)–H Bonds

**Scheme 82:**
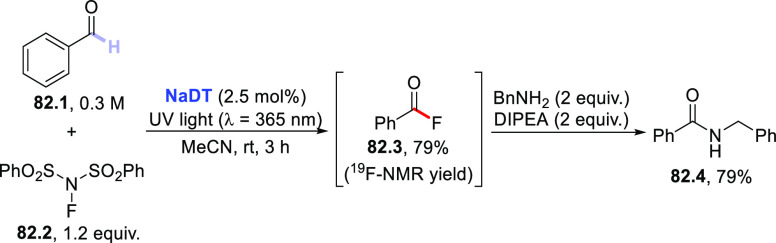
Synthesis of Acyl Fluorides from Aldehydes

The fluorination of aldehydes via the activation of the formyl C(sp^2^)–H bond has been sparsely reported.^511^ Here, the capability of **DT** to generate acyl radicals from aldehydes (**82.1**) was exploited. These intermediates were in turn trapped by NFSI to get the corresponding acyl fluorides (**82.3**, Scheme 82). Since acyl fluorides were found to be unstable in several cases, they were treated *in situ* with benzylamine to get the corresponding amides (**82.4**). For example, when an acetonitrile solution of benzaldehyde **82.1** (0.3 M) was irradiated at 365 nm in the presence of **NaDT** (2.5 mol %) and NFSI (**82.2**, 1.2 equiv), the corresponding acyl fluoride **82.3** was formed in 79% ^19^F NMR yield. After irradiation, the addition of 2 equiv of benzylamine allowed isolation of benzamide **82.4** in 79% yield. This reactivity was extended to several aliphatic aldehydes as well. Notably, **82.4** could be obtained in 53% isolated yield starting from benzyl alcohol in the presence of a higher amount of NFSI (2.5 equiv) in a one-pot fashion.^511^

### 3.5 Formation of C–Cl Bonds

Similarly to what was shown in the previous section for C–F bond formation, C–Cl bonds can also be formed via photocatalyzed HAT. In these cases, electrophilic reactants containing a weak N–Cl bond are exploited, with one typical example being *N*-chlorosuccinimide (NCS).

**Scheme 83:**
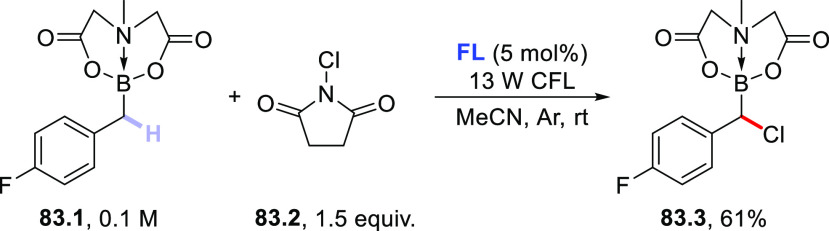
Chlorination of Benzyl MIDA Boronates

In one instance, the halogenation of α-to-boron C(sp^3^)–H bonds in benzyl *N*-methyliminodiacetyl (MIDA) boronates was reported.^512^ Thus, the authors started off by studying the bromination of said bonds and found an effective photochemical way to promote reactivity. In particular, the direct irradiation of a solution containing the boronate ester and *N-*bromosuccinimide (NBS) under inert atmosphere with a 13 W CFL led to the expected products. However, this protocol failed when replacing NBS with NCS to perform chlorination of C–H bonds; intriguingly, the authors found that the addition of 5 mol % of **FL** restored reactivity (Scheme 83). Upon mechanistic investigation, they found that **FL** acted as a photocatalyst for HAT. Interestingly, by replacing NCS with Selectfluor, the authors were able to achieve the fluorination of α-to-boron C(sp^3^)–H bonds.^512^

**Scheme 84:**
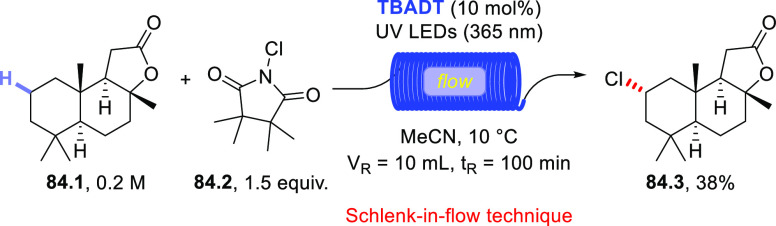
Chlorination of (+)-Sclareolide via a Schlenk-in-Flow Approach

In another instance, a similar chlorine source was used for the chlorination of (+)-sclareolide (**84.1**) under flow conditions (Scheme 84).^513^ Interestingly, the authors designed a “Schlenk-in-flow” technique for the safe handling of oxygen- and moisture-sensitive reagents, where argon is used instead of solvent to drive reagents through the tubing. Doing so, distribution phenomena are suppressed, which allowed saving both reagents and solvent. The photocatalyzed chlorination of **84.1** was one of their benchmark reactions: when an acetonitrile solution of **84.1** was pumped through the photoreactor (V_R_ = 10 mL, t_R_ = 100 min) in the presence of **TBADT** (10 mol %) and **84.2** (1.5 equiv) under UV light (365 nm), 38% of the chlorinated product **84.3** was obtained on a gram scale.

### 3.6 Formation of C–D Bonds

Deuterium labeling has a range of applications, including the investigation of reaction mechanisms, drugs metabolism and distribution, as well as in spectroscopy. Accordingly, it is no surprise that photocatalyzed HAT was investigated as a direct and unique platform to access the formation of the C–D (D: deuterium) bond by the so-called hydrogen isotope exchange (HIE).^514−517^

In this frame, two similar approaches have been published very recently. In both cases, the photogenerated radicals (**TBADT** as the PC_HAT_) were quenched by an aromatic thiol (triisopropylbenzenethiol, TIPSH) present in catalytic amounts and involved in an acid/base equilibrium with D_2_O. While in one instance the deuteration of the formyl C–H bond in both aromatic and aliphatic aldehydes was achieved,^518^ in the other one the methodology was extended to the deuteration of benzylic sites and tertiary C(sp^3^)–H bonds, besides aldehydes.^519^

**Scheme 85:**
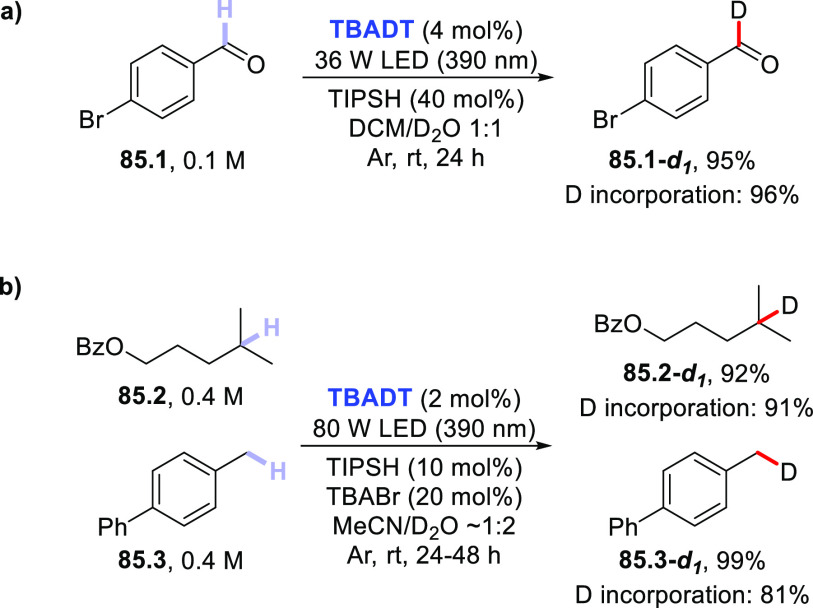
Photocatalyzed Hydrogen Isotope Exchange (HIE)

In the former case, 4-bromobenzaldehyde **85.1** was deuterated in 95% yield (D incorporation: 96%) by means of the **TBADT**/TIPSH system (used in 4 and 40 mol %, respectively) in DCM/D_2_O 1:1 upon irradiation for 24 h (Scheme 85a).^518^ In the latter case, the deuteration could be extended to benzylic sites and tertiary C(sp^3^)–H bonds simply by adding a phase transfer catalyst (TBAB, tetrabutylammonium bromide). For example, **85.2** and **85.3** were functionalized in 92% (D incorporation: 91%, 24 h) and 99% (D incorporation: 81%, 48 h) yield, respectively (Scheme 85b).^519^

## 4 Formation of Other Bonds

Photocatalyzed HAT has been likewise employed for the generation of heteroradicals, including P-, Si-, and S-centered radicals.

### 4.1 Formation of P–C Bonds

**Scheme 86:**
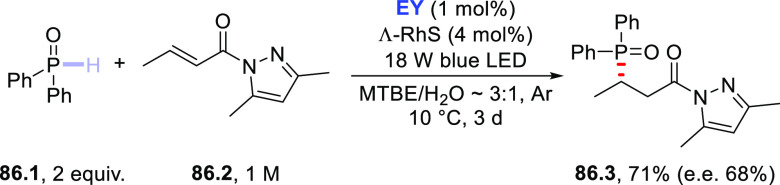
Enantioselective Formation of a P–C Bond

As for P–C bond formation, the enantioselective conversion of hydrophosphine oxide **86.1** to the phosphine oxide **86.3** via **EY**-photocatalyzed HAT has been reported (Scheme 86).^390^ In particular, when a solution of amide **86.2** (1 M) in MTBE/H_2_O ∼ 3:1 was irradiated with a blue LED in the presence of **EY** (1 mol %) and **86.1** as the H-donor, **86.3** was formed in 71% yield (e.e. 68%). As mentioned before (see Scheme 19d), the enantioselectivity was entrusted to a chiral Rh complex (4 mol %). Unfortunately, the reaction required a long time to go to completion (3 days).

Very recently, **EY** was used to promote the formation of a P–C bond starting from hydrophosphine oxide as H-donor and *N*-(hetero)arylsulfonyl propiolamides. As already mentioned above (see Scheme 45 in section 2.5), this transformation proceeded through a cascade involving 1,4-addition of the P-centered radical onto the C≡C triple bond of propiolamide, Smiles rearrangement, and 5-*endo*-trig cyclization.^437^ In a very recent report, *N*-aminopyridinium salts have been used as radical traps for photogenerated P-centered radicals (**AQ** as the photocatalyst), resulting in the pyridylation of a handful of hydrophosphine oxides.^416^

### 4.2 Formation of Si–X Bonds (X = Cl or C)

**Scheme 87:**
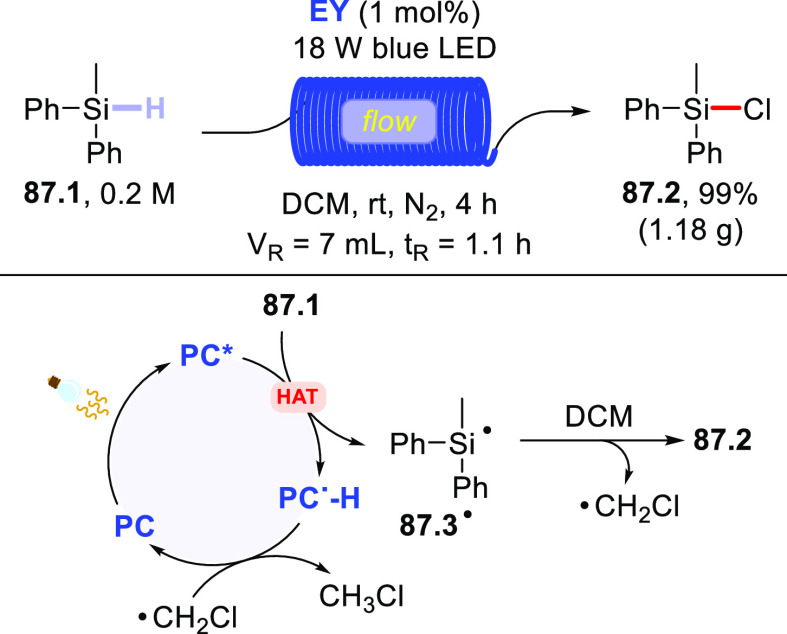
Photocatalyzed Synthesis of Chlorosilanes

Photocatalyzed HAT has been used also for the formation of Si–X bonds (X = Cl, C). This strategy requires that a Si–H bond is photocatalytically cleaved, which is easily predicted by the enhanced electropositivity of silicon compared to carbon. This feature contributes to make the H atom to be abstracted more hydridic.^248^ The forging of the Si–Cl bond gives access to chlorosilanes, currently used for the protection of alcohols and acids. The reaction occurred under visible light irradiation (**EY** as the PC_HAT_) and relied upon DCM in the double role of chlorinating agent and solvent. The range of applicability of this transformation is remarkably broad, and almost all chlorosilanes were isolated in quantitative yield. Notably, flow chemistry allowed successfully performing the process up to the gram scale. For example, when hydrosilane **87.1** (0.2 M) was irradiated with **EY** (1 mol %) in DCM with an 18 W blue LED strip, the corresponding chlorinated product **87.2** was obtained quantitatively after 4 h. By means of the flow technique (HPFA tubing, V = 7 mL, flow rate ∼ 0.1 mL·min^–1^), the same yield was achieved with a residence time of 1.1 h and 1.18 g of **87.2** was produced after 4 h (Scheme 87).^248^

**Scheme 88:**
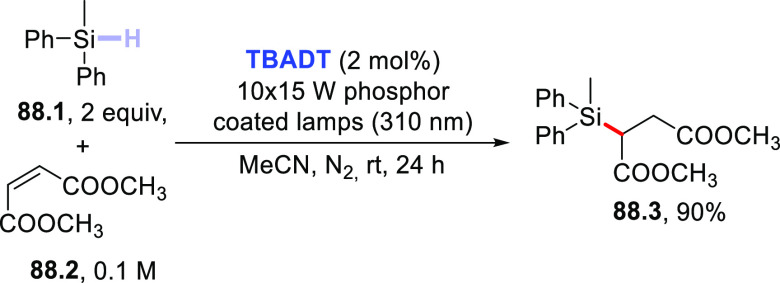
TBADT-Photocatalyzed Hydrosilylation of Electron-Poor Olefins

As for the formation of the Si–C bond, a handful of reports describe the use of **TBADT** for the activation of the Si–H bond (Scheme 88).^277,378,386,520^ Thus, when an acetonitrile solution of methyldiphenylsilane **88.1** was irradiated with UV light (310 nm, 10 × 15 W phosphor-coated lamps) for 24 h in the presence of **TBADT** (2 mol %), the expected Si-centered radical was generated via HAT.^520^ This intermediate was readily trapped by dimethyl maleate (**88.2**) to afford the corresponding silylated compound in excellent isolated yield (90%), even though it should be noted that 28% yield was observed in the absence of the PC_HAT_. The use of transient absorption spectroscopy and steady-state EPR experiments served to prove the nature of the activation step. Interestingly, when silanes containing more labile Si–H bonds (e.g., tris(trimethylsilyl)silane) were used, the reaction proceeded even in the absence of the photocatalyst due to radical chain processes. On the other hand, when trialkylsilanes were used, the competitive activation of the α-to-Si C–H and Si–H bonds was observed, thus generating a mixture of C- and Si-functionalized products.^520^

### 4.3 Formation of S–C Bonds

**Scheme 89:**
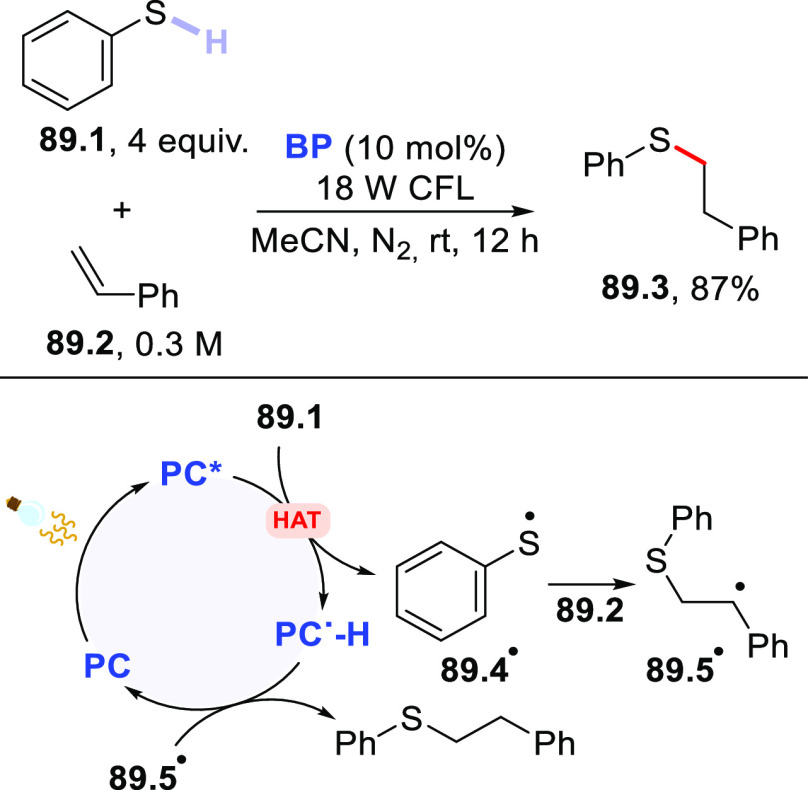
BP-Photocatalyzed S–C Bond Formation

The formation of S–C bonds can be achieved through the addition of S-centered radicals generated via HAT onto electron-rich olefins such as styrenes. In one case, a thiol–ene process has been reported by using **BP** (10 mol %) as the PC_HAT_, which allowed formation of sulfide **89.3** in 87% yield after 12 h of irradiation of a mixture of thiophenol (**89.1**) and styrene (**89.2**) with a 18 W CFL (Scheme 89).^236^

Quite recently, an asymmetric manifold for the formation of the S–C bond has been proposed.^390^ Therein, a sulfinic acid was activated via HAT by **EY** to get the corresponding S-centered radical. The latter intermediate was trapped by α,β-unsaturated amides through a radical addition manifold to forge the desired S–C bond. Notably, a Ru-based chiral Lewis acid was used to coordinate the amide with a dual objective: favoring the radical addition step and introducing asymmetry.^390^

Finally, given the importance of the trifluoromethylthio (−SCF_3_) moiety in medicinal chemistry, a methodology based on HAT has been recently proposed for the trifluoromethylation of β-ketodithioesters under visible light irradiation.^251^ Thus, when methyl 3-oxo-3-(3,4,5-trimethoxyphenyl)propanedithioate was irradiated with blue light in the presence of **EY** (2 mol %) and sodium triflinate, the corresponding trifluoromethylthiolated product was obtained as a single diastereomer (*Z*) in 89% yield after 15 h. It is perhaps worth mentioning that the reaction worked to some extent (37% yield) also in the absence of the PC_HAT_.

## 5 Miscellanea

This section gathers examples that do not fit into any of the categories above. In particular, net-oxidative processes based on a HAT step such as dehydrogenation and dehydroformylation reactions, as well as reactions based on a C–C oxidative cleavage, are described.

Seminal examples of such processes have been reported in the mid 1980s/early 1990s and were based on the use of polyoxometalates as PCs_HAT_, with particular focus on the **DT** anion; albeit, the reactions were usually stopped at low conversion of the starting mixtures.^200^ Thus, the dehydrogenation of a library of organic substrates (mostly alkanes and alcohols) based on photocatalyzed HAT has been reported.^321,324,329,521,522^ Indeed, specific conditions have been found to deliver the expected products with a high quantum yield (approaching unity),^522^ as well as to favor the formation of nonthermodynamic alkenes from alkanes.^329^ Furthermore, it was demonstrated that photocatalyzed HAT can also be exploited to trigger the epimerization of C–H bonds (e.g., in decalone derivatives).^324,521^

**Scheme 90:**
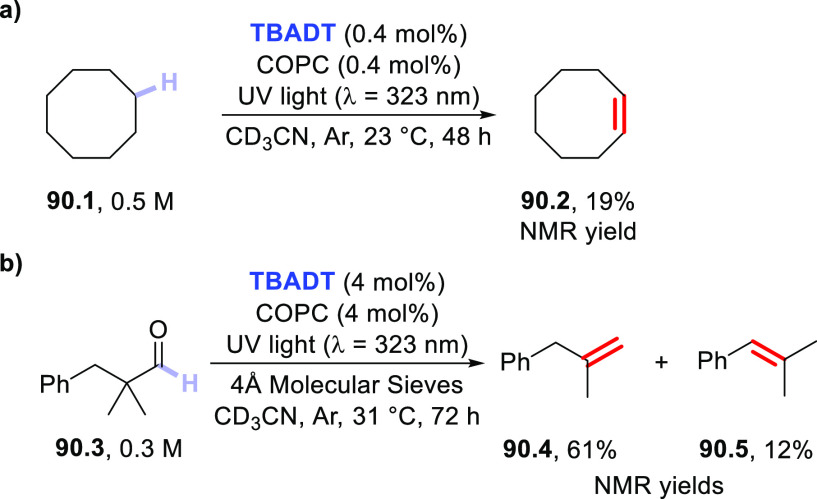
TBADT/Cobaloxime Dual-Catalytic Approaches for the (a) Dehydrogenation of Alkanes and (b) Dehydroformylation of Aldehydes

More recently, a dual-catalytic system consisting of **TBADT** (0.4 mol %) and cobaloxime pyridine chloride ([Co(dmgH)_2_(pyr)Cl], COPC; 0.4 mol %) was used for the preparative dehydrogenation of alkanes.^523^ This methodology was baptized “cooperative HAT”, since it involved the combination of photocatalyzed HAT and thermal HAT.^524^ In detail, the PC_HAT_ was responsible for the activation of a strong C(sp^3^)–H bond, for example in cyclooctane **90.1**, in what has been called a “hard” HAT step (Scheme 90a). Subsequently, the Co-based complex exerted an “easy” HAT from the α-position with respect to the radical site. The two catalytic cycles met when the reduced **DT** and the spent cocatalyst interacted to release a molecule of hydrogen gas. Ultimately, cyclooctene (**90.2**) was obtained in 19% NMR yield upon irradiation for 48 h. The very same procedure was also successfully applied to secondary alcohols, delivering the corresponding ketones in good yields.^523^

Later on, a dehydroformylation procedure via HAT has been developed.^525^ In particular, the same dual-catalytic system has been proposed to realize the decomposition of an aldehyde to an alkene, hydrogen gas, and carbon monoxide. Thus, aldehydes containing a nonenolizable quaternary α-carbon, such as 2,2-dimethyl-3-phenylpropanal **90.3**, were tested at first (Scheme 90b). **TBADT** (4 mol %) generated an acyl radical under UV light irradiation, that spontaneously underwent decarbonylation to give a tertiary radical. The latter intermediate was in turn intercepted by cobaloxime (COPC, 4 mol %), that triggered a further dehydrogenation step, with a preference toward the less substituted olefin (terminal olefin **90.4**). Aldehydes with an enolizable carbon α to the carbonyl group, however, were not competent substrates in this reaction.^525^

**Scheme 91:**
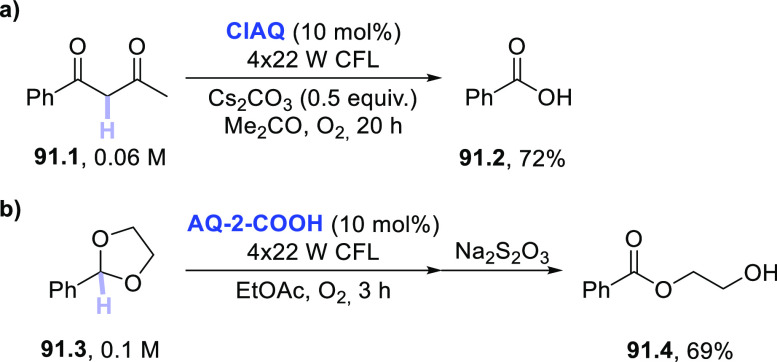
Aerobic Photooxidative Cleavage of (a) 1,3-Diketones and (b) 1,3-Dioxolanes

Another photocatalytic approach for the deconstructive cleavage of organic compounds via HAT dealt with the aerobic photooxidative cleavage of 1,3-diketones to give carboxylic acids.^526^ In such a case, **ClAQ** (10 mol %) was used to trigger a H-abstraction in benzoylacetones, such as 1-phenyl-1,3-butanedione **91.1** (Scheme 91a). Thus, when an acetone solution (0.06 M) was irradiated with four 22 W CFLs in the presence of oxygen and a base, the corresponding benzoic acid **91.2** was obtained in 72% yield after 20 h. The proposed mechanism underlying this transformation is quite complicated and proceeds through the formation of transient hydroperoxides and α-hydroxycarbonyl and α-dicarbonyl compounds.^526^ In a related instance, similar conditions were applied for the aerobic oxidative cleavage of cyclic acetals (Scheme 91b).^527^ As an example, irradiation of 1,3-dioxolane **91.3** in ethyl acetate solution in the presence of **AQ-2-COOH** (10 mol %) followed by quenching with sodium thiosulfate allowed synthesizing the corresponding ester **91.4** in good yield (69%).

## 6 Conclusions

It is apparent from the examples described in this review that the photocatalyzed hydrogen atom transfer approach has had and will have in future years a primary role in synthetic planning, in both academic and industrial settings. The versatility of the method is witnessed by the different types of bonds that can be forged, even though most of them are C–C bonds (see Table 2). In the majority of the examples reviewed, the reactions are carried out under mild conditions with no need of heating or aggressive conditions. In many instances, in the reactions developed all of the atoms of the reagents are incorporated in the desired products (100% atom economy).

**Table 2:** Use of the Diverse Classes of PCs_HAT_ in the Forging of New Bonds

**Bond formed/PCs_HAT_**	**Aromatic ketones**	**Anthraquinones**	**EY**	**DT**	**Ur**	**Sb-Oxo**	**TAC^•2+^**
**C(sp**^**3**^**)–C(sp**^**3**^**)**	**√**	**√**	**√**	**√**	**√**	**√**	
**C(sp**^**3**^**)–C(sp**^**2**^**)**	**√**		**√**	**√**			**√**
**C(sp**^**3**^**)–C(sp)**	**√**		**√**	**√**	**√**		
**C(sp**^**2**^**)–C(sp**^**3**^**)**	**√**		**√**	**√**	**√**	**√**	
**C(sp**^**2**^**)–C(sp**^**2**^**)**		**√**		**√**			
**C(sp**^**2**^**)–C(sp)**				**√**			
**C–N**	**√**			**√**			
**C–O**	**√**	**√**	**√**	**√**	**√**		
**C–S**	**√**		**√**	**√**			
**C–Cl**	**√**			**√**			
**C–F**	**√**			**√**	**√**		
**C–D**				**√**			
**Other bonds P–C, Si–Cl, Si–C, S–C**	**√**		**√**	**√**			

Table 2 gives an idea of the importance of the different classes of PCs_HAT_ used in connection with the bond they allow to build. Decatungstate salts (either **NaDT** or **TBADT**) are by far the most employed PCs_HAT_, and they are involved in all of the processes described. The capability of **DT**-based PCs_HAT_ to cleave even very strong bonds, such as the C–H bond in methane (BDE = 105 kcal/mol), showcases their impressive potentialities. Moreover, dual-catalytic approaches with Ni, Pd, Cu, and Co have extended even further the boundaries of the chemical space that can be explored with them. This colorless anion is robust, cheap, and easy to prepare; however, its chemistry is mainly restricted by the need for highly energetic UV light (<400 nm). Accordingly, in recent years a lot of work has been devoted to the discovery of visible light absorbing PCs_HAT_. Albeit colored polyoxometalates that can compete with **DT** are so far lacking, other classes of colored PCs_HAT_ have been tested, including oxo-porphyrins (**Sb-Oxo**), uranyl salts (**Ur**), or MOFs. Photoorganocatalysts appear as promising derivatives worthy to be further investigated. As for aromatic ketones, early examples dealt only with the use of colorless **BP**, but nowadays slightly colored diaryl ketones (e.g., **FL**, **DTX**, **PT**) or α-diketones (**PQ**) are preferred. Anthraquinone derivatives could have a more important role in the future; albeit, they have been used only in selected cases so far. **EY** is close to the ideal candidate as PC_HAT_ due to its wide availability and the performance showed, but its competitive role as PC_SET_ or singlet oxygen sensitizer must be carefully considered to avoid undesired side reactions. The use of alternative hydrogen abstractors, such as the excited state of the electrogenerated tris(amino)cyclopropenium radical dication (**TAC**^**•2+**^), is still in its infancy as it is the photoelectrochemistry approach.^528^

As apparent from Table 2, the forging of C(sp^3^)–C(sp^3^) bonds is widely investigated and a lot of conditions have been tested, making use of almost all of the classes of PCs_HAT_ available in conjunction with other catalysts, in turn resulting in interesting dual-catalytic applications. The Giese radical addition is the archetypal reactivity mode in this group.

However, in C–C bond formation limited efforts have been devoted to the formation of C(sp^2^)–C(sp^2^) and even less to C(sp^2^)–C(sp) bonds. The same holds for the construction of C–N and C–D bonds.

One of the main drawbacks of HAT reactions is that the homolytic cleavage of C–H bonds involves “nucleophilic” rather than “electrophilic” hydrogens, and it is quite rare to find a process that points to the generation (and ensuing reactions) of an electrophilic radical.^179^ However, quite recently, a new methodology based on radical polar crossover was exploited to reverse the polarity of said nucleophilic radical by generating a carbocation upon chemical oxidation.^463^ This concept finally made photocatalyzed HAT suitable to explore new chemical spaces, namely the world of polar chemistry. The possibility to choose the nucleophile to trap the carbocation sets up a fertile ground to develop new synthetic methodologies based on HAT.

The final comment is on selectivity in the hydrogen abstraction. As mentioned in the Introduction, there are several factors that may affect and direct the cleavage of a C–H bond out of all those present in the hydrogen donor. Dated examples typically dealt with hydrogen donors where all C–H bonds (e.g., in cyclohexane) were equivalent or the hydrogen abstraction was merely governed by the BDE (e.g., in tetrahydrofuran). Conversely, in recent times, very selective HAT reactions have been reported especially in the late-stage functionalization of complex molecules. This demonstrates that mastering the knowledge of all the factors explained in the Introduction allows chemists to develop surprisingly selective processes. Accordingly, further efforts toward a better understanding of these factors is needed to increase the potentialities of HAT reactions in the future. Finally, efforts on the development of hydrogen abstractors having peculiar shapes or a marked bulkiness as well as on the study of the cooperative effects in tuning the selectivity of the reaction are then crucial in this respect.

## Abbreviations

**ABP**: aminobenzophenone
**AP**: acetophenone
**AQ**: 9,10-anthraquinone
**AQ-2-COOH**: anthraquinone-2-carboxylic acid
**AQ-2,3-diCOOH**: anthraquinone-2,3-dicarboxyliic acid
**AQ-2-SO_3_Na**: anthraquinone-2-sulfonic acid sodium salt
**tBAQ**: 2-*tert*-butylanthraquinone
BLB: Black light bulb
Boc: *tert*-butoxycarbonyl
BOX: bisoxazoline
**BP**: benzophenone
BPO: benzoyl peroxide
**BPSS**: disodium benzophenondisulfonate
Brsm: based on remaining starting material
**CFL**: compact fluorescent lamp
**ClAQ**: 2-chloroantraquinone
COPC: cobaloxime pyridine chloride
**DBS**: dibenzosuberenone
**DCBP**: 4,4′-dichlorobenzophenone
DCM: dichloromethane
**DDQ**: 2,3-dichloro-5,6-dicyano-1,4-benzoquinone
DIAD: diisopropyl azodicarboxylate
DIPEA: *N*,*N*-diisopropylethylamine
**DMBP**: 4,4′-dimethoxybenzophenone
**DT**: decatungstate anion
**DT-BPY**: [Cu_4_(BPY)_6_Cl_2_(W_10_O_32_)]·3H_2_O
**DTX**: 3,6-dimethoxy-9*H*-thioxanthen-9-one
**EY**: Eosin Y
**FL**: 9-fluorenone
FPT: freeze pump–thaw
HAT: hydrogen atom transfer
h.p.: high pressure
l.p.: low pressure
MFC: mass flow controller
m.p.: medium pressure
MTBE: *tert*-butyl methyl ether
**NaDT**: sodium decatungstate
PC: photocatalyst
P.E.: petroleum ether
POC: photoorganocatalyst
**PGA**: phenylglyoxylic acid
PSMA: Prostate specific membrane antigen
**PQ**: 9,10-phenanthrenequinone
**PT**: 5,7,12,14-pentacenetetrone
PTSA: *p*-toluenesulfonic acid.
**PYD**: 1,6-pyrenedione
**Sb-Oxo**: antimony-oxo tetra-(*p*-methoxyphenyl)porphyrin
SOLFIN: solar synthesis of fine chemicals
TBAB: tetrabutyl ammonium bromide
**TBADT**: tetrabutylammonium decatungstate
TBDPS: *tert*-butyldiphenylsilyl
TEA: triethylamine
TFA: trifluoroacetic acid
TIPSH: triisopropylbenzenethiol
Ts: tosyl
**TX**: thioxanthone
**Ur**: uranyl cation
**UrN**: uranyl nitrate hexahydrate
**UrP**: uranyl perchlorate
**XA**: xanthone

## Notes

The photocatalyzed cyanation of alkanes has been reported (compare section 2.4)^529^ The photocatalyzed acylation of crotonic acid^530^ and the alkylation of aldehydes with pyridinium salts^531^ to give ketones have been published (compare section 2.5). The decatungstate-photocatalyzed functionalization of [60]fullerene with lactones was devised (compare section 2.8).^532^ The photocatalyzed C(sp^3^)−H sulfinylation^533^ or C(sp^3^)−H/C(sp^2^)−H trifluoromethylthiolation^534^ for the forging of C−S bonds have been obtained (compare section 3.3).
